# Mechanical ventilation, hospitalization time, deaths and disability according to the variants of Guillain-Barré syndrome: systematic review and meta-analysis

**DOI:** 10.17843/rpmesp.2024.413.13509

**Published:** 2024-08-28

**Authors:** Silvana Ximena Castro Diaz, Luiza Pereira-Salto, Roger Vladimir Araujo Castillo

**Affiliations:** 1 Universidad Peruana de Ciencias Aplicadas, Lima, Peru. Universidad Peruana de Ciencias Aplicadas Universidad Peruana de Ciencias Aplicadas Lima Peru

**Keywords:** Guillain-Barre Syndrome, Acute Autoimmune Neuropathy, Acute Inflammatory Demyelinating Polyneuropathy, Mechanical Ventilation, Hospitalization, Intensive Care Units, Mortality, Disability Evaluation

## Abstract

**Objectives.:**

To determine the requirement and time to mechanical ventilation and Intensive Care Unit (ICU), hospitalization and hospitalization time, death and disability of the axonal variants of Guillain-Barré Syndrome (GBS) in comparison with the acute demyelinating variant in patients of all the ages.

**Materials and methods.:**

The systematic review that included patients with GBS. The exposure variable was the axonal variants and the comparator was acute inflammatory demyelinating polyneuropathy (AIDP). The outcomes were the requirement and time on mechanical ventilation (MV), requirement and time in the ICU, hospitalization time, disability and death. The NewCasttle-Ottawa Scale (NOS) was used to assess risk of bias. A meta-analysis was conducted to calculate mean differences and relative risks (RR) with their 95% confidence intervals (CI) using inverse variances and random effects models.

**Results.:**

Of the 3116 articles found, 46 met the selection criteria. The time on MV was 7.42 days (95% CI: 0.36 to 1.48) and the hospitalization time was 3.11 (95% CI: 0.73 to 5.49) days for the axonal variants. The axonal variants had a RR of 0.47 (95% CI: 0.24 to 0.92) for the requirement of MV in adults, but it was 1.68 (95% CI: 1.25 to 2.25) in children. There was a high statistical heterogeneity.

**Conclusions.:**

Axonal variants showed, on average, longer MV and hospitalization time, overall and by subgroups. A high MV requirement was found for axonal variants in children; it was lower for adults.

## INTRODUCTION

Guillain-Barré syndrome (GBS) is an autoimmune disease characterized by progressive motor weakness and decreased or absent reflexes. Respiratory muscles may be compromised, resulting in the need for mechanical ventilation (MV), which occurs in 20-30% of patients ^1^^,^^2^.

GBS includes the demyelinating variant, also called acute inflammatory demyelinating polyradiculoneuropathy (AIDP), which causes sensory and motor symptoms, autonomic dysfunction, pain and cranial nerve deficits; on the other hand the axonal variant includes acute motor axonal neuropathy (AMAN), which causes motor symptoms only; the acute axonal sensory-motor neuropathy (AMSAN) presents with both sensory and motor symptoms; acute sensory neuropathy (ASAN) causes demyelination of peripheral nerves; and patients with the Miller-Fisher syndrome (MFS) present ophthalmoplegia, ataxia and areflexia ^3^^,^^4^.

The worldwide incidence is 0.89-1.89 cases per 100,000 population per year, with children having the best prognosis ^5^^-^^7^. AIDP accounts for 90% of cases in Europe and North America. However, axonal variants are the most common in Asia, and Central and South America, accounting for 30-40% of cases ^8^.

The need for MV implies admission to the Intensive Care Unit (ICU) ^9^. Patients with an intubation time longer than 2 months took more time (up to 6 months) to recover the ability to walk ^10^. Mortality in people requiring MV varies between 8.3-20% ^11^^,^^12^.

AIDP has a better prognosis compared to the axonal variant, with the MV requirement being 10% and 38%, respectively. AMAN and AIDP have a faster recovery compared to AMSAN ^13^. The latter manifests with severe symptoms, prolonged MV use ^14^^)^ and longer hospitalization time ^15^. However, findings from previous publications are inconsistent, highlighting the need to systematically analyze the data, as there are only narrative reviews.

Although there is a significant percentage of patients who require MV, develop permanent sequelae, or die, it is unclear which variant is most associated with adverse outcomes. Therefore, it is important to collect information on the adverse outcomes associated with each variant to quantify the additional resources needed for these patients and to develop contingency plans, especially for outbreak situations. Given the above, the aim of the study was to determine the frequency and duration of adverse outcomes (MV requirement and time, ICU admission and time, hospitalization and hospitalization time, death, disability) of the axonal variants of GBS compared to the acute demyelinating variant in patients of all ages.

**KEY MESSAGES**

Motivation for the study. It is unclear which variant of Guillain-Barré syndrome is mostly associated with adverse outcomes.

Main findings. Variants had longer time in MV (7.42 days longer), and hospitalization (3.11 days longer). By subgroups, we found that children with the axonal variant had a greater use of MV; whereas, the demyelinating variant was more frequent among adults in MV.

Implications. Our results could be used for the implementation of new public health policies, allowing health personnel to have a better knowledge of the prognosis of each variant and the necessary resources to face future outbreaks.

## MATERIALS AND METHODS

### Study design

A systematic review was conducted to evaluate the frequency of adverse outcomes of axonal variants of GBS (AMAN, AMSAN, ASAN, MFS) compared to the acute demyelinating variant (AIDP), stratified for patients younger and older than 18 years of age. The Cochrane methodology was used for the systematic review of observational studies, and the PRISMA (Preferred Reporting Items for Systematic Reviews and Meta-Analysis) checklist was applied ^16^^,^^17^. The search and selection of articles was conducted between July and September 2020, and updated in March 2021.

### Eligibility criteria

The population were observational studies including patients with the diagnosis of GBS, according to the clinical diagnosis appearing in the medical records, or according to diagnostic criteria such as Brighton, Asbury and Cornblath, National Institute of Neurologic Disorders and Stroke (NINCDS), Hadden, Ho, ICD-10 and electrophysiological. Axonal variants were considered to be the exposure variable compared to demyelinating variants. The considered outcomes were MV requirement and time, ICU admission and time, hospitalization and hospitalization time, death and disability. Observational studies were included. Clinical trials, systematic reviews, narrative reviews and clinical practice guidelines were excluded. No studies were excluded by filters. When articles were not accessible or could not be found in a language other than English, Spanish or Portuguese, the author was contacted; the study was excluded if this was not possible.

### Search strategy and study selection

A bibliographic search was conducted in MEDLINE/Pubmed, LILACS/SciELO, Cochrane, Scopus, Web of Science and Google Scholar (Supplementary Material). The selected articles were registered in the Zotero reference manager, in which duplicate articles were eliminated.

### Data extraction

From each selected study we extracted in duplicate the following information: number of participants, number exposed to each variant of GBS, outcome measures for each exposure, and measures of association with their 95% confidence intervals (CI). Outcomes were: numerical (MV time, ICU time, hospitalization time, disability score) and dichotomous (ICU admission, MV requirement, death, disability). For numerical outcomes, means with standard deviation (SD) were reported. If medians with interquartile ranges (IQR) were reported, the median was considered as the mean and the IQR was divided by 1.35 to estimate the SD following the Cochrane Manual ^17^. The measure of association was the mean difference (MD) with its 95% CI; if not reported, the MD was calculated without the 95% CI. The absolute frequencies of each outcome according to exposure were used for categorical outcomes; relative risks (RR) with their 95% CI were used as measures of association, if it was not reported, the RR was calculated without the 95% CI.

### Risk of bias assessment

Each article was assessed twice for methodological quality using the Newcastle-Ottawa Scale (NOS) for cohorts and for case-control studies. The cohort version was used for cross-sectional studies or case series. The NOS has three domains: selection, comparability, and exposure ^18^, with scores from 0 to 9. Studies with scores under 7 points were considered to be at high risk of bias. An Egger funnel plot was performed to assess publication bias for results with at least 9 articles. The probability of publication bias was high when the p value was ≤0.05.

### Data synthesis

A meta-analysis was carried out using Review Manager 5.3 software (RevMan 5.3, Copenhagen, The Cochrane Collaboration) for results that included 3 or more studies. Random-effects models with inverse variances were used as a form of weighting. They were obtained by combining all studies for that outcome, and in different subgroups: studies combining children and adults, children only, and adults only.

The I^2^ test and the chi^2^ test were used to estimate the statistical heterogeneity of the studies. I^2^ values equal to or greater than 75% indicated high heterogeneity, on the other hand, results under 25% represented low heterogeneity.

### Ethical considerations

Systematic reviews used information from open-access databases and published texts that did not contain individual participant identifiers. Studies with ethical violations or retractions were not included. No studies were excluded on the basis of sex, race, religion, origin or language. The protocol of this systematic review was approved by the Ethics Committee of the Faculty of Health Sciences of the Peruvian University of Applied Sciences (UPC) with the code FCS-CEI/216-07-20 and FCS-CEI/345-05-21. In addition, it was registered in the international database PROSPERO with code CRD42020198653.

## RESULTS

After applying the search terms, 10,960 articles were obtained from the database. After eliminating duplicates, 3116 remained. We excluded 2996 articles by title and abstract, and 120 were left for full-text review. Fifteen were eliminated for not having the exposition, four for not presenting the outcomes, 33 for not analyzing the results by exposition, 18 because the full text could not be accessed and four were in a language other than Spanish, English or Portuguese.

Forty-six articles were finally included, published between 2003 and 2020. Twenty-seven were conducted in Asia, seven in Europe, five in Turkey, five in Latin America and two in Africa. Thirteen were prospective cohorts, 26 retrospective cohorts, five case series, one cross-sectional analytical study and one case-control.

There were 4585 patients of both sexes: 1780 of the axonal variants and 2805 of the demyelinating variant. Seventeen articles considered the axonal variant as a whole, 14 reported AMAN separately, 14 reported AMSAN separately, one reported ASAN and nine reported MFS. Overall, 12 articles included only adults, 21 only children, and 13 included both groups.

Regarding outcomes, 34 mentioned MV requirement as a discrete variable, and 8 measured MV time as a continuous variable in days. Only 1 reported ICU admission as a discrete variable, 5 reported ICU time as a continuous variable in days, and 17 reported hospitalization time as a continuous variable in days. For post-event disability, 13 reported Hughes scale as a continuous variable, and 13 dichotomized the scale using different cutoff points. Ten studies reported the proportion of deaths as a discrete variable; 7 reported deaths during hospitalization, one study at 3 months, one at 6 months, and one during a fluctuating follow-up period.

After applying the NOS, 31 articles were found to be at low risk of bias, with one being a case-control study. Funnel plots were made for hospitalization time, disability as a dichotomous variable, need for MV and death. These did not demonstrate possible publication bias for disability or death. The studies did not stay within the limits of the funnel for hospitalization time, but were symmetrically distributed on both sides. For MV, there appeared to be a bias toward publications with RR<1.0, favoring axonal variants, in studies conducted in adults.

Twenty-four studies had numerical outcomes, 11 reported means with SD, 10 reported means without dispersion measures, and 3 reported medians with IQR, which were transformed into SD. Articles without dispersion measures, the combined SD of the remaining studies in their corresponding group, were used in the meta-analysis, as described by Ma *et al.*^19^. Only one reported the MD with its 95% CI, the rest were calculated using the Review Manager 5.4 program and appear in the corresponding “Forest Plots”. Forty-two of 46 studies reported a dichotomous outcome with absolute frequencies. Only two studies reported measures of association with 95% CI, one article reported odds ratio (OR) and another RR. Meta-analyses were performed for each of the outcomes, except for ICU admission because only one study had this outcome.

**Figure 1:**
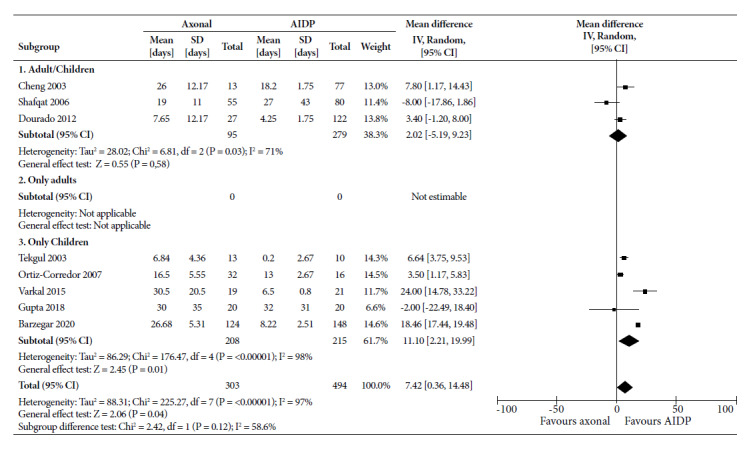
Forest Plot for mechanical ventilation time of the axonal variants compared to the demyelinating variant in patients with Guillain-Barré syndrome.

CI: confidence interval; AIDP: acute inflammatory demyelinating demyelinating polyradiculoneuropathy; ICU: Intensive Care Unit; SD: standard deviation.

For time in MV (Figure 1), MD was 2.02 (95% CI: -5.19 to 9.23) days longer for axonal variants in studies combining children and adults; whereas in studies with only children it was 11.10 days (95% CI: 2.21 to 19.99). This was not significant for any subgroup. The combination of the 8 studies showed a time of 7.42 days (95% CI: 0.36 to 14.48) in axonal variants, being significant, but with high statistical heterogeneity (I^2^ = 97%).

**Figure 2:**
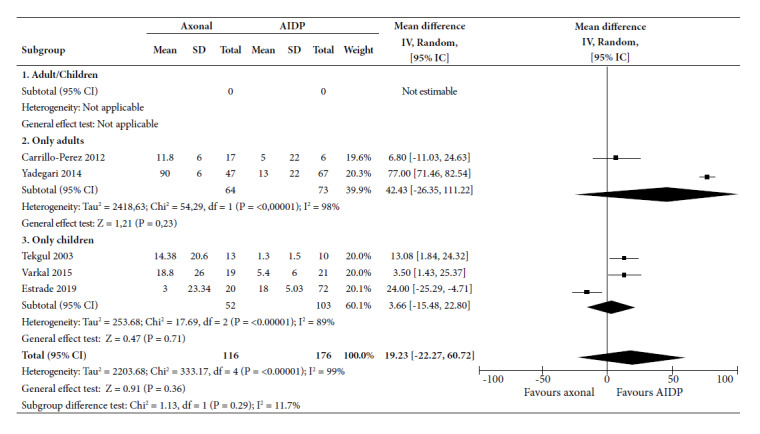
Forest Plot for time in Intensive Care Unit of axonal variants compared to demyelinating variant in patients with Guillain-Barré Syndrome.

CI: confidence interval; AIDP: acute inflammatory demyelinating demyelinating polyradiculoneuropathy; SD: standard deviation.

The mean length of stay in ICU (Figure 2) was 42.4 days (95% CI: -26.35 to 111.22) more for axonal variants only in adults; while in children only MD was 3.66 days (95% CI: -15.48 to 22.80); which was not significant. MD of 5 studies showed a time of 19.23 days (95% CI: -22.27 to 60.72) in axonal variants, which was not significant, and a high statistical heterogeneity (I2 = 99%).

**Figure 3:**
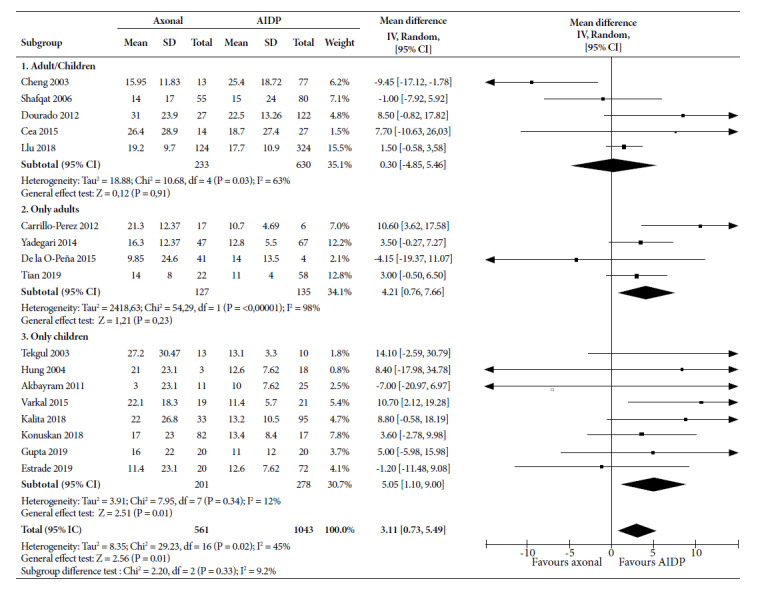
Forest Plot for time to hospitalization of axonal variants compared to demyelinating variant in patients with Guillain-Barré syndrome.

CI: confidence interval; AIDP: acute inflammatory demyelinating demyelinating polyradiculoneuropathy; SD: standard deviation.

For hospitalization time (Figure 3), MD was 0.30 days (95% CI: -4.85 to 5.46) for axonal variants in studies combining children and adults, not significant; while in adult-only studies it was 4.21 days longer (95% CI: 0.76 to 7.66), and in children only it was 5.05 days (95% CI: 1.10 to 9.00); the latter being significant. The combination of 17 studies showed a MD of 3.11 days (95% CI: 0.73 to 5.49) in axonal variants, not significant, and with a medium statistical heterogeneity (I^2^=45%).

**Figure 4:**
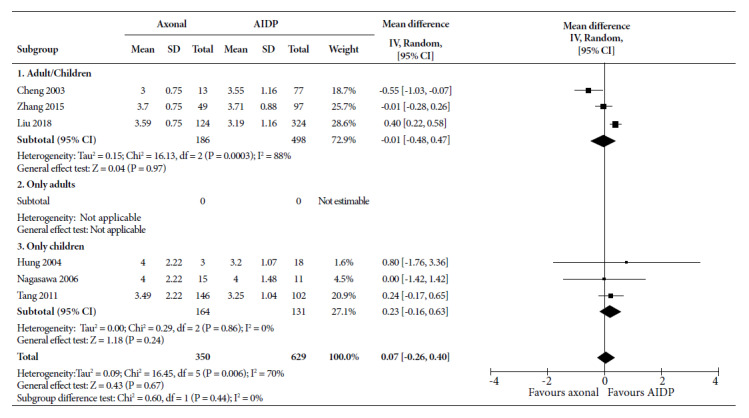
Forest Plot for disability dispersion measures (Hughes scale) of axonal variants compared to the demyelinating variant in patients with Guillain-Barré syndrome.

CI: confidence interval; AIDP: acute inflammatory demyelinating demyelinating polyradiculoneuropathy; SD: standard deviation

The Hughes disability score (Figure 4), showed an MD of -0.01 points (95% CI: -0.48 to 0.47) for axonal variants in studies combining children and adults, and it was 0.23 points in studies with children (95% CI: -0.16 to 0.63); both were not significant. The combination of six studies showed 0.07 (95% CI: -0.26 to 0.40) points more in axonal variants, which was not significant, with an average statistical heterogeneity (I^2^=70%).

**Figure 5:**
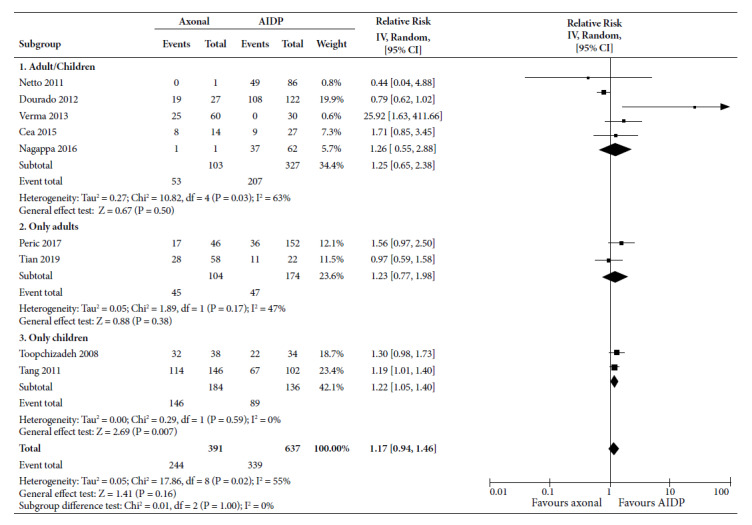
Forest Plot for disability (dichotomous variable) of axonal variants compared to demyelinating variant in patients with Guillain-Barré syndrome.

CI: confidence interval; AIDP: acute inflammatory demyelinating demyelinating polyradiculoneuropathy; SD: standard deviation.

When analyzing disability as a dichotomous variable (Figure 5), the studies that combined children and adults, the axonal subtypes had a probability of 1.25 (95% CI 0.65 to 2.38) times of developing disability; while the RR was 1.23 in adults (95% CI 0.77 to 1.98), which was not significant. RR was significant at 1.22 (95% CI 1.05 to 1.40) in studies on children. Overall, nine studies showed that axonal variants had an RR of 1.17 (95% CI 0.94 to 1.46), with average statistical heterogeneity (I^2^=55%).

**Figure 6:**
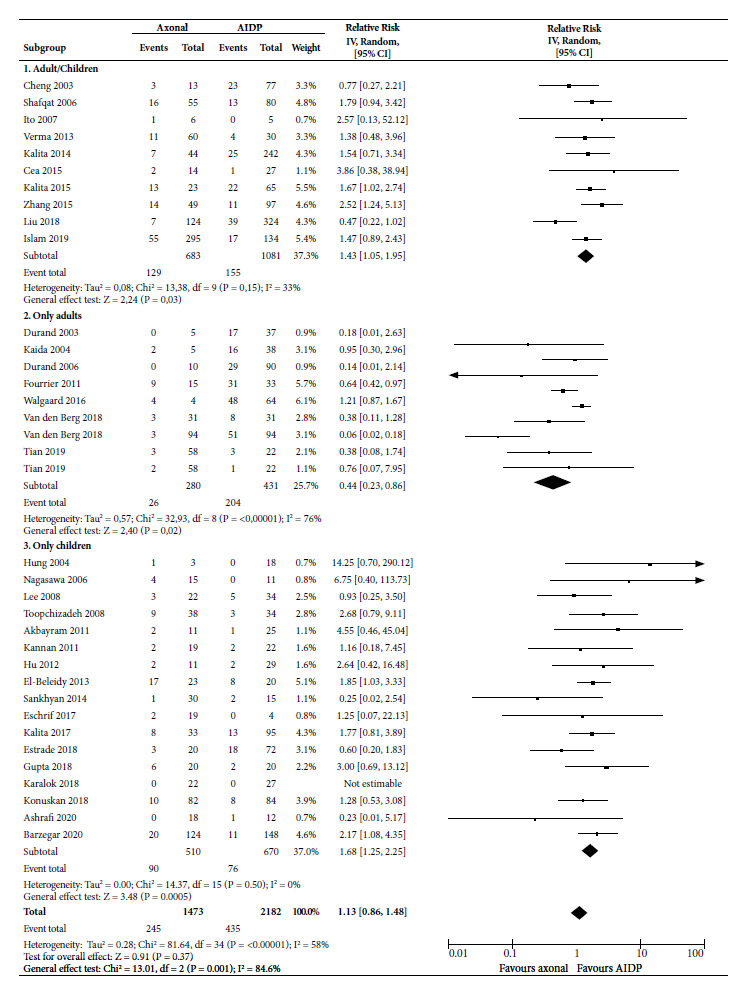
Forest Plot for mechanical ventilation requirement of the axonal variants compared to the demyelinating variant in patients with Guillain-Barré syndrome.

For MV requirement (Figure 6), axonal forms had a 1.43 (95% CI: 1.05 to 1.95) times probability of requiring MV in studies that combined children and adults, and it was 1.68 times in studies on children (95% CI: 1.25 to 2.25), both being significant. The association was inverse in adult-only studies, with an RR of 0.44 (95% CI: 0.23 to 0.86), also significant.

The 36 combined studies showed that axonal variants had an RR of 1.13 (95% CI 0.86 to 1.48) with a mean statistical heterogeneity (I^2^ = 58%), not being significant.

**Figure 7:**
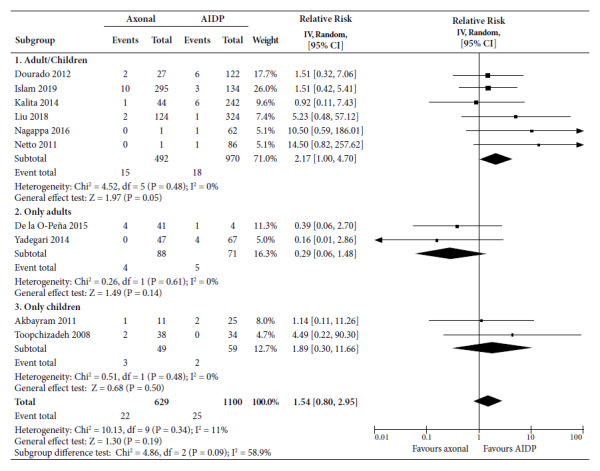
Forest Plot for deaths due to axonal variants compared to demyelinating variant in patients with Guillain-Barré syndrome.

When death was analyzed (Figure 7), axonal variants had a probability of death of 2.17 (95% CI 1.00 to 4.70) in the studies that combined children and adults which was marginally significant. The RR was 0.29 in studies on only adults (95% CI 0.06 to 1.48), and in studies on children only it was 1.89 (95% CI 0.30 to 11.66), both not significant. The 10 studies combined showed that axonal subtypes had a RR of death of 1.54 (95% CI 0.80 to 2.95), which was not significant, with a mean statistical heterogeneity (I^2^=58.9%).

Meta-analyses and Forest Plots were repeated for outcomes where significant differences were found in total or in any subgroup, but giving a 0% weight to studies that scored <7 on NOS.

For time in MV, MD for children (10.20; 95% CI: -0.49 to 20.89) and overall (5.57; 95% CI: -2.58 to 13.71) lost statistical significance when the CI increased; however, the directionality toward a higher number of days was maintained with axonal variants. For hospitalization time, MD remained significant for adults (3.03; 95% CI: 0.50 to 5.56), but decreased for children (3.84; 95% CI: -0.12 to 7.80) as total (2.05; 95% CI: -0.11 to 4.22); however, directionality toward a longer hospital stay was maintained with axonal variants.

The RR remained unchanged for the dichotomous outcome of disability and was significant for children (1.22; 95% CI 1.05 to 1.40), but not significant overall (1.17; 95% CI 0.94 to 1.46). The association in children remained significant for MV requirement (0.61; 95% CI 1.17 to 2.34), and the analysis with all articles became significant (1.39; 95% CI 1.07 to 1.80). There was no change in the significance or directionality of the associations for death (1.22; 95% CI 0.61 to 2.42).

## DISCUSSION

To our knowledge, this is the first systematic review that assesses differences of clinical outcomes according to the type of GBS. Axonal variants had 7.42 more days in MV (95% CI 0.36 to 1.48), and longer hospitalization time with 3.11 days (95% CI 0.73 to 5.49). There was a 17% higher occurrence of disability (RR 1.17, 95% CI 0.94 to 1.46), although not significant. There were no statistically significant differences for the other outcomes. In some cases, differences in directionality were found between studies that included only children, only adults, and both groups.

In terms of MV time, axonal variants require longer use compared to demyelinating ones. However, two articles report that the demyelinating subtype cases remain in MV for more days compared to axonal. The first study, Gupta 2019 ^20^, included only children and the MV time of the axonal subtype was 30 days, while for the demyelinating variant was 32 days. The second, Shafqat 2006 ^21^, included children and adults and reported 19 and 27 days in MV for the axonal and demyelinating variants, respectively. The longer MV time in the axonal subtype may be due to the fact that it presents more disabilities, increasing the possibility of respiratory muscle involvement. When analyzed by subgroups, children had a higher MV requirement compared to adults. The axonal variant in children is more severe and therefore requires greater use of MV.

Axonal variants required a longer ICU stay compared to demyelinating ones, although these differences were not significant. Several studies reported similar results, but some showed the opposite effect, such as the one by Estrade in 2019 ^22^. This study in children reported an average ICU stay of 3 days for the axonal subtype and 18 days for the demyelinating one. According to our results, the time in the ICU is longer in the axonal variant compared to the MV time. This discrepancy may be due to the fact that patients may be admitted to the ICU for reasons other than MV ^23^, but these do not specify the reason for ICU admission.

Hospitalization time statistically increased for the axonal variant. However, some articles report discrepancies. Cheng in 2003 ^24^, evaluating patients of all ages, found an average of 15.9 days of hospitalization for the axonal type and 25.4 days for the demyelinating type. Shafqat, in 2006 ^21^, reported 14 days of hospitalization for axonal compared to 15 days for demyelinating. The study by Peña in 2015 ^25^ was the only one that differed from studies in adults, where the axonal subtype was hospitalized for 9.8 days and the demyelinating for 14 days. Only 2 articles conducted only in children report differences. First, Akbayram in 2011 ^26^ found that the axonal variant had a mean hospitalization time of three days and the demyelinating was ten days, and Estrade, in 2019 ^22^, reported 11.4 days of hospitalization for axonal compared to 12.6 days for demyelinating. This difference may be due to the delay in diagnosis of GBS, leading to disease progression and severity of each variant, with axonal requiring the longest hospitalization time due to more complications.

Hospitalization time is shorter for the axonal variant compared to ICU stay and MV. This could be due to the fact that not all patients are admitted to the ICU. Therefore, if the hospitalization time in patients who are not admitted to the ICU is shorter for axonal variants, the combined effect on prolongation would be lower. Unfortunately, studies do not report hospitalization times stratified according to ICU admission or not. Another possibility is that after leaving the ICU, patients are transferred directly to specialized institutes, reducing hospitalization time.

A differentiated effect was observed between children and adults regarding the requirement of MV. The studies that included children and adults found 43% more MV use; while in children only 68% more use was found, both statistically significant. In contrast, research conducted only in adults showed 53% less MV use, also significant. When combining these values, the result shows no effect. We can conclude that the results regarding MV requirement are different according to age. Although in children, axonal variants increase ventilatory dysfunction, in adults, demyelinating variants have a higher risk of MV. This could be because children often have the axonal variant, which progresses rapidly, compared to adults, thus developing respiratory dysfunction and the need for MV. However, no studies were found comparing the requirement for MV between children and adults.

Disability was assessed according to the Hughes scale, as a continuous and dichotomous variable, although with different cut-off points. When assessed as a continuous variable, no differences were found. On the other hand, there was a tendency towards a greater occurrence of disability in the axonal forms when it was assessed as a dichotomized variable. All studies agree on this, except Netto in 2011 ^27^ and Dourado in 2012 ^28^, which reported more disability in AIDP. Although the weighted value showed more disability in axonal forms, this was not significant. The discrepancy between both forms of measurement may be due to the fact that, although the scores are similar on average, more patients with the axonal variant had values higher than the cut-off point. This could be interpreted that, although axonal forms cause more disability; when these occur in AIDP, they tend to cause higher scores, i.e., they are more severe. A study comparing the scores of patients already classified as disabled might show us this more clearly.

Lethality was higher in axonal variants in studies performed only in children, or in those that combined children and adults; whereas there were more deaths in AIDP when the studies were performed only in adults. When combining the different subgroups, the effects cancel each other out, resulting in a non-significant association. We are dealing with a case of interaction, in which age changes the directionality of the association. Due to the small number of studies per subgroup and the large CI, we cannot reach a clear conclusion, but it appears that axonal variants increase deaths in children, whereas AIDP has higher lethality in adults. Children with axonal variants have a more severe disease and are at greater risk of respiratory involvement and hemodynamic alteration, for which hospitalization or ICU stay is recommended ^29^^,^^30^. The contradiction is evident, since literature describes that children have fewer sequelae, better prognosis and lower mortality than adults ^31^. It is believed that the differences between GBS variants in children and adults are due to their history and risk factors, which affect severity and prognosis ^29^.

Selection bias can occur if studies with certain results have been systematically excluded, but the excluded studies were not available or did not contain the required information, and therefore could not have been excluded based on the results they report. Selection bias could also occur if one definition of GBS is favored over another. To avoid this, almost all available diagnostic criteria were used. Although this generates more methodological heterogeneity between studies, the benefit outweighs the risk. Not all studies included electromyographic studies to define the subtype of GBS, in which case the exposure and comparator could not be correctly classified. All studies were included even if they did not mention whether they performed electromyography. By not excluding these, we ensured that studies conducted in poor health systems were included in the review.

We excluded clinical trials, systematic reviews, narrative reviews and clinical practice guidelines because the results could be modified due to the intervention. Our literature search did not use the EMBASE database, due to not having free access to it. We do not anticipate this to cause selection bias, as six other databases and a large number of publications were included.

The evaluation of the publications and data extraction were not performed by GBS experts or methodological experts, which could lead to limitations. To minimize this, definitions and classifications of GBS were consulted with a methodological expert. One third of the studies had potential quality problems, but a sensitivity analysis was performed and we only included studies with high methodological quality. High statistical and/or methodological heterogeneity was found for some of the results, which was reported.

Stratifying the studies by age allowed us to evaluate the outcome of each variant by subgroups and to find significant interactions by age in two of the outcomes.

Our results can be used for the implementation of new public health policies, allowing health personnel to have a better knowledge of the prognoses of each variant and the resources needed to face future outbreaks. Early diagnosis and appropriate treatment can be carried out to reduce hospitalization time, MV requirement, disability and death. Finally, we suggest studying these results categorized by age group, as our review detected an important interaction of age in the effect that variants have on clinical outcomes.

In conclusion, axonal variants had a longer time in MV with a significant difference of 7.4 days more and a longer hospitalization time with 3.1 days more. Time in ICU was longer, but without significant difference. Disability was 17% higher in axonal variants, without being significant. There were no statistically significant differences when evaluated as a numerical variable.

The MV requirement among the axonal variants was not significant when analyzed as a whole. However, by subgroups, more MV was found for axonal variants in children; whereas, there was more MV in adults with the demyelinating variant. A similar trend was found for death without being significant.
