# Supplementary material for: Mechanical ventilation, hospitalization time, deaths and disability according to the variants of Guillain-Barré syndrome: systematic review and meta-analysis
Source: Rev Peru Med Exp Salud Publica. 2024 Aug 28;41(3):247–58. doi: 10.17843/rpmesp.2024.413.13509 (PMC11495932; doi:10.17843/rpmesp.2024.413.13509)
Supplement: Supplementary material. — Available in the electronic version of the RPMESP. [file rpmesp-41-03-13509-s001.docx]

**SUPPLEMENTAL MATERIAL**

**REFERENCES**

1. Organización Mundial de la Salud. Síndrome de Guillain–Barré [Internet]. WHO. 2016 [citado 17 de abril de 2020]. Disponible en: [https://www.who.int/es/news-room/fact-sheets/detail/guillain-barré-syndrome](https://www.who.int/es/news-room/fact-sheets/detail/guillain-barr%C3%A9-syndrome)
2. Torres M, Patrón Sánchez A, Bravo Perez R. Síndrome de Guillain Barré. Revista Cubana de Medicina Militar. 2003; 32(2):137-42.
3. Walling AD. Guillain-Barré Syndrome. American Family Physician. 2013; 87(3):191-7.
4. Instituto Nacional de Ciencias Neurológicas. Guía de Práctica Clínica para el Diagnóstico y Tratamiento del paciente con Síndrome de Guillain Barre [Internet]. 2018 [citado 17 de abril de 2020]. Disponible en: <http://www.dge.gob.pe/portal/docs/tools/sg/RD2012018Guia_Guillain_Barre.pdf>
5. Manorenj S, Muralikrishna PS, Sravan KS, Sagari N. Acute sensory axonal neuropathy (ASAN): An unusual pure sensory variant of Guillain-Barre Syndrome associated with IgM autoantibodies against GM1. International Journal of Scientific Research. 2017; 6(9):698-99.
6. van den Berg B, Walgaard C, Drenthen J, Fokke C, Jacobs BC, van Doorn PA. Guillain-Barré syndrome: pathogenesis, diagnosis, treatment and prognosis. Nature Reviews Neurology. 2014; 10(8):469-82.
7. Rebolledo-García D, González-Vargas P, Salgado-Calderón I. Síndrome de Guillain-Barré: viejos y nuevos conceptos. Medicina Interna de México. 2018; 34(1):72-81.
8. Walling AD, Dickson G. Guillain-Barré syndrome. Am Fam Physician. 2013; 87(3):191-7.
9. Karalok ZS, Taskin BD, Yanginlar ZB, Gurkas E, Guven A, Degerliyurt A, et al. Guillain-Barré syndrome in children: subtypes and outcome. Child's Nervous System. 2018; 34(11):2291-7.
10. Vucic S, Kiernan MC, Cornblath DR. Guillain-Barré syndrome: an update. J Clin Neurosci. 2009; 16(6):733-41.
11. Loayza-Alarico MJ, De la Cruz-Vargas JA, Alatrista MDS. Guillain-Barré syndrome, epidemic outbreak in Peru in June 2019. Salud Pública de México. 2019; 61(5):556-557.
12. Munayco CV, Soto Cabezas MG, Reyes MF, Arica Gutiérrez JA, Napanga Saldaña O. Epidemiology of guillain-barré syndrome in Perú. Revista Peruana de Medicina Experimental y Salud Pública. 2019; 36(1):10-16.
13. Loayza-Alarico MJ, De la Cruz-Vargas JA, Alatrista MDS. Guillain-Barré syndrome, epidemic outbreak in Peru in June 2019. Salud Pública de México. 2019; 61(5):556-557.
14. Hernández-Torruco J, Canul-Reich J, Frausto-Solís J, Méndez-Castillo J. Predictores de falla respiratoria y de la necesidad de ventilación mecánica en el síndrome de Guillain-Barré: una revisión de la literatura. Revista Mexicana de Neurociencias. 2013; 14(5):272-80.
15. van den Berg B, Storm EF, Garssen MJP, Blomkwist-Markens PH, Jacobs BC. Clinical outcome of Guillain-Barré syndrome after prolonged mechanical ventilation. Journal of Neurology, Neurosurgery and Psychiatry [Internet]. 2018; 89(9):949-954.
16. Wang Y, Zhang H-L, Wu X, Zhu J. Complications of Guillain-Barré syndrome. Expert Review of Clinical Immunology. 2016; 12(4):439-48.
17. de Boisanger L. Outcomes for patients with Guillain-Barré syndrome requiring mechanical ventilation: a literature review. Irish Journal of Medical Science. 2016; 185(1):11-15.
18. Netto AB, Taly AB, Kulkarni GB, Rao UGS, Rao S. Mortality in mechanically ventilated patients of Guillain Barré Syndrome. Annals of Indian Academy of Neurology. 2011; 14(4):262-66.
19. Tekgul H, Serdaroglu G, Tutuncuoglu S. Outcome of axonal and demyelinating forms of Guillain-Barré syndrome in children. Pediatric Neurology. 2003; 28(4):295-99.
20. Konuşkan B, Okuyaz Ç, Taşdelen B, Kurul SH, Anlar B. Electrophysiological Subtypes and Prognostic Factors of Childhood Guillain-Barré Syndrome. Noro Psikiyatr Ars. 2018; 55(3):199-204.
21. Ansari B, Basiri K, Derakhshan Y, Kadkhodaei F, Okhovat AA. Epidemiology and Clinical Features of Guillain-Barre Syndrome in Isfahan, Iran. Advanced Biomedical Research. 2018; 7:87.
22. Moher D, Liberati A, Tetzlaff J, Altman DG. Preferred reporting items for systematic reviews and meta-analyses: the PRISMA statement. BMJ. 2009; 339: b2535.
23. Higgins J, Thomas J, Chandler J, Cumpston M, Li T, Page M, et al. Cochrane Handbook for Systematic Reviews of Interventions version 6.2 [Internet]. The Cochrane Collaboration; 2021 [Citado el 30 de mayo de 2021]. Disponible en: https://training.cochrane.org/handbook
24. Wells G, Shea B, O´Connell D, Peterson J, Welch V, Losos M, et al. The Newcastle-Ottawa Scale (NOS) for assessing the quality of nonrandomised studies in meta-analyses. Ottawa Hospital Research Institute [Internet]. 2019 [citado 11 de junio de 2020]. Disponible en:<http://www.ohri.ca/programs/clinical_epidemiology/oxford.asp>
25. Ma J, Liu W, Hunter A, Zhang W. Performing meta-analysis with incomplete statistical information in clinical trials. BMC Medical Research Methodology. 2008; 18(8):56.
26. Yadegari S, Kazemi N, Nafissi S. Clinical and electrophysiological features of Guillain-Barré syndrome in Iran. Journal of Clinical Neuroscience. 2014; 21(9):1554-7.
27. Sankhyan N, Sharma S, Konanki R, Gulati S. Childhood Guillain–Barré syndrome subtypes in northern India. Journal of Clinical Neuroscience. 2014; 21(3):427-30.
28. Rosen BA. Guillain-Barre Syndrome. Pediatrics in Review. 2012; 33(4):164-71.
29. Ortez González CI, Conradi ÁD. Síndrome de Guillain-Barré en la infancia. Anales de Pediatría Continuada. 2013; 11(2):98-103.
30. Sankhyan N, Sharma S, Konanki R, Gulati S. Childhood Guillain–Barré syndrome subtypes in northern India. Journal of Clinical Neuroscience. 2014; 21(3):427-30.
31. Organización Mundial de la Salud. Identificación y tratamiento del síndrome de Guillain-Barré en el contexto del brote del virus Zika [Internet]. WHO. 2016 [citado 15 de junio de 2020]. Disponible en:<https://apps.who.int/iris/bitstream/handle/10665/204587/WHO_ZIKV_MOC_16.4_spa.pdf;jsessionid=5E2343E3448A0DA3A82E923FFA1D417E?sequence=1>
32. Asbury AK, Cornblath DR. Assessment of current diagnostic criteria for Guillain‐Barré syndrome. Annals of neurology. 1990; 27(1S), S21-S24.
33. Leonhard SE, Mandarakas MR, Gondim FAA, Bateman K, Ferreira MLB, Cornblath DR, et al. Diagnosis and management of Guillain–Barré syndrome in ten steps. Nature Reviews Neurology. 2019; 15(11):671-83.
34. Yoon B-A, Bae JS, Kim JK. Electrognostic findings of Guillain-Barré syndrome. ACN. 2020; 22(1):13-18.
35. Ministerio de sanidad, servicios sociales e igualdad. Clasificación Internacional de Enfermedades - 10.ª Revisión Modificación Clínica. [Internet]. España: Boletín Oficial del Estado; 2018 [citado de 09 mayo de 2020]. Disponible en: <https://www.mscbs.gob.es/estadEstudios/estadisticas/normalizacion/CIE10/CIE10ES_2018_diag_pdf_20180202.pdf>
36. Rangan R, Tullu M, Deshmukh C, Mondkar S, Agrawal M. Clinical Profile and Outcome of Guillain–Barre Syndrome in Pediatric Patients Admitted to a Tertiary Care Centre: A Retrospective Study. Neurology India. 2021; 69(1):81.
37. Ruiz-Sandoval J, Salvatella-Gutiérrez A, López-Valencia G, Chiquete E, Ruiz-Herrera V, Pérez-Gómez H, et al. Clinical Characteristics and Predictors of Short-Term Outcome in Mexican Adult Patients with Guillain-Barré Syndrome. Neurology India. 2021; 69(1):107.
38. Cadenas Yaurimo SE, Castro Ortega ML. Factores asociados a mal pronóstico del Síndrome de Guillain-Barré en dos hospitales de Huancayo en el brote del año 2019 [Tesis]. Huancayo: Universidad Nacional de Centro del Perú; 2020. 67 p.
39. Coya C, Catherine Y. Características epidemiológicas y clínicas del Síndrome de Guillain Barré en pacientes diagnosticados en el Hospital Honorio Delgado Espinoza [Tesis]. Arequipa: Universidad Nacional de San Agustín de Arequipa; 2020. 48 p.
40. Velasquéz VA, Vásquez AE. Características clínico-epidemiológicas asociadas con resultados al alta hospitalaria en pacientes con síndrome de Guillain-Barré en el Hospital Nacional Dos de Mayo, 2011-2015, Lima-Perú [Tesis]. Lima: Universidad Nacional Mayor de San Marcos; 2020. 33 p.
41. Bravo JCF. Morbimortalidad del Síndrome de Guillain-Barré en el Instituto Nacional de Salud del Niño 2011- 2015 [Tesis]. Lima: Universidad San Martin de Porres; 2019. 92 p.
42. Cabrera-Ortiz A, Álvarez-Aguilar P, Porras-Vargas J. Caracterización clínica del síndrome de Guillain-Barré en el Hospital México, Costa Rica. Revista Hispanoamericana de Ciencias de la Salud. 2019; 5(2):55-62.
43. [Kili](https://www.scopus.com/authid/detail.uri?authorId=56592297600&eid=2-s2.0-85075463897)ç [B](https://www.scopus.com/authid/detail.uri?authorId=56592297600&eid=2-s2.0-85075463897), [Gungor S](https://www.scopus.com/authid/detail.uri?authorId=7003442366&eid=2-s2.0-85075463897), [Ozgor B.](https://www.scopus.com/authid/detail.uri?authorId=35761477000&eid=2-s2.0-85075463897) Clinical, electrophysiological findings and evaluation of prognosis of patients with Guillain-barré syndrome. [Turkish Journal of Pediatrics](https://www.scopus.com/sourceid/16222). 2019; 61(2): 200-208.
44. Sedano MJ, Orizaola P, Gallardo E, García A, Pelayo-Negro AL, Sánchez-Juan P, et al. A unicenter, prospective study of Guillain-Barré syndrome in Spain. Acta Neurologica Scandinavica. 2019; 139(6):546-54.
45. Vera-Carrasco O. Síndrome de Guillain Barré. Cuadernos del Hospital de Clínicas. 2019; 60(2):59-64.
46. [Yadav S](https://www.scopus.com/authid/detail.uri?authorId=57210376731&eid=2-s2.0-85070524407), [Jain P](https://www.scopus.com/authid/detail.uri?authorId=55257167900&eid=2-s2.0-85070524407), [Sharma S](https://www.scopus.com/authid/detail.uri?authorId=57221072439&eid=2-s2.0-85070524407), [Kumar V](https://www.scopus.com/authid/detail.uri?authorId=57221614776&eid=2-s2.0-85070524407), [Aneja S](https://www.scopus.com/authid/detail.uri?authorId=57207562102&eid=2-s2.0-85070524407). Guillain-Barre syndrome in North Indian children: Clinical and serial electrophysiological features. [Neurology India](https://www.scopus.com/sourceid/17969). 2019; 67(3): 724-727.
47. Yosha-Orpaz N, Aharoni S, Rabie M, Nevo Y. Atypical Clinical Presentations of Pediatric Acute Immune-Mediated Polyneuropathy. Journal of Child Neurology. 2019; 34(5):268-76.
48. [Zhou RM](https://www.scopus.com/authid/detail.uri?authorId=57215113322&eid=2-s2.0-85075537172), [Shao B](https://www.scopus.com/authid/detail.uri?authorId=7103207792&eid=2-s2.0-85075537172), [Luo C](https://www.scopus.com/authid/detail.uri?authorId=57189058186&eid=2-s2.0-85075537172), [Dai HY](https://www.scopus.com/authid/detail.uri?authorId=57205028148&eid=2-s2.0-85075537172), [Xu J](https://www.scopus.com/authid/detail.uri?authorId=56809704100&eid=2-s2.0-85075537172), [Li XY](https://www.scopus.com/authid/detail.uri?authorId=57208293918&eid=2-s2.0-85075537172), [Wang N](https://www.scopus.com/authid/detail.uri?authorId=57220879811&eid=2-s2.0-85075537172), [Zhang RX](https://www.scopus.com/authid/detail.uri?authorId=8532145900&eid=2-s2.0-85075537172), [Ji F](https://www.scopus.com/authid/detail.uri?authorId=57203929771&eid=2-s2.0-85075537172), [Yang B](https://www.scopus.com/authid/detail.uri?authorId=57215339533&eid=2-s2.0-85075537172), [Jiang ZW](https://www.scopus.com/authid/detail.uri?authorId=57205028406&eid=2-s2.0-85075537172), [Hu F](https://www.scopus.com/authid/detail.uri?authorId=57215652918&eid=2-s2.0-85075537172). Analysis of differences in epidemiology and clinical features of Guillain-Barré syndrome between rural and urban areas of southern China. [Zhonghua yi xue za zhi](https://www.scopus.com/sourceid/50191). 2019; 99(43): 3432-36.
49. Aragonès JM, Altimiras J, Alonso F, Celedón G, Alfonso S, Roura P, et al. Incidencia y características clínicas del síndrome de Guillain-Barré en la comarca de Osona (Barcelona, España) (2003-2016). Neurología. 2018; 6.
50. Hafsteinsdóttir B, Ólafsson E, Jakobsson F. Incidence and outcome of Guillain-Barré syndrome in Iceland: A population-based study. Acta Neurologica Scandinavica. 2018; 138(5):454-8.
51. Vázquez R, Analiz L. Características clínicas y epidemiológicas del síndrome de guillain-barré en el Hospital Central del Instituto de Previsión Social, 2016-2017 [Tesis]. Oviedo: Universidad Nacional de Caaguazú; 2018. 70 p.
52. Ballón-Manrique B, Campos-Ramos N. Características clínicas y paraclínicas del Síndrome de Guillain-Barré en el Hospital Regional Lambayeque. Revista de Neuro-Psiquiatría. 2017; 80(1):22-6.
53. Chareyre J, Hully M, Simonnet H, Musset L, Barnerias C, Kossorotoff M, et al. Acute axonal neuropathy subtype of Guillain Barré syndrome in a French pediatric series: Adequate follow-up may require repetitive electrophysiological studies. European Journal of Paediatric Neurology. 2017; 21(6):891-7.
54. Cabrera Ortiz A. Análisis clínico y epidemiológico del Síndrome Guillain Barré en pacientes internados en el hospital México durante el periodo del II semestre 2011 al 1 semestre de 2015 [Tesis]. Costa Rica: Universidad de Costa Rica; 2016.
55. González P, García X, Guerra A, Arango JC, Delgado H, Uribe CS, et al. Experience with Guillain-Barré syndrome in a neurological Intensive Care Unit. Neurologia. 2016; 31(6):389-94.
56. [Salehiomran MR](https://www.scopus.com/authid/detail.uri?authorId=57210257410&eid=2-s2.0-84961595225), [Nikkhah A](https://www.scopus.com/authid/detail.uri?authorId=36893948800&eid=2-s2.0-84961595225), [Mahdavi M](https://www.scopus.com/authid/detail.uri?authorId=57188557469&eid=2-s2.0-84961595225). Prognosis of Guillain-Barré syndrome in children. [Iranian Journal of Child Neurology](https://www.scopus.com/sourceid/19700174989). 2016; 10(2): 38-41.
57. Wu X, Shen D, Li T, Zhang B, Li C, Mao M, et al. Distinct Clinical Characteristics of Pediatric Guillain-Barré Syndrome: A Comparative Study between Children and Adults in Northeast China. PloS One. 2016; 11(3):12.
58. [Zhang](https://pubmed.ncbi.nlm.nih.gov/?term=Zhang+J&cauthor_id=27470955) [J](https://pubmed.ncbi.nlm.nih.gov/27470955/#affiliation-1), [Song](https://pubmed.ncbi.nlm.nih.gov/?term=Song+XJ&cauthor_id=27470955) XJ, [Hou](https://pubmed.ncbi.nlm.nih.gov/?term=Hou+HQ&cauthor_id=27470955) HQ, [Tan](https://pubmed.ncbi.nlm.nih.gov/?term=Tan+GJ&cauthor_id=27470955) GJ, [Bian](https://pubmed.ncbi.nlm.nih.gov/?term=Bian+GY&cauthor_id=27470955) GY, [Li](https://pubmed.ncbi.nlm.nih.gov/?term=Li+B&cauthor_id=27470955) B, [Chen](https://pubmed.ncbi.nlm.nih.gov/?term=Chen+LP&cauthor_id=27470955) LP, [Wang](https://pubmed.ncbi.nlm.nih.gov/?term=Wang+L&cauthor_id=27470955) L, [Guo](https://pubmed.ncbi.nlm.nih.gov/?term=Guo+L&cauthor_id=27470955) L. Electrophysiological subtypes and long term prognosis of Guillain-Barré síndrome. Yiigle. 2016; 96(25):1987-90.
59. [Pasanen ME](https://www.scopus.com/authid/detail.uri?authorId=8504911500&eid=2-s2.0-84924989461). Guillain-Barré syndrome. [Hospital Medicine Clinics](https://www.scopus.com/sourceid/21100197950). 2015; 4(2): 177-1901.
60. Ramos B, Geraldine K. Grados de fuerza muscular y su relación con los subtipos del síndrome de guillain barré en los pacientes afectados entre los años 2009 al 2013 [Tesis]. Lima: Universidad Mayor de San Marcos; 2015. 95 p.
61. Bhagat H, Dash H, Chauhan R, Khanna P, Bithal P. Intensive care management of Guillain-Barre syndrome: A retrospective outcome study and review of literature. Journal of Neuroanaesthesiology and Critical Care. 2014; 01(03):188-97.
62. González VD, Aguilera POR, Ruiz MFJ, Casamajor CMA, Parrón CI. Síndrome de Guillain-Barré en la infancia. MEDISAN. 2014; 18(5):613-20.
63. Montes de Oca SL, Albert Victorero A. Síndrome de Guillain-Barré. Revista de Ciencias Médicas de Pinar del Río. 2014; 18(2):275-83.
64. Pandian GD. Analysis of Clinical and Investigatory Profile in the management and Outcome of Guillain – Barre Syndrome [Masters]. Chennai: Madras Medical College; 2014. 91 p.
65. Ye Y-Q, Wang K-R, Sun L, Wang Z. Clinical and electrophysiologic features of childhood Guillain-Barré syndrome in Northeast China. Journal of the Formosan Medical Association. 2014; 113(9):634-9.
66. [Gazioglu S](https://www.scopus.com/authid/detail.uri?authorId=21740701100&eid=2-s2.0-84875671623), [Tomak T](https://www.scopus.com/authid/detail.uri?authorId=55636557600&eid=2-s2.0-84875671623), [Boz C.](https://www.scopus.com/authid/detail.uri?authorId=6701728553&eid=2-s2.0-84875671623) Clinical characteristics and prognosis of guillain barre síndrome. Journal of Neurological Sciences. 2013; 30(1):124-134.
67. González-Suárez I, Sanz-Gallego I, Rodríguez de Rivera FJ, Arpa J. Guillain-Barré syndrome: natural history and prognostic factors: a retrospective review of 106 cases. BMC Neurology. 2013; 13:95.
68. Uribarri López M, Aguilera Celorrio L, Miguel Martínez C, Yuste del Valle S. El síndrome de Guillain Barré en la unidad de cuidados críticos en los últimos 10 años. Revista Española de Anestesiología y Reanimación. 2013; 60(5):257-62.
69. Witsch J, Galldiks N, Bender A, Kollmar R, Bösel J, Hobohm C, et al. Long-term outcome in patients with Guillain-Barré syndrome requiring mechanical ventilation. Journal of Neurology. 2013; 260(5):1367-74.
70. Lin J-J, Hsia S-H, Wang H-S, Lyu R-K, Chou M-L, Hung P-C, et al. Clinical variants of Guillain-Barré syndrome in children. Pediatric Neurology. 2012; 47(2):91-6.
71. Paul BS, Bhatia R, Prasad K, Padma MV, Tripathi M, Singh MB. Clinical predictors of mechanical ventilation in Guillain-Barré syndrome. Neurology India [Internet]. 2012 [citado 23 de mayo de 2021]; 60(2):150-3. Disponible en: https://www.neurologyindia.com/article.asp?issn=0028-3886;year=2012;volume=60;issue=2;spage=150;epage=153;aulast=Paul
72. [Qiu Y-Q](https://www.scopus.com/authid/detail.uri?authorId=37049474800&eid=2-s2.0-84875425770), [Bi X-Y](https://www.scopus.com/authid/detail.uri?authorId=12807910900&eid=2-s2.0-84875425770), [Cai J-M.](https://www.scopus.com/authid/detail.uri?authorId=24437664400&eid=2-s2.0-84875425770) Clinical features of variant guillain-barre syndrome. [Journal of Clinical Neurology (China)](https://www.scopus.com/sourceid/17600155040). 2012; 25(3): 230-23225.
73. Alexander M, Prabhakar AT, Aaron S, Thomas M, Mathew V, Patil AK. Utility of neurophysiological criteria in Guillain Barre syndrome: Subtype spectrum from a tertiary referral hospital in India. Neurology India. 2011; 59(5):722-6.
74. Incecik F, Hergüner MO, Altunbasak S. Guillain-Barré syndrome in children. Neurological Sciences. 2011;32(3):381-5.
75. [Areeyapinan](https://pubmed.ncbi.nlm.nih.gov/?term=Areeyapinan+P&cauthor_id=20973317) P, [Phanthumchinda](https://pubmed.ncbi.nlm.nih.gov/?term=Phanthumchinda+K&cauthor_id=20973317) K. Guillain-Barre syndrome: a clinical study in King Chulalongkorn Memorial Hospital. Journal of Medical Association of Thailand. 2010;93(10):1150-5.
76. Méndez CJJ, Ruano CLA, Moreno BM, López SL. Descripción estadística de la evaluación clínica y neurofisiológica del Síndrome de Guillain-Barré en México. Medicina Interna de México [Internet]. 2018 [citado el 23 de julio de 2020]. Disponible en: https://www.researchgate.net/profile/Mario_Ojeda/publication/292708863_Memoria_del_2_Encuentro_de_Biometria_y_la_V_Reunion_de_la_Region_Centroamericana_y_del_Caribe_de_la_Sociedad_de_Biometria/links/56b0ccb008ae9c1968b97050/Memoria-del-2-Encuentro-de-Biometria-y-la-V-Reunion-de-la-Region-Centroamericana-y-del-Caribe-de-la-Sociedad-de-Biometria.pdf#page=169
77. Pithadia AB, Kakadia N. Guillain-Barré syndrome (GBS). Pharmacological Reports. 2010; 62(2):220-232.
78. van der Linden V, da Paz JA, Casella EB, Marques-Dias MJ. Guillain-Barré syndrome in children: Clinic, laboratorial and epidemiologic study of 61 patients. Arquivos de Neuro-Psiquiatría. 2010; 68(1):12-7.
79. Ye Y, Zhu D, Wang K, Wu J, Feng J, Ma D, et al. Clinical and electrophysiological features of the 2007 Guillain-Barré syndrome epidemic in northeast China. Muscle Nerve [Internet]. 2010 [citado el 23 de mayo de 2021]; 42(3):311-4. Disponible en: https://onlinelibrary.wiley.com/doi/abs/10.1002/mus.21701
80. Aguilar CJ, Medina MT, Hernández R, Amador C, Matamoros M, Pinto JAL. Caracterización clínica y neurofisiológica del síndrome de Guillain-Barré en el Hospital Escuela. Rev Cienc Tecnol [Internet]. 2009 [citado el 23 de mayo de 2021]; 66-86. Disponible en: https://www.camjol.info/index.php/RCT/article/view/520/0
81. [Dididze MN.](https://www.scopus.com/authid/detail.uri?authorId=57214974174&eid=2-s2.0-66149136913) Clinical variants of Guillain-Barre syndrome: some aspects of differential diagnosis. [Georgian medical new](https://www.scopus.com/sourceid/3900148601)s. 2009; 166:48–51.
82. [Kollar K](https://www.scopus.com/authid/detail.uri?authorId=14070318000&eid=2-s2.0-76549103760), [Liptai Z](https://www.scopus.com/authid/detail.uri?authorId=8593385500&eid=2-s2.0-76549103760), [Rosdy B](https://www.scopus.com/authid/detail.uri?authorId=14070485300&eid=2-s2.0-76549103760), [Moser J](https://www.scopus.com/authid/detail.uri?authorId=7103187636&eid=2-s2.0-76549103760). Guillain-Barré syndrome in childhood. Ideggyogyaszati Szemle. 2009; 62(11-12):399–40430.
83. Ramírez [Z](https://dialnet.unirioja.es/servlet/autor?codigo=2922728)M, [Ganuza](https://dialnet.unirioja.es/servlet/autor?codigo=2922734) CRB, [Valle](https://dialnet.unirioja.es/servlet/autor?codigo=2922737) DAA, [Galán](https://dialnet.unirioja.es/servlet/autor?codigo=2922739) PEV, [González](https://dialnet.unirioja.es/servlet/autor?codigo=2922743) CIO. Síndrome de Guillain-Barré en edad pediátrica. Perfil epidemiológico, clínico y terapéutico en un hospital de El Salvador. [Revista de neurología](https://dialnet.unirioja.es/servlet/revista?codigo=2362) [Internet]. 2009 [citado el 07 de junio de 2021];[48(6)](https://dialnet.unirioja.es/ejemplar/277822):292-297. Disponible en:<https://dialnet.unirioja.es/servlet/articulo?codigo=3667112>
84. Caballero SM, Zelaya de Lobo R. Variante más frecuente de Guillain Barre y su correlación con la severidad y funcionalidad en pacientes menores de 18 años del Hospital Escuela. Revista Médica de los Posgrados de Medicina [Internet]. 2008 [citado el 23 de mayo de 2021]; 11(2):7. Disponible en: http://cidbimena.desastres.hn/RMP/pdf/2008/pdf/Vol11-2-2008-13.pdf
85. Dhar R, Stitt L, Hahn AF. The morbidity and outcome of patients with Guillain-Barré syndrome admitted to the intensive care unit. Journal of the Neurological Sciences. 2008; 264(1-2):121-8.
86. Moreno Ruiz I, Cabrera Linares AE, Curbelo Pérez R. Comportamiento del Síndrome de Guillain – Barré en las Unidades de Terapia de la provincia Ciego de Ávila. 16 de abril [Internet]. 2008 [citado 24 de julio de 2020]. Disponible en: http://www.16deabril.sld.cu/rev/233/02.html
87. Zazula R, Řezáč T, Cihlář J. Guillain-Barré syndrome: Specific features of intensive care, potential for therapeutic use of plasmapheresis, and our experience. Prakt Lek. [Internet]. 2008 [citado el 23 de mayo de 2021]; 88 (10): 582-8. Disponible en: https://www.prolekare.cz/casopisy/prakticky-lekar/2008-10/syndrom-guillain-barre-specifika-intenzivni-pece-moznosti-terapeutickeho-vyuziti-plazmaferezy-a-nase-zkusenosti-971
88. Markoula S, Giannopoulos S, Sarmas I, Tzavidi S, Kyritsis AP, Lagos G. Guillain-Barré syndrome in northwest Greece. Acta Neurologica Scandinavica. 2007; 115 (3): 167-73.
89. Nachamkin I, Barbosa PA, Ung H, Lobato C, Rivera AG, Rodriguez P, et al. Patterns of Guillain-Barré syndrome in children: Results from a Mexican population. Neurology. 2007; 69(17):1665-71.
90. Ortiz-Corredor F, Pena-Preciado M, Diaz-Ruiz J. Motor recovery after Guillain-Barre syndrome in childhood. Disability and Rehabilitation. 2007; 29(11-12):883-9.
91. Sinha S, Prasad KN, Jain D, Pandey CM, Jha S, Pradhan S. Preceding infections and anti-ganglioside antibodies in patients with Guillain-Barrésyndrome: A single centre prospective case-control study. Clinical Microbiology and Infection. 2007; 13(3):334-7.
92. Arami MA, Yazdchi M, Khandaghi R. Epidemiology and characteristics of Guillain-Barré syndrome in the northwest of Iran. Annals of Saudi Medicine. 2006;26(1):22-7.
93. [Dai Y-M](https://www.scopus.com/authid/detail.uri?authorId=57217626728&eid=2-s2.0-33747283133), [Wang D-S](https://www.scopus.com/authid/detail.uri?authorId=57219135713&eid=2-s2.0-33747283133), [Wang S-R](https://www.scopus.com/authid/detail.uri?authorId=57191712420&eid=2-s2.0-33747283133), [Hu X-L](https://www.scopus.com/authid/detail.uri?authorId=55496309200&eid=2-s2.0-33747283133), [Zhao Q-J](https://www.scopus.com/authid/detail.uri?authorId=55743326800&eid=2-s2.0-33747283133), [Zhang L-M.](https://www.scopus.com/authid/detail.uri?authorId=37065392100&eid=2-s2.0-33747283133) Epidemic characteristics and prognosis of patients with Guillain-Barre syndrome: Review of 254 cases in 5 years. Chinese Journal of Clinical Rehabilitation. 2006;10(24):18-2125.
94. França Jr. MC, Deus-Silva L, De Castro R, Garibaldi SG, Pfeilsticker BHM, Nucci A, et al. Guillain-Barré syndrome in the elderly: Clinical, electrophysiological, therapeutic and outcome features. Arquivos de Neuropsiquiatria. 2005;63(3B):772-5.
95. Ryan MM. Guillain-Barré syndrome in childhood. Journal of Paediatrics and Child Health. 2005; 41(5-6):237-41.
96. [Sundar](https://pubmed.ncbi.nlm.nih.gov/?term=Sundar+U&cauthor_id=16334619) U, [Abraham](https://pubmed.ncbi.nlm.nih.gov/?term=Abraham+E&cauthor_id=16334619) E, [Gharat](https://pubmed.ncbi.nlm.nih.gov/?term=Gharat+A&cauthor_id=16334619) A, [Yeolekar](https://pubmed.ncbi.nlm.nih.gov/?term=Yeolekar+ME&cauthor_id=16334619) ME, [Trivedi](https://pubmed.ncbi.nlm.nih.gov/?term=Trivedi+T&cauthor_id=16334619) T, [Dwivedi](https://pubmed.ncbi.nlm.nih.gov/?term=Dwivedi+N&cauthor_id=16334619) N. Neuromuscular respiratory failure in Guillain-Barre Syndrome: evaluation of clinical and electrodiagnostic predictors. Journal of the Association of Physicians of India. 2005; 53:764-8.
97. Chroni E, Papapetropoulos S, Gioldasis G, Ellul J, Diamadopoulos N, Papapetropoulos T. Guillain-Barré syndrome in Greece: seasonality and other clinico-epidemiological features. European Journal of Neurology. 2004; 11(6):383-8.
98. Hiraga A, Mori M, Ogawara K, Hattori T, Kuwabara S. Differences in patterns of progression in demyelinating and axonal Guillain-Barré syndromes. Neurology. 2003; 61(4):471-4.
99. [Nishimoto Y](https://www.scopus.com/authid/detail.uri?authorId=7102885786&eid=2-s2.0-0035751672), [Odaka M](https://www.scopus.com/authid/detail.uri?authorId=56262973400&eid=2-s2.0-0035751672), [Yuki N.](https://www.scopus.com/authid/detail.uri?authorId=55325339300&eid=2-s2.0-0035751672) Guillain-Barré syndrome (including Fisher syndrome). Nihon Rinsho. 2001; 59(8):529-39.
100. [Téllez-Zenteno JF](https://pesquisa.bvsalud.org/portal/?lang=pt&q=au:%22T%C3%A9llez%20Zenteno,%20Jos%C3%A9%20Francisco%22), [Jacinto-Tinajero JC](https://pesquisa.bvsalud.org/portal/?lang=pt&q=au:%22Jacinto%20Tinajero,%20Juan%20Carlos%22), [Avila-Fuentes A](https://pesquisa.bvsalud.org/portal/?lang=pt&q=au:%22Avila%20Fuentes,%20Alberto%22), [García-Ramos G](https://pesquisa.bvsalud.org/portal/?lang=pt&q=au:%22Garc%C3%ADa%20Ramos,%20Guillermo%22), [Negrete-Pulido O](https://pesquisa.bvsalud.org/portal/?lang=pt&q=au:%22Negrete%20Pulido,%20Oscar%22), [Sentíes-Madrid H](https://pesquisa.bvsalud.org/portal/?lang=pt&q=au:%22Sent%C3%ADes%20Madrid,%20Horacio%22). Síndrome de Guillain-Barré: experiencia en un hospital de tercer nivel. [Rev. invest. Clín [Internet]. 2001 [citado el 07 de junio de 2021];](http://portal.revistas.bvs.br/transf.php?xsl=xsl/titles.xsl&xml=http://catserver.bireme.br/cgi-bin/wxis1660.exe/?IsisScript=../cgi-bin/catrevistas/catrevistas.xis%7Cdatabase_name=TITLES%7Clist_type=title%7Ccat_name=ALL%7Cfrom=1%7Ccount=50&lang=pt&comefrom=home&home=false&task=show_magazines&request_made_adv_search=false&lang=pt&show_adv_search=false&help_file=/help_pt.htm&connector=ET&search_exp=Rev.%20invest.%20cl%C3%ADn) 53(4): 311-314. Disponible en:<https://pesquisa.bvsalud.org/portal/resource/pt/lil-314459>
101. Fletcher DD, Lawn ND, Wolter TD, Wijdicks EF. Long-term outcome in patients with Guillain-Barré syndrome requiring mechanical ventilation. Neurology. 2000; 54(12):2311-5.
102. Jiang W, Xu Y, Wu B. Electrophysiological classification of Guillain-Barre syndrome: Analysis of 100 cases. Chinese Journal of Neurology [Internet]. 2000 [citado el 23 de mayo de 2021]; 33(4):234-7. Disponible en: https://www.researchgate.net/publication/292789354_Electrophysiological_classification_of_Guillain-Barre_syndrome_Analysis_of_100_cases
103. [Ndiaye](https://pubmed.ncbi.nlm.nih.gov/?term=Ndiaye+M&cauthor_id=14666798) M, [Sène-Diouf](https://pubmed.ncbi.nlm.nih.gov/?term=S%C3%A8ne-Diouf+F&cauthor_id=14666798) F, [Diop](https://pubmed.ncbi.nlm.nih.gov/?term=Diop+AG&cauthor_id=14666798) AG, [Ndao](https://pubmed.ncbi.nlm.nih.gov/?term=Ndao+AK&cauthor_id=14666798) AK, [Diagne](https://pubmed.ncbi.nlm.nih.gov/?term=Diagne+M&cauthor_id=14666798) M, [Thiam](https://pubmed.ncbi.nlm.nih.gov/?term=Thiam+A&cauthor_id=14666798) A, et al. Guillain-Barre syndrome in children: experience in the neurology service of Dakar. Dakar Medical. 2000; 45(1):81-4.
104. Silverman A. Guillain-Barré Syndrome. UCLA Department of Medicine [Internet]. 2000 [citado el 07 de junio de 2021]. Disponible en:<http://www.med.ucla.edu/modules/xfsection/print.php?articleid=157>
105. Paradiso G, Tripoli J, Galicchio S, Fejerman N. Epidemiological, clinical, and electrodiagnostic findings in childhood Guillain-Barré syndrome: A reappraisal. Annals of Neurology. 1999; 46(5):701-7.
106. [Schottlender](https://pubmed.ncbi.nlm.nih.gov/?term=Schottlender+JG&cauthor_id=10752212) JG, [Lombardi](https://pubmed.ncbi.nlm.nih.gov/?term=Lombardi+D&cauthor_id=10752212) D, [Toledo](https://pubmed.ncbi.nlm.nih.gov/?term=Toledo+A&cauthor_id=10752212) A, [Otero](https://pubmed.ncbi.nlm.nih.gov/?term=Otero+C&cauthor_id=10752212) C, [Mazia](https://pubmed.ncbi.nlm.nih.gov/?term=Mazia+C&cauthor_id=10752212) C, [Menga](https://pubmed.ncbi.nlm.nih.gov/?term=Menga+G&cauthor_id=10752212) G. Respiratory failure in the Guillain Barré syndrome. Medicina. 1999; 59(6):705-9.
107. Miralles Alonso F, Gutiérrez Martínez JA, González Sánchez M, Guerra Fernández R. Síndrome Guillain-Barre en la Unidad de Cuidados Intensivos. Revista Cubana de Medicina. 1996; 35(3):152-5.
108. [Drouet A](https://www.scopus.com/authid/detail.uri?authorId=7006890527&eid=2-s2.0-0025979161), [Valance J.](https://www.scopus.com/authid/detail.uri?authorId=6701766779&eid=2-s2.0-0025979161) Miller-Fisher syndrome of ophthalmoplegia, ataxia and areflexia. [Semaine des Hopitaux](https://www.scopus.com/sourceid/18873). 1991; 67(14):470-472.
109. Storey E, Cook M, Peppard R, Newton-John H, Byrne E. Guillain-Barré Syndrome and related conditions in victorian teaching hospitals 1980–84. Australian and New Zealand Journal of Medicine. 1989; 19(6):687-93.
110. Ashrafi MR, Mohammadalipoor A, Naeini AR, Amanat M, Tavasoli AR, Heidari M, et al. Clinical Characteristics and Electrodiagnostic Features of Guillain-Barré Syndrome Among the Pediatric Population. Journal of Child Neurology. 2020; 35(7):448-55.
111. Barzegar M, Toopchizadeh V, Golalizadeh D, Pirani A, Jahanjoo F. A predictive model for respiratory failure and determining the risk factors of prolonged mechanical ventilation in children with guillain-barre syndrome. Iranian Journal of Child Neurology. 2020; 14(3):33-46.
112. Estrade S, Guiomard C, Fabry V, Baudou E, Cances C, Chaix Y, et al. Prognostic factors for the sequelae and severity of Guillain-Barré syndrome in children. Muscle Nerve. 2019; 60(6):716-23.
113. Gupta PK, Singhi P, Singhi S, Kasinathan A, Sankhyan N. How Different is AMAN from AIDP in Childhood GBS? A Prospective Study from North India. Indian Journal of Pediatrics. 2019; 86(4):329-34.
114. Islam Z, Papri N, Ara G, Ishaque T, Alam AU, Jahan I, et al. Risk factors for respiratory failure in Guillain-Barré syndrome in Bangladesh: a prospective study. Annals of Clinical and Translational Neurology. 2019; 6(2):324-32.
115. Tian J, Cao C, Li T, Zhang K, Li P, Liu Y, et al. Electrophysiological subtypes and prognostic factors of Guillain-Barre syndrome in northern China. Frontiers in Neurology [Internet]. 2019 [citado el 23 de mayo de 2021]; 10. Disponible en: https://www.frontiersin.org/articles/10.3389/fneur.2019.00714/full
116. Kalita J, Kumar M, Misra UK. Prospective comparison of acute motor axonal neuropathy and acute inflammatory demyelinating polyradiculoneuropathy in 140 children with Guillain–Barré syndrome in India. Muscle Nerve. 2018; 57(5):761-5.
117. Karalok ZS, Taskin BD, Yanginlar ZB, Gurkas E, Guven A, Degerliyurt A, et al. Guillain-Barré syndrome in children: subtypes and outcome. Child's Nervous System. 2018; 34(11):2291-7.
118. Konuşkan B, Okuyaz Ç, Taşdelen B, Kurul SH, Anlar B. Electrophysiological Subtypes and Prognostic Factors of Childhood Guillain-Barré Syndrome. Arch Neuropsychiatry. 2018; 55(3):199-204.
119. Liu S, Xiao Z, Lou M, Ji F, Shao B, Dai H, et al. Guillain-Barré syndrome in southern China: Retrospective analysis of hospitalised patients from 14 provinces in the area south of the Huaihe River. Journal of Neurology, Neurosurgery and Psychiatry. 2018; 89(6):618-26.
120. van den Berg B, Storm EF, Garssen MJP, Blomkwist-Markens PH, Jacobs BC. Clinical outcome of Guillain-Barré syndrome after prolonged mechanical ventilation. Journal of Neurology, Neurosurgery and Psychiatry. 2018; 89(9):949-54.
121. Eshrif S, Ramadan M, Almejresi A. Outcome Of Children with AFP. TMJ [Internet]. 2017 [citado el 23 de marzo de 2021]; 6(2):34-40. Disponible en: https://www.researchgate.net/publication/344597494_Outcome_Of_Children_with_AFP
122. Peric S, Bozovic I, Bjelica B, Berisavac I, Stojiljkovic O, Basta I, et al. Diabetes mellitus may affect short-term outcome of Guillain-Barré syndrome. Journal of the Peripheral Nervous System. 2017; 22(2):127-30.
123. Walgaard C, Lingsma HF, van Doorn PA, van der Jagt M, Steyerberg EW, Jacobs BC. Tracheostomy or Not: Prediction of Prolonged Mechanical Ventilation in Guillain-Barré Syndrome. Neurocritical Care. 2017; 26(1):6-13.
124. Kalita J, Ranjan A, Misra UK. Outcome of Guillain-Barre syndrome patients with respiratory paralysis. Monthly Journal of the Association of Physicians. 2016; 109(5):319-23.
125. Nagappa M, Netto AB, Taly AB, Kulkarni GB, Umamaheshwara Rao GS, Periyavan S, et al. Electrophysiological observations in critically ill Guillain-Barre syndrome. Neurology India [Internet]. 2016 [citado el 23 de mayo de 2021]; 64(5):914-20. Disponible en: https://neurologyindia.com/article.asp?issn=0028-3886;year=2016;volume=64;issue=5;spage=914;epage=920;aulast=Nagappa
126. Cea G, Jara P, Quevedo F. Características epidemiológicas del síndrome de Guillain-Barré en población chilena: estudio hospitalario en un período de 7 años. Revista médica de Chile. 2015; 143(2):183-9.
127. O-Peña D de la, Robles-Figueroa M, Chávez-Peña Q, Bedolla-Barajas M. Características del síndrome de Guillain-Barré en adultos: resultados de un hospital universitario. Revista Médica del Instituto Mexicano del Seguro Social [Internet]. 2015 [citado el 23 de mayo de 2021]; 53(6):678-85. Disponible en: https://www.medigraphic.com/cgi-bin/new/resumen.cgi?IDARTICULO=62656
128. Varkal M, Uzunhan T, Aydinli N, Ekici B, Cąlişkan M, Özmen M. Pediatric Guillain-Barré syndrome: Indicators for a severe course. Annals of Indian Academy of Neurology [Internet]. 2015 [citado el 23 de mayo de 2021]; 18(1):24-8. Disponible en: https://annalsofian.org/article.asp?issn=0972-2327;year=2015;volume=18;issue=1;spage=24;epage=28;aulast=Varkal
129. Zhang G, Li Q, Zhang R, Wei X, Wang J, Qin X. Subtypes and prognosis of Guillain-Barré syndrome in southwest China. PLoS ONE [Internet]. 2015 [citado el 23 de mayo de 2021]; 10(7). Disponible en: <https://journals.plos.org/plosone/article?id=10.1371/journal.pone.0133520>
130. Hosokawa T, Nakajima H, Unoda K, Yamane K, Doi Y, Ishida S, et al. An electrophysiological classification associated with guillain-barré syndrome outcomes. Journal of Neurology [Internet]. 2014 [citado el 23 de mayo de 2021]; 261(1):1986-93. Disponible en: https://www.researchgate.net/publication/264429792_An_electrophysiological_classification_associated_with_Guillain-Barr_syndrome_outcomes
131. Kalita J, Misra UK, Goyal G, Das M. Guillain-Barré syndrome: subtypes and predictors of outcome from India. Journal of the Peripheral Nervous System. 2014; 19(1):36-43.
132. Sankhyan N, Sharma S, Konanki R, Gulati S. Childhood Guillain-Barré syndrome subtypes in northern India. Journal of Clinical Neuroscience. 2014; 21(3):427-30.
133. Yadegari S, Kazemi N, Nafissi S. Clinical and electrophysiological features of Guillain-Barré syndrome in Iran. J Clin Neurosci Off J Neurosurg Soc Australas. 2014; 21(9):1554-7.
134. El-Beleidy AS, Khattab AA, El-Sherbini SA, El-Adely TZ, Sleem HM, El-Haware RES. Antiganglioside antibodies determine the clinical severity and predict response to therapy in Egyptian children with Guillain-Barré Syndrome. Pediatria Polska [Internet]. 2013 [citado el 23 de mayo de 2021]; 88(3):224-9. Disponible en: https://cyberleninka.org/article/n/472045
135. Verma R, Chaudhari TS, Raut TP, Garg RK. Clinico-electrophysiological profile and predictors of functional outcome in Guillain-Barre syndrome (GBS). Journal of the Neurological Sciences. 2013; 335(1-2):105-11.
136. Carrillo-Pérez DL, García-Ramos G, Ruano-Calderón LÁ, Sosa-Hernández JL, Méndez-Castillo JJ. Síndrome de Guillain-Barré en un hospital de referencia en México. Revista Mexicana de Neurociencia [Internet]. 2012 [citado el 23 de mayo de 2021];13(1):15-21. Disponible en: https://www.medigraphic.com/cgi-bin/new/resumen.cgi?IDARTICULO=44789
137. Dourado ME, Félix RH, da Silva WKA, Queiroz JW, Jeronimo SMB. Clinical characteristics of Guillain-Barré syndrome in a tropical country: A Brazilian experience. Acta Neurologica Scandinavica. 2012; 125(1):47-53.
138. Hu M-H, Chen C-M, Lin K-L, Wang H-S, Hsia S-H, Chou M-L, et al. Risk factors of respiratory failure in children with Guillain-Barré syndrome. Pediatrics and Neonatology. 2012; 53(5):295-9.
139. Akbayram S, Dogan M, Akgün C, Peker E, Sayin R, Aktar F, et al. Clinical features and prognosis with Guillain-Barre syndrome. Annals of Indian Academy of Neurology [Internet]. 2011 [citado el 23 de mayo de 2021]; 14(2):98-102. Disponible en: https://www.annalsofian.org/article.asp?issn=0972-2327;year=2011;volume=14;issue=2;spage=98;epage=102;aulast=Akbayram
140. Fourrier F, Robriquet L, Hurtevent J-F, Spagnolo S. A simple functional marker to predict the need for prolonged mechanical ventilation in patients with Guillain-Barré syndrome. Critical care. 2011; 15(1):R65.
141. Kannan MA, Ch RK, Jabeen SA, Rukmini Mridula K, Rao P, Borgohain R. Clinical, electrophysiological subtypes and antiganglioside antibodies in childhood Guillain-Barré syndrome. Neurology India. 2011; 59(5):727-32.
142. Netto AB, Taly AB, Kulkarni GB, Uma Maheshwara Rao GS, Rao S. Prognosis of patients with Guillain-Barré syndrome requiring mechanical ventilation. Neurology India [Internet]. 2011 [citado el 23 de mayo de 2021]; 59(5):707-11. Disponible en: https://neurologyindia.com/article.asp?issn=0028-3886;year=2011;volume=59;issue=5;spage=707;epage=711;aulast=Netto;type=0
143. Tang J, Dai Y, Li M, Cheng M, Hong S, Jiang L, et al. Guillain-Barré syndrome in Chinese children: A retrospective analysis. Pediatric Neurology. 2011; 45(4):233-7.
144. Lee JH, Sung IY, Rew IS. Clinical presentation and prognosis of childhood Guillain-Barré syndrome. Journal of Paediatrics and Child Health. 2008; 44(7-8):449-54.
145. Toopchizadeh V, Barzegar M. Electrophysiologic features of childhood Guillain-Barré syndrome in Iran. Journal of Pediatric Neurology [Internet]. 2008 [citado el 23 de mayo de 2021]; 6(1):11-6. Disponible en: https://content.iospress.com/articles/journal-of-pediatric-neurology/jpn00201
146. Ito H, Ito H, Fujita K, Kinoshita Y, Takanashi Y, Kusaka H. Phrenic nerve conduction in the early stage of Guillain-Barre syndrome might predict the respiratory failure. Acta Neurologica Scandinavica. 2007; 116(4):255-8.
147. Ortiz-Corredor F, Peña-Preciado M. Use of immunoglobulin in severe childhood Guillain-Barré syndrome. Acta Neurologica Scandinavica. 2007;115(4):289-93.
148. Durand M-C, Porcher R, Orlikowski D, Aboab J, Devaux C, Clair B, et al. Clinical and electrophysiological predictors of respiratory failure in Guillain-Barré syndrome: a prospective study. Lancet Neurology. 2006;5(12):1021-8.
149. Nagasawa K, Kuwabara S, Misawa S, Fujii K, Tanabe Y, Yuki N, et al. Electrophysiological subtypes and prognosis of childhood Guillain-Barré syndrome in Japan. Muscle Nerve. 2006;33(6):766-70.
150. Shafqat S, Khealani BA, Awan F, Abedin SE. Guillain-Barré syndrome in Pakistan: Similarity of demyelinating and axonal variants. European Journal of Neurology. 2006;13(6):662-5.
151. Hung P-L, Chang W-N, Huang L-T, Huang S-C, Chang Y-C, Chang C-J, et al. A clinical and electrophysiologic survey of childhood Guillain- Barré syndrome. Pediatric Neurology. 2004; 30(2):86-91.
152. Kaida K, Kusunoki S, Kanzaki M, Kamakura K, Motoyoshi K, Kanazawa I. Anti-GQ1b antibody as a factor predictive of mechanical ventilation in Guillain-Barré syndrome. Neurology. 2004; 62(5):821-4.
153. Cheng B-C, Chang W-N, Chang C-S, Chee C-Y, Huang C-R, Chen J-B, et al. Guillain-Barré syndrome in southern Taiwan: Clinical features, prognostic factors and therapeutic outcomes. European Journal of Neurology. 2003;10(6):655-62.
154. Durand M-C, Lofaso F, Lefaucheur J-P, Chevret S, Gajdos P, Raphaël J-C, et al. Electrophysiology to predict mechanical ventilation in Guillain-Barré syndrome. European Journal of Neurology. 2003;10(1):39-44.
155. Tekgul H, Serdaroglu G, Tutuncuoglu S. Outcome of axonal and demyelinating forms of Guillain-Barré syndrome in children. Pediatric Neurology. 2003;28(4):295-9.

**APPENDICES.**

**APPENDIX 1: Diagnostic Criteria for Guillain-Barre Syndrome used in this Systematic Review.**

| **Name** | **Diagnostic Criteria** |
| --- | --- |
| Brighton (31) | “Bilateral and flaccid limb weakness, attenuated or absent deep tendon reflexes in limbs with weakness, with an interval of 12 hours to 28 days between onset and nadir of weakness and subsequent clinical plateau, and no alternative diagnosis for weakness” |
| Asbury and Cornblath (32) | A. Progressive motor weakness of more than one limb. The degree ranges from minimal leg weakness, with or without mild ataxia, to total paralysis of the muscles of the four limbs and trunk, bulbar and facial paralysis, and external ophthalmoplegia.  B. Areflexia (loss of tendon spasms). Universal areflexia is the rule, although distal areflexia with definite hyporeflexia of the biceps and knee jerks will suffice if other features are consistent. |
| NINCDS (33) | A. Progressive bilateral arm and leg weakness (initially only the legs may be affected).  B. Absent or diminished tendon reflexes in the affected limbs (at some point in the clinical course). |
| Hadden (34) | Must have one of the following in two nerves:   - Criteria for AIDP:   CV <90% LLN (<85%, if distal amp <50% LLN)  DML >110% ULN (>120%, if distal amp <LLN)  F-wave latency >120% ULN   - Criteria for AMAN:   None of the above except in one nerve if distal amp <10% of LLN  Distal amp <80% in two nerves |
| Ho (34) | Must have one of the following in two nerves:   - Criteria for AIDP:   CV <90% LLN (<85%, if distal amp <50% LLN)  DML >110% ULN (>120%, if distal amp <LLN)  TD Unequivocal  F-wave latency >120% ULN   - Criteria for AMAN:   No evidence of demyelination in the above nerves  Distal amp <80% in two nerves |
| CIE-10 (35) | G61.0 Guillain-Barré syndrome: Acute (post-) infectious polyneuritis. |

**APPENDIX 2: Search Strategies according to Bibliographic Database used**

**MEDLINE/Pubmed:**

| Search number | Search terms  04/01/2021 | Number Found |
| --- | --- | --- |
| 1 | "Guillain-Barre Syndrome"[MeSH Terms] | 5 337 |
| 2 | (#1 OR “Miller Fisher Syndrome” [MeSH Terms]) | 5 337 |
| 3 | (“Mortality” [MeSH Terms] OR “Disability Evaluation” [MeSH Terms] OR “Intubation, Intratracheal” [MeSH Terms] OR “Respiration, Artificial” [MeSH Terms] OR “Length of stay” [MeSH Terms]) | 631 682 |
| 4 | (#2 AND #3) | 362 |
| 5 | (Case Reports[ptyp] OR Clinical Study[ptyp] OR Comparative Study[ptyp] OR Evaluation Study[ptyp] OR Journal Article[ptyp] OR Multicenter Study[ptyp] OR Observational Study[ptyp]) | 30 574 487 |
| 6 | (#4 AND #5) | 356 |
| 7 | (#6 AND "humans"[MeSH Terms]) | 354 |
| 8 | (#7 AND hasabstract[text]) | 308 |

((("Guillain-Barre Syndrome"[MeSH Terms] OR “Miller Fisher Syndrome”[MeSH Terms]) AND (“Mortality”[MeSH Terms] OR “Disability Evaluation”[MeSH Terms] OR “Intubation, Intratracheal” [MeSH Terms] OR “Respiration, Artificial” [MeSH Terms] OR “Length of stay”[MeSH Terms])) AND (Case Reports[ptyp] OR Clinical Study[ptyp] OR Comparative Study[ptyp] OR Evaluation Study[ptyp] OR Journal Article[ptyp] OR Multicenter Study[ptyp] OR Observational Study[ptyp]) AND "humans"[MeSH Terms] AND hasabstract[text]))

**LILACS/SciELO:**

| Search number | Search terms  04/01/2021 | Number Found |
| --- | --- | --- |
| 1 | “Guillain Barre Syndrome” | 242 |
| 2 | “Axonal Neuropathy” | 65 |
| 3 | #2 OR “Demyelinating Polyneuropathy” | 98 |
| 4 | (#1 AND #3) | 242 |
| 5 | “Mortality” OR “Disability” OR “Mechanical Ventilation” OR “Intubation” OR “Outcome” OR “Hospitalization” | 58 948 |
| 6 | (#4 AND #5) | 242 |
| 7 | (Article OR Case Reports OR Review article OR Abstract) | 341 461 |
| 8 | (#6 AND #7) | 208 |
| 9 | “Humans” | 5 928 |
| 10 | (#8 AND #9) | 11 |

((((((((“Guillain Barre Syndrome”) AND (“Axonal Neuropathy”)) OR (“Demyelinating Polyneuropathy”)) AND (“Mortality”) OR (“Disability”)) OR (“Mechanical Ventilation”)) OR (“Intubation”)) OR (“Outcome”)) OR (“Hospitalization”)) AND (Article OR Case Reports OR Review article OR Abstract) AND (“Human”)

**Cochrane database:**

| Search number | Search terms  04/01/2021 | Number Found |
| --- | --- | --- |
| 1 | “Guillain Barre Syndrome” | 386 |
| 2 | “Axonal Neuropathy” | 30 |
| 3 | #2 OR “Demyelinating” | 1 049 |
| 4 | (#1 AND #3) | 77 |
| 5 | “Mortality” OR “Disability” OR “Intubation” OR “Mechanical Ventilation” OR “Respiratory failure” OR “Outcome” OR “Hospitalization” | 617 469 |
| 6 | (#4 AND #5) | 55 |
| 7 | (Cochrane Reviews OR Cochrane Protocols OR Trials OR Clinical Answers) | 1 771 831 |
| 8 | (#6 AND #7) | 55 |
| 9 | (#8 AND humans) | 36 |
| 10 | (#10 AND abstract) | 36 |

((“Guillain Barre Syndrome”) AND (“Axonal Neuropathy” OR “Demyelinating”) AND (“Mortality” OR “Disability” OR “Intubation” OR “Mechanical Ventilation” OR “Respiratory failure” OR “Outcome” OR “Hospitalization”) AND (Cochrane Reviews OR Cochrane Protocols OR Trials OR Clinical Answers) AND (“Humans”) AND (“Abstract”))

**Scopus:**

| Search number | Search terms  04/01/2021 | Number Found |
| --- | --- | --- |
| 1 | TITLE-ABS-KEY ("Guillain barre syndrome") | 37 880 |
| 2 | TITLE-ABS-KEY (“Subtypes”) | 95 8451 |
| 3 | #2 OR TITLE-ABS-KEY (“Miller Fisher”) | 752 664 |
| 4 | #3 OR TITLE-ABS-KEY (“Axonal Neuropathy”) | 34 348 |
| 5 | #1 AND #4 | 4 468 |
| 6 | TITLE-ABS-KEY ("Mortality") OR TITLE-ABS-KEY ("Deaths") OR TITLE-ABS-KEY ("Disability") OR ("Mechanical Ventilation") OR ("Intubation") OR  TITLE-ABS-KEY ("Complications")  OR  TITLE-ABS-KEY ("Outcome") OR TITLE-ABS-KEY ("Consequences") OR  TITLE-ABS-KEY ("Hospitalization") | 3 982 282 |
| 7 | (#5 AND #6) | 1 482 |
| 8 | LIMIT-TO (DOCTYPE, "ar") OR LIMIT-TO (DOCTYPE, "re") | 686 |
| 9 | (#7 AND #8) | 1 146 |
| 10 | LIMIT-TO EXACTKEYWORD, "Human" | 29 544 713 |
| 11 | (#9 AND #10) | 1 142 |

(((TITLE-ABS-KEY ("Guillain barre syndrome")) AND ((TITLE-ABS-KEY (“Subtypes”) OR (TITLE-ABS-KEY (“Miller Fisher”)) OR (TITLE-ABS-KEY (“Axonal Neuropathy”)) AND ((TITLE-ABS-KEY ("Mortality") OR TITLE-ABS-KEY ("Deaths") OR TITLE-ABS-KEY ("Disability") OR TITLE-ABS-KEY ("Mechanical Ventilation") OR TITLE-ABS-KEY ("Intubation") OR TITLE-ABS-KEY ("Complications") OR TITLE-ABS-KEY ("Outcome") OR TITLE-ABS-KEY ("Consequences")  OR  TITLE-ABS-KEY ("Hospitalization")) AND (LIMIT-TO (DOCTYPE, "ar") OR LIMIT-TO (DOCTYPE, "re")) AND (LIMIT-TO (EXACTKEYWORD, "Human")))

**Web of Science database:**

| Search number | Search terms  04/01/2021 | Number Found |
| --- | --- | --- |
| 1 | “Guillain Barre Syndrome” | 6 383 |
| 2 | “Axonal Neuropathy” | 4 1 88 |
| 3 | #2 OR “Demyelinating Polyneuropathy” | 6 150 |
| 4 | (#1 AND #3) | 1 033 |
| 5 | “Mortality” OR “Disability” OR “Intubation” OR “Mechanical Ventilation” OR “Respiratory failure” OR “Outcome” OR “Hospitalization” | 2 546 400 |
| 6 | (#4 AND #5) | 269 |
| 7 | (Article OR Abstract of published item OR Database review OR Review) | 2 913 242 |
| 8 | (#6 AND #7) | 88 |
| 9 | (#8 AND humans) | 10 |

((“Guillain Barre Syndrome”) AND (“Axonal Neuropathy” OR “Demyelinating Polyneuropathy”) AND (“Mortality” OR “Disability” OR “Intubation” OR “Mechanical Ventilation” OR “Respiratory failure” OR “Outcome” OR “Hospitalization”) AND (Article OR Abstract of published item OR Database review OR Review) AND humans)

**Google Scholar (English):**

| Search number | Search terms  04/01/2021 | Number Found |
| --- | --- | --- |
| 1 | “Guillain Barre Syndrome” | 73 000 |
| 2 | “Axonal Neuropathy” | 133 000 |
| 3 | #2 OR “Demyelinating Polyneuropathy” | 41 700 |
| 4 | (#1 AND #3) | 19 700 |
| 5 | “Lethality” OR “Mortality” OR “Disability” OR “Intubation” OR “Mechanical Ventilation” OR “Respiratory failure” OR “Respiratory dysfunction” OR “Prognosis” OR “Outcome” OR “Hospitalization” | 2 590 000 |
| 6 | (#4 AND #5) | 9 040 |

((“Guillain Barre Syndrome”) AND (“Axonal Neuropathy” OR “Demyelinating Polyneuropathy”) AND (“Lethality” OR “Mortality” OR “Disability” OR “Intubation” OR “Mechanical Ventilation” OR “Respiratory failure” OR “Respiratory dysfunction” OR “Prognosis” OR “Outcome” OR “Hospitalization”))

**Google Académico (Spanish):**

| Search number | Search terms  04/01/2021 | Number Found |
| --- | --- | --- |
| 1 | “Síndrome de Guillain Barre” | 11 500 |
| 2 | “Neuropatía Axonal” | 7 450 |
| 3 | #2 OR “Polineuropatía Desmielinizante” | 3 520 |
| 4 | (#1 AND #3) | 1 430 |
| 5 | “Letalidad” OR “Mortalidad” OR “Disabilidad” OR “Intubación” OR “Ventilación Mecánica” OR “Falla Respiratoria” OR “Insuficiencia Respiratoria” OR “Pronóstico” OR “Desenlace” OR “Hospitalización” | 57 100 |
| 6 | (#4 AND #5) | 413 |

((“Síndrome de Guillain Barre”) AND (“Neuropatía Axonal” OR “Polineuropatía Desmielinizante”) AND (“Letalidad” OR “Mortalidad” OR “Disabilidad” OR “Intubación” OR “Ventilación Mecánica” OR “Falla Respiratoria” OR “Insuficiencia Respiratoria” OR “Pronóstico” OR “Desenlace” OR “Hospitalización”))

**APPENDIX 3: Selection Flowchart**

**
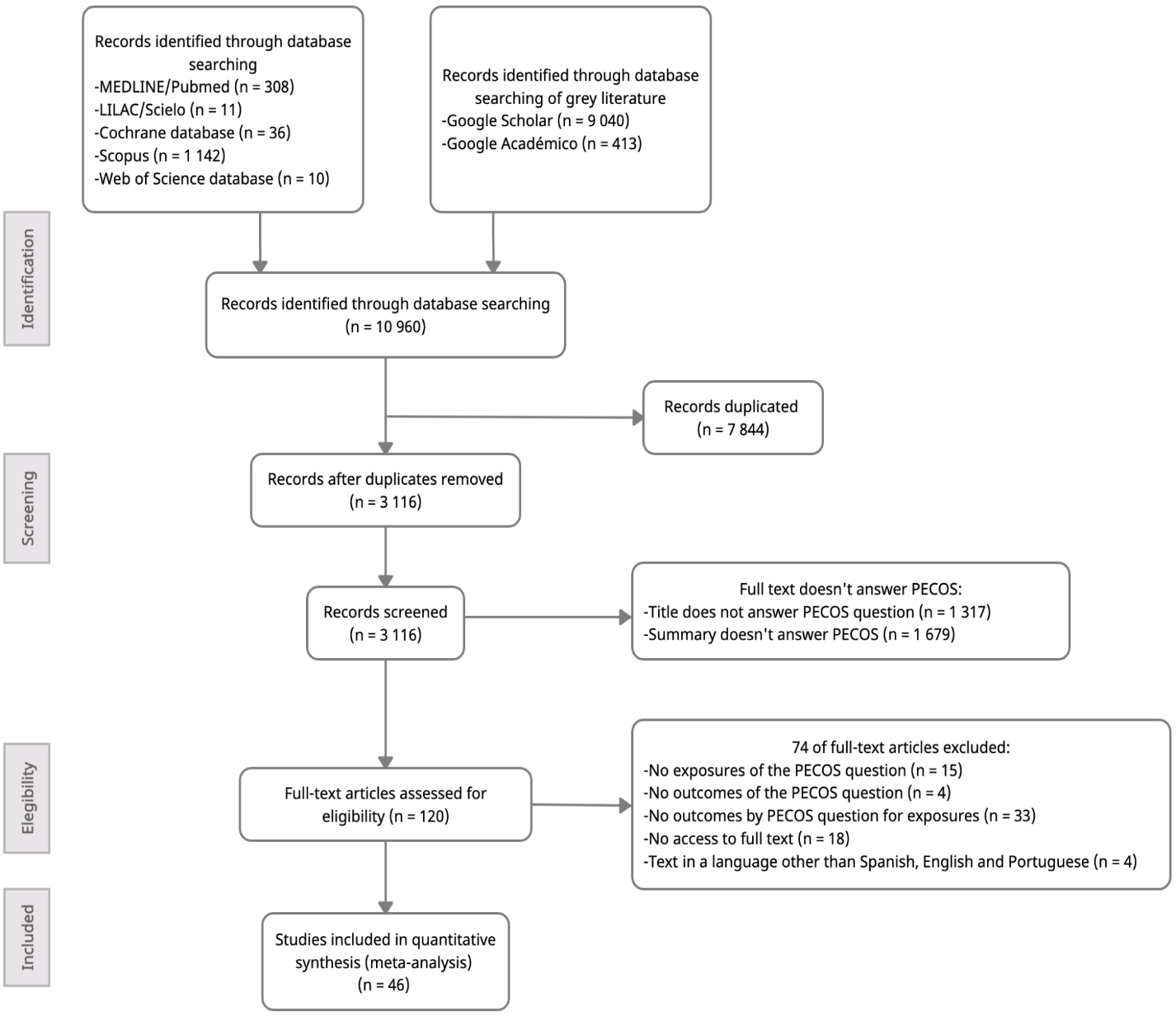
**

**APPENDIX 4: Tables**

**Table 1a.** Excluded studies with reasons for exclusion (74 articles).

| **Number** | **Study** | **Year** | **Country** | **Design** | **Number of**  **Participants** | **Reason for exclusion** |
| --- | --- | --- | --- | --- | --- | --- |
| 1 | Rangan 2021 (36) | 2021 | India | Cohort: retrospective | 30 | No outcomes for exposures |
| 2 | Ruiz-Sandoval 2021 (37) | 2021 | Mexico | Cohort: retrospective | 115 | No access to full text* |
| 3 | Cadenas 2020 (38) | 2020 | Perú | Cohort: retrospective | 105 | No outcomes for exposures |
| 4 | Coya 2020 (39) | 2020 | Perú | Cohort: retrospective | 27 | No outcomes for exposures |
| 5 | Velásquez 2020 (40) | 2020 | Perú | Cohort: retrospective | 92 | No exposures |
| 6 | Bravo 2019 (41) | 2019 | Perú | Cross-Sectional | 32 | No outcomes for exposures |
| 7 | Cabrera-Ortiz 2019 (42) | 2019 | Costa Rica | Cohort: retrospective | 64 | No exposures |
| 8 | Kiliç 2019 (43) | 2019 | Turkey | Cohort: retrospective | 45 | No access to full text* |
| 9 | Sedano 2019 (44) | 2019 | Spain | Cohort: prospective | 56 | No exposures |
| 10 | Vera-Carrasco 2019 (45) | 2019 | Bolivia | Narrative Review | NA | No outcomes for exposures |
| 11 | Yadav 2019 (46) | 2019 | India | Cohort: prospective | 36 | No access to full text* |
| 12 | Yosha-Orpaz 2019 (47) | 2019 | Israel | Cohort | 39 | No outcomes |
| 13 | Zhou 2019* (48) | 2019 | China | Cohort: retrospective | 759 | Text in Chinese* |
| 14 | Aragonès 2018 (49) | 2018 | Spain | Cohort: retrospective | 43 | No outcomes for exposures |
| 15 | Hafsteinsdóttir 2018 (50) | 2018 | Iceland | Cohort: population‐based | 63 | No exposures |
| 16 | Vázquez 2018 (51) | 2018 | Paraguay | Cross-Sectional | 37 | No outcomes for exposures |
| 17 | Ballón-Manrique 2017 (52) | 2017 | Peru | Cohort: retrospective | 16 | No outcomes for exposures |
| 18 | Chareyre 2017 (53) | 2017 | France | Cohort: retrospective | 11 | No outcomes for exposures |
| 19 | Cabrera 2016 (54) | 2016 | Costa Rica | Cohort: retrospective | 64 | No outcomes for exposures |
| 20 | González 2016 (55) | 2016 | Colombia | Case series | 25 | No outcomes |
| 21 | Salehiomran 2016 (56) | 2016 | Iran | Cross-Sectional | 17 | No access to full text* |
| 22 | Wu 2016 (57) | 2016 | China | Cohort: retrospective | 750 | No outcomes for exposures |
| 23 | Zhang 2016* (58) | 2016 | China | Cohort: retrospective | 118 | Text in Chinese* |
| 24 | Pasanen 2015 (59) | 2015 | United States | Narrative Review | NA | No access to full text* |
| 25 | Ramos 2015 (60) | 2015 | Perú | Cross-Sectional | 31 | No outcomes for exposures |
| 26 | Bhagat 2014 (61) | 2014 | India | Cohort: retrospective | 59 | No outcomes for exposures |
| 27 | Gonzalez 2014 (62) | 2014 | Cuba | Cohort: prospective | 25 | No outcomes for exposures |
| 28 | Montes de Oca 2014 (63) | 2014 | Cuba | Cohort: retrospective | 12 | No outcomes for exposures |
| 29 | Pandian 2014 (64) | 2014 | India | Cohort: prospective | 30 | No outcomes for exposures |
| 30 | Ye 2014 (65) | 2014 | China | Cohort: retrospective | 138 | No outcomes |
| 31 | Gazioglu 2013 (66) | 2013 | Turkey | Cohort: retrospective | 64 | No access to full text* |
| 32 | González-Suárez 2013 (67) | 2013 | Spain | Cohort: retrospective | 106 | No outcomes for exposures |
| 33 | Uribarri 2013 (68) | 2013 | Spain | Cohort: retrospective | 17 | No exposures |
| 34 | Witsch 2013 (69) | 2013 | Germany | Cohort | 110 | No exposures |
| 35 | Lin 2012 (70) | 2012 | Taiwan | Cohort: retrospective | 43 | No exposures |
| 36 | Paul 2012 (71) | 2012 | India | Cohort: retrospective and prospective cohort | 138 | No exposures |
| 37 | Qiu 2012 (72) | 2012 | China | Cohort: retrospective | 14 | No access to full text* |
| 38 | Alexander 2011 (73) | 2011 | India | Cohort: retrospective | 115 | No outcomes for exposures |
| 39 | Incecik 2011 (74) | 2011 | Turkey | Cohort: retrospective | 46 | No outcomes for exposures |
| 40 | Areeyapinan 2010 (75) | 2010 | Thailand | Cohort: retrospective | 55 | No access to full text* |
| 41 | Mendez 2010 (76) | 2010 | Mexico | Cohort: retrospective | 132 | No exposures |
| 42 | Pithadia 2010 (77) | 2010 | India | Narrative Review | NA | No outcomes for exposures |
| 43 | Van der Linden 2010 (78) | 2010 | Brazil | Cohort: retrospective | 61 | No exposures |
| 44 | Ye 2010 (79) | 2010 | China | Cohort: retrospective | 26 | No outcomes for exposures |
| 45 | Aguilar 2009 (80) | 2009 | Honduras | Cross-Sectional | 29 | No outcomes for exposures |
| 46 | Dididze 2009 (81) | 2009 | Georgia | Cross-Sectional | 57 | No access to full text* |
| 47 | Kollár 2009 (82) | 2009 | Hungary | Cohort: retrospective | 35 | No access to full text* |
| 48 | [Ramírez](https://pesquisa.bvsalud.org/portal/?lang=pt&q=au:%22Ram%C3%ADrez%20Zamora,%20M%22) 2009 (83) | 2009 | El Salvador | Cohort: retrospective | 414 | No access to full text* |
| 49 | Caballero 2008 (84) | 2008 | Honduras | Cohort: prospective | 12 | No exposures |
| 50 | Dhar 2008 (85) | 2008 | Canada | Cohort: retrospective | 76 | No outcomes for exposures |
| 51 | Moreno 2008 (86) | 2008 | Cuba | Cohort: retrospective | 73 | No outcomes for exposures |
| 52 | Zazula 2008 (87) | 2008 | Czech Republic | Narrative Review | NA | No outcomes for exposures |
| 53 | Markoula 2007 (88) | 2007 | Greece | Case series | 46 | No outcomes for exposures |
| 54 | Nachamkin 2007 (89) | 2007 | México | Cohort: prospective | 121 | No outcomes for exposures |
| 55 | Ortiz-Corredor 2007 (90) | 2007 | Colombia | Cohort | 332 | No outcomes for exposures |
| 56 | Sinha 2007 (91) | 2007 | India | Cohort: prospective | 80 | No outcomes for exposures |
| 57 | Arami 2006 (92) | 2006 | Iran | Cohort: prospective | 76 | No outcomes for exposures |
| 58 | Dai 2006 (93) | 2006 | China | Cohort: retrospective | 254 | No access to full text* |
| 59 | França 2005 (94) | 2005 | Brazil | Cohort: retrospective | 18 | No outcomes for exposures |
| 60 | Ryan 2005 (95) | 2005 | Australia | Narrative Review | NA | No exposures |
| 61 | Sundar 2005 (96) | 2005 | India | Cohort: retrospective and prospective cohort | 46 | No access to full text* |
| 62 | Chroni 2004 (97) | 2004 | Greece | Cohort: retrospective | 105 | No exposures |
| 63 | Hiraga 2003 (98) | 2003 | Japan | Cohort | 131 | No outcomes |
| 64 | Nishimoto 2001* (99) | 2001 | NA | NA | NA | Text in Japanese* |
| 65 | Téllez-Zenteno 2001 (100) | 2001 | Mexico | Cohort: retrospective | 28 | No access to full text* |
| 65 | Fletcher 2000 (101) | 2000 | USA | Cohort: retrospective | 114 | No exposures |
| 67 | Jiang 2000 (102) | 2000 | China | Cohort | 100 | No outcomes for exposures |
| 68 | Ndiaye 2000* (103) | 2000 | Senegal | Cohort: retrospective | 19 | Text in French* |
| 69 | Silverman 2000 (104) | 2000 | NA | NA | NA | No access to full text* |
| 70 | Paradiso 1999 (105) | 1999 | Argentina | Cross-Sectional | 61 | No access to full text* |
| 71 | Schottlender 1999 (106) | 1999 | Argentina | Cohort: retrospective | 44 | No access to full text* |
| 72 | Miralles 1996 (107) | 1996 | Cuba | Cohort | 8 | No exposures |
| 73 | Drouet 1991 (108) | 1991 | Francia |  |  | No access to full text* |
| 74 | Storey 1989 (109) | 1989 | Australia | Cohort: retrospective | 110 | No outcomes for exposures |

NA: Not applicable
 * Every effort was made to contact the author, but no response was obtained.

**Table 1b.** Characteristics of the INCLUDED studies (46 articles).

| **Number** | **Study** | **Year** | **Country** | **Design** | **Population** | **Participants** | **Exposure** | **Outcomes** | **Diagnostic Criteria** |
| --- | --- | --- | --- | --- | --- | --- | --- | --- | --- |
| 1 | Ashrafi 2020 (110) | 2020 | Iran | Case series: prospective | Children | 30 | - AIDP  - AMAN | - MV* | Clinical criteria |
| 2 | Barzegar 2020 (111) | 2020 | Iran | Cross-sectional | Children | 272 | - AIDP  - Axonal  - Normal  - Unclassified | - MV*  - Time of MV | Asbury and Cornblath |
| 3 | Estrade 2019 (112) | 2019 | France | Cohort: retrospective multicenter | Children | 92 | - Demyelinating form  - Axonal form | - MV*  - Time of hospital stay  - ICU stay | Medical records |
| 4 | Gupta 2019 (113) | 2019 | India | Case series: prospective | Children | 40 | - AIDP  - AMAN | - MV*  - Time of MV  - Time of hospital stay | Hughes criteria |
| 5 | Islam 2019 (114) | 2019 | Bangladesh | Cohort:  prospective | Children and adults | 429 | - AIDP  - AMAN  - AMSAN  - Unclassified  - Normal | - MV*  - Death | NINCDS |
| 6 | Tian 2019 (115) | 2019 | China | Cohort: retrospective | Adults | 80 | - AIDP  - AMAN | - MV*  - Time of hospital stay - Disability | NINCDS |
| 7 | Kalita 2018 (116) | 2018 | India | Case series: prospective | Children | 128 | - AIDP  - AMAN | - MV*  - Time of hospital stay | Asbury and Cornblath |
| 8 | Karalok 2018 (117) | 2018 | Turkey | Cohort: retrospective | Children | 49 | - AIDP  - AMAN  - AMSAN  - MFS | - MV* | Clinical criteria |
| 9 | Konuşkan 2018 (118) | 2018 | Turkey | Cohort: retrospective | Children | 166 | - Demyelinating  - Axonal:  - AMAN - AMSAN | - MV*  - Time of hospital stay | Asbury and Cornblath |
| 10 | Liu 2018 (119) | 2018 | China | Cohort: retrospective | Children and adults | 448 | - AIDP  - AMAN | - MV*  - Time of hospital stay - Disability  - Death | Asbury and Cornblath |
| 11 | Van den Berg 2018 (120) | 2018 | Netherlands | Cohort:  prospective | Adults | 188 | - Demyelinating  - Axonal  - Inexcitable - Equivocal | - MV*  - Time of MV* | Asbury and Cornblath |
| 12 | Eshrif 2017 (121) | 2017 | Lybia | Case series: retrospective | Children | 23 | - AIDP  - AMAN  - AMSAN  - Normal | - MV* | Medical records |
| 13 | Peric 2017 (122) | 2017 | Serbia | Cohort: retrospective | Adults | 198 | - Demyelinating  - Axonal | - Disability | Asbury and Cornblath |
| 14 | Walgaard 2017 (123) | 2017 | Netherlands | Cohort: prospective | Adults | 68 | - AIDP  - AMAN  - Inexcitable  - Equivocal | - MV* | Hadden |
| 15 | Kalita 2016 (124) | 2016 | India | Cohort: prospective | Children and Adults | 88 | - AIDP  - AMAN  - AMSAN  - Inexcitable  - Equivocal | - MV* | NINCDS |
| 16 | Nagappa 2016 (125) | 2016 | India | Cohort:  retrospective | Children and Adults | 63 | - Primary demyelinating  - Primary axonal  - Inexcitable  - Equivocal | - Disability  - Death | Hadden |
| 17 | Cea 2015 (126) | 2015 | Chile | Cohort: retrospective | Children and Adults | 41 | - AIDP  - AMAN  - AMSAN  - ASAN  - MFS | - MV*  - Time of hospital stay - Disability | CIE-10 |
| 18 | de la O-Peña 2015 (127) | 2015 | México | Cohort: retrospective | Adults | 45 | - AIDP  - AMAN  - AMSAN  - MFS | - Time of hospital stay - Death | Asbury and Cornblath |
| 19 | Varkal 2015 (128) | 2015 | Turkey | Cohort: retrospective | Children | 40 | - AIDP  - AMAN | - Time of MV  - Time of hospital stay - Time of ICU stay | Medical records |
| 20 | Zhang 2015 (129) | 2015 | China | Cohort: prospective | Children and adults | 146 | - AIDP  - AMAN  - MFS | - MV*  - Disability | Medical records |
| 21 | Hosokawa 2014 (130) | 2014 | Japan | Cohort: retrospective | Adults | 34 | - M-AIDP pattern  - AMAN pattern | - Disability | Electrophysiological criteria |
| 22 | Kalita 2014 (131) | 2014 | India | Cohort: prospective | Children and Adults | 286 | - AIDP  - AMAN | - MV*  - Death | NINCDS |
| 23 | Sankhyan 2014 (132) | 2014 | India | Cohort: prospective | Children | 45 | - Acute motor axonal neuropathy  - Acute inflammatory demyelinating polyneuropathy | - MV* | Hadden, Asbury and Cornblath |
| 24 | Yadegari 2014 (133) | 2014 | Iran | Cohort: retrospective | Adults | 114 | - AIDP - AMAN  - AMSAN | - Time of hospital stay  - ICU admission  - Time of ICU stay  - Death | Asbury and Cornblath |
| 25 | El-Beleidy 2013 (134) | 2013 | Egipto | Cohort: prospective | Children | 43 | - AIDP  - AMAN | - MV* | Asbury and Cornblath |
| 26 | Verma 2013 (135) | 2013 | India | Cohort: prospective | Children and Adults | 90 | - Primary demyelinating  - Axonal | - MV*  - Disability | Asbury and Cornblath |
| 27 | Carrillo-Pérez 2012 (136) | 2012 | México | Cohort: retrospective | Adults | 23 | - AIDP  - AMAN  - AMSAN  - MFS | - Time of hospital stay - Time of ICU stay | Asbury and Cornblath |
| 28 | Dourado 2012 (137) | 2012 | Brazil | Cohort: retrospective | Children and adults | 149 | - AIDP  - Axonal | - Time of MV*  - Time of hospital stay - Disability  - Death | Asbury and Cornblath |
| 29 | Hu 2012 (138) | 2012 | Taiwan | Cohort: retrospective | Children | 40 | - AIDP  - AMAN  - AMSAN  - MFS | - MV* | Medical records |
| 30 | Akbayram 2011 (139) | 2011 | Turkey | Cohort: retrospective | Children | 36 | - AIDP  - AMAN  - AMSAN | - MV*  - Time of hospital stay  - Death | Clinical criteria |
| 31 | Fourrier 2011 (140) | 2011 | France | Cohort:  retrospective | Adults | 48 | - Demyelinating type | - MV* | Clinical criteria |
| 32 | Kannan 2011 (141) | 2011 | India | Cohort:  prospective | Children | 41 | - AIDP  - AMAN  - Unclassified | - MV* | Asbury and Cornblath |
| 33 | Netto 2011 (142) | 2011 | India | Cohort: retrospective | Children and Adults | 87 | - Primary demyelinating  - Primary axonal  - Inexcitable  - Equivocal | - Disability  - Death | NINCDS |
| 34 | Tang 2011 (143) | 2011 | China | Cohort: retrospective | Children | 248 | - AIDP  - AMAN  - AMSAN  - Inexcitable  - Unclassified | - Disability | Asbury and Cornblath |
| 35 | Lee 2008 (144) | 2008 | Korea | Cohort: retrospective | Children | 56 | - AIDP  - AMAN  - AMSAN  - MFS | - MV* | Medical records |
| 36 | Toopchizadeh 2008 (145) | 2008 | Iran | Cohort: prospective | Children | 72 | - Axonal type  - Demyelinating type | - MV* - Disability  - Death | Asbury and Cornblath |
| 37 | Ito 2007 (146) | 2007 | Japan | Case series: prospective | Children and Adults | 11 | - Demyelinating  - Axonal  - Equivocal | - MV*  - Disability | Asbury and Cornblath |
| 38 | Ortiz-Corredor 2007 (147) | 2007 | Colombia | Cohort: retrospective | Children | 48 | - AMAN  - AIDP | - Time of MV* | Asbury and Cornblath |
| 39 | Durand 2006 (148) | 2006 | France | Cohort: prospective | Adults | 100 | - Demyelinating  - Axonal  - Equivocal  - Normal | - MV* | Asbury and Cornblath |
| 40 | Nagasawa 2006 (149) | 2006 | Japan | Cohort: retrospective | Children | 26 | - AIDP  - AMAN | - MV*  - Disability | Electrodiagnostic criteria reported by Ho and colleagues |
| 41 | Shafqat 2006 (150) | 2006 | Pakistan | Cohort: retrospective | Children and adults | 135 | - Axonal  - Demyelinating  - Ambiguous | - MV*  - Time of MV  - Time of hospital stay | Asbury and Cornblath |
| 42 | Hung 2004 (151) | 2004 | Taiwan | Cohort: retrospective | Children | 21 | - AIDP - Axonal  - MFS  - Unclassified | - MV*  - Time of hospital stay  - Disability | Asbury and Cornblath |
| 43 | Kaida 2004 (152) | 2004 | Japan | Case-control | Adults | 43 | - Primary demyelinating  - Primary axonal  - Inexcitable  - Normal  - Equivocal | - MV* | Asbury and Cornblath |
| 44 | Cheng 2003 (153) | 2003 | Taiwan | Cohort: retrospective | Children and adults | 90 | - AIDP  - Axonal  - MFS  - Unclassified | - MV*  - Time of MV - Time of hospital stay - Disability | Asbury and Cornblath |
| 45 | Durand 2003 (154) | 2003 | France | Cohort: prospective | Adults | 42 | - Demyelinating  - Axonal  - Equivocal | - MV* | Asbury and Cornblath |
| 46 | Tekgul 2003 (155) | 2003 | Turkey | Cohort: retrospective | Children | 23 | - AIDP  - AMAN  - AMSAN | - Time of MV* - Time of hospital stay  - Time of ICU stay | Asbury and Cornblath |

*MV: Mechanical ventilation

**Table 2:** Quality assessment of all the studies included using the NewCasttle Ottawa scale (NOS).

|  |  |  | **Selection** | | | | **Comparability** | **Outcome** | | |  |
| --- | --- | --- | --- | --- | --- | --- | --- | --- | --- | --- | --- |
| **Number** | **Study** | **Study Type** | **Representativeness of the exposed cohort** | **Selection of the non-exposed cohort** | **Ascertainment of exposure** | **Demonstration that the current outcome of interest was not present at start of study** | **Comparability of cohorts on the basis of the design of analysis** | **Assessment of outcome** | **Was follow-up long enough for outcomes to occur** | **Adequacy of follow-up of cohorts** | **NOS**  **Score** |
| 1 | Ashrafi 2020 | Case series: prospective | **✵** | **✵** | **✵** | **✵** | **✵✵** | **✵** | **✵** | **✵** | 9 |
| 2 | Barzegar 2020 | Cross-sectional | **✵** | **✵** | **✵** |  | **✵✵** | **✵** | **✵** | **✵** | 8 |
| 3 | Estrade 2019 | Cohort: retrospective multicenter | **✵** | **✵** | **✵** | **✵** | **✵✵** | **✵** | **✵** | **✵** | 9 |
| 4 | Gupta 2019 | Case series: prospective | **✵** | **✵** | **✵** |  | **✵✵** | **✵** | **✵** | **✵** | 8 |
| 5 | Islam 2019 | Cohort:  prospective | **✵** | **✵** | **✵** |  | **✵✵** | **✵** | **✵** | **✵** | 8 |
| 6 | Tian 2019 | Cohort: retrospective | **✵** | **✵** | **✵** | **✵** | **✵✵** | **✵** | **✵** | **✵** | 9 |
| 7 | Kalita 2018 | Case series: prospective | **✵** | **✵** | **✵** |  | **✵✵** | **✵** | **✵** | **✵** | 8 |
| 8 | Karalok 2018 | Cohort: retrospective | **✵** | **✵** | **✵** |  | **✵✵** | **✵** | **✵** | **✵** | 8 |
| 9 | Konuşkan 2018 | Cohort: retrospective | **✵** | **✵** | **✵** |  | **✵✵** | **✵** | **✵** | **✵** | 8 |
| 10 | Liu 2018 | Cohort: retrospective | **✵** | **✵** | **✵** |  | **✵✵** | **✵** | **✵** | **✵** | 8 |
| 11 | Van den Berg 2018 | Cohort:  prospective | **✵** | **✵** | **✵** |  |  | **✵** | **✵** | **✵** | 6 |
| 12 | Eshrif 2017 | Case series: retrospective | **✵** | **✵** | **✵** |  |  | **✵** | **✵** | **✵** | 6 |
| 13 | Peric 2017 | Cohort: retrospective | **✵** | **✵** | **✵** |  |  | **✵** | **✵** | **✵** | 6 |
| 14 | Walgaard 2017 | Cohort:  retrospective | **✵** | **✵** | **✵** |  |  | **✵** | **✵** | **✵** | 6 |
| 15 | Kalita 2016 | Cohort: prospective | **✵** | **✵** | **✵** |  |  | **✵** | **✵** | **✵** | 6 |
| 16 | Nagappa 2016 | Cohort: prospective | **✵** | **✵** | **✵** |  |  | **✵** | **✵** |  | 5 |
| 17 | Cea 2015 | Cohort: retrospective | **✵** | **✵** | **✵** |  | **✵✵** | **✵** | **✵** | **✵** | 8 |
| 18 | de la O-Peña 2015 | Cohort: retrospective | **✵** | **✵** | **✵** |  | **✵✵** | **✵** | **✵** |  | 7 |
| 19 | Varkal 2015 | Cohort: retrospective | **✵** | **✵** | **✵** |  |  | **✵** | **✵** | **✵** | 6 |
| 20 | Zhang 2015 | Cohort: prospective | **✵** | **✵** | **✵** |  | **✵✵** | **✵** | **✵** | **✵** | 8 |
| 21 | Hosokawa 2014 | Cohort: retrospective | **✵** | **✵** | **✵** |  | **✵✵** | **✵** | **✵** | **✵** | 8 |
| 22 | Kalita 2014 | Cohort: prospective | **✵** | **✵** | **✵** |  | **✵✵** | **✵** | **✵** | **✵** | 8 |
| 23 | Sankhyan 2014 | Cohort: prospective | **✵** | **✵** | **✵** |  | **✵✵** | **✵** | **✵** | **✵** | 8 |
| 24 | Yadegari 2014 | Cohort: retrospective | **✵** | **✵** | **✵** |  | **✵✵** | **✵** | **✵** | **✵** | 8 |
| 25 | El-Beleidy 2013 | Cohort: prospective | **✵** | **✵** | **✵** |  | **✵✵** | **✵** | **✵** |  | 7 |
| 26 | Verma 2013 | Cohort: prospective | **✵** | **✵** | **✵** |  |  | **✵** | **✵** | **✵** | 6 |
| 27 | Carrillo-Pérez 2012 | Cohort: retrospective | **✵** | **✵** | **✵** |  |  | **✵** | **✵** | **✵** | 6 |
| 28 | Dourado 2012 | Cohort: retrospective | **✵** | **✵** | **✵** |  | **✵✵** | **✵** | **✵** | **✵** | 8 |
| 29 | Hu 2012 | Cohort: retrospective | **✵** | **✵** | **✵** |  |  | **✵** | **✵** | **✵** | 6 |
| 30 | Akbayram 2011 | Cohort: retrospective | **✵** | **✵** | **✵** |  | **✵✵** | **✵** | **✵** | **✵** | 8 |
| 31 | Fourrier 2011 | Cohort:  retrospective | **✵** | **✵** | **✵** |  |  | **✵** | **✵** | **✵** | 6 |
| 32 | Kannan 2011 | Cohort:  prospective | **✵** | **✵** | **✵** |  |  | **✵** | **✵** |  | 5 |
| 33 | Netto 2011 | Cohort: retrospective | **✵** | **✵** | **✵** |  |  | **✵** | **✵** |  | 5 |
| 34 | Tang 2011 | Cohort: retrospective | **✵** | **✵** | **✵** |  | **✵✵** | **✵** | **✵** |  | 7 |
| 35 | Lee 2008 | Cohort: retrospective | **✵** | **✵** | **✵** |  | **✵✵** | **✵** | **✵** |  | 7 |
| 36 | Toopchizadeh 2008 | Cohort: prospective | **✵** | **✵** | **✵** |  | **✵✵** | **✵** | **✵** |  | 7 |
| 37 | Ito 2007 | Case series: prospective | **✵** | **✵** | **✵** |  | **✵✵** | **✵** | **✵** |  | 7 |
| 38 | Ortiz-Corredor 2007 | Cohort: retrospective | **✵** | **✵** | **✵** |  |  | **✵** | **✵** |  | 5 |
| 39 | Durand 2006 | Cohort: prospective | **✵** | **✵** | **✵** |  |  | **✵** | **✵** |  | 5 |
| 40 | Nagasawa 2006 | Cohort: retrospective | **✵** | **✵** | **✵** |  | **✵✵** | **✵** | **✵** | **✵** | 8 |
| 41 | Shafqat 2006 | Cohort: retrospective | **✵** | **✵** | **✵** |  | **✵✵** | **✵** | **✵** |  | 7 |
| 42 | Hung 2004 | Cohort: retrospective | **✵** | **✵** | **✵** |  | **✵✵** | **✵** | **✵** |  | 7 |
| 43 | Kaida 2004 | Case-control **†** | **✵** | **✵** |  | **✵** | **✵✵** | **✵** | **✵** |  | 7 |
| 44 | Cheng 2003 | Cohort: retrospective | **✵** | **✵** | **✵** |  | **✵✵** | **✵** | **✵** | **✵** | 8 |
| 45 | Durand 2003 | Cohort: prospective | **✵** | **✵** | **✵** |  | **✵✵** | **✵** | **✵** |  | 7 |
| 46 | Tekgul 2003 | Cohort: retrospective | **✵** | **✵** | **✵** |  | **✵✵** | **✵** | **✵** | **✵** | 8 |

All studies were evaluated using the Newcastle Ottawa scale (NOS) for cohorts, except for **†** that used the version for case control studies.

**Table 3a.** Study Results reporting Duration of MV in days with dispersion measures (8 articles).

| **Number** | **Study** | **Exposure** | **Outcomes**  **(Time in days)** | **Axonal Variants**  **(AMAN, AMSAN, SMF)** | | | **Demyelination Variants (AIDP, non-specified)** | | | **Mean Difference** | **95% CI of Mean Difference** |
| --- | --- | --- | --- | --- | --- | --- | --- | --- | --- | --- | --- |
|  |  |  |  | **Mean** | **SD** | **Total^1^** | **Mean** | **SD** | **Total^1^** |  |  |
| 1 | Barzegar 2020 | GBS Type | Time of MV | 26.68 | 5.31 | 124 | 8.22 | 2.51 | 148 | +18.46 | Not estimated |
| 2 | Gupta 2019 | GBS Type | Time of MV | 30 | 35 | 20 | 32 | 31 | 20 | -2 | Not estimated |
| 3 | Varkal 2015 | GBS Type | Time of MV | 30.5 | 20.5 | 19 | 6.5 | 0.8 | 21 | +24 | Not estimated |
| 4 | Dourado 2012 | GBS Type | Time of MV | 7.65 | ^ | 27 | 4.25 | ^ | 122 | +3.4 | Not estimated |
| 5 | Ortiz-Corredor 2007 | GBS Type | Time of MV | 16.5* | ^ | 32 | 13* | ^ | 16 | +3.5 | 0.72 – 3.30 |
| 6 | Shafqat 2006 | GBS Type | Time of MV | 19 | 11 | 55 | 27 | 43 | 80 | -8 | Not estimated |
| 7 | Cheng 2003 | GBS Type | Time of MV | 26 | ^ | 13 | 18.2 | ^ | 77 | +7.8 | Not estimated |
| 8 | Tekgul 2003 | GBS Type | Time of MV | 6.84 | 4.36 | 13 | 0.2 | 0 | 10 | +6.64 | Not estimated |

SD: Standard Deviation. CI: Confidence Interval. GBS: Guillain-Barre Syndrome. MV: Mechanical ventilation.

1 Total Number of Patients per group

* Calculated based on tables numbers.

^ For meta-analysis calculations, pooled SD of the remaining studies was employed replacing missing data.

**Table 3b.** Study Results reporting ICU Stay Means in days with dispersion measures (5 articles).

| **Number** | **Study** | **Exposure** | **Outcomes**  **(Time in days)** | **Axonal Variants**  **(AMAN, AMSAN, SMF)** | | | **Demyelination Variants (AIDP, non-specified)** | | | **Mean Difference** | **95% CI of Mean Difference** |
| --- | --- | --- | --- | --- | --- | --- | --- | --- | --- | --- | --- |
|  |  |  |  | **Mean** | **SD** | **Total^1^** | **Mean** | **SD** | **Total^1^** |  |  |
| 1 | Estrade 2019 | GBS Type | Time of ICU Stay | 3 | ^ | 20 | 18 | ^ | 72 | -15 | Not estimated |
| 2 | Varkal 2015 | GBS Type | Time of ICU Stay | 18.8 | 26 | 19 | 5.4 | 6 | 21 | +13.4 | Not estimated |
| 3 | Yadegari 2014 | GBS Type | Time of ICU Stay | 90 | 6 | 47 | 13 | 22 | 67 | +77 | Not estimated |
| 4 | Carrillo-Pérez 2012 | GBS Type | Time of ICU Stay | 11.8 | ^ | 17 | 5 | ^ | 6 | +6.8 | Not estimated |
| 5 | Tekgul 2003 | GBS Type | Time of ICU Stay | 14.38 | 20.6 | 13 | 1.3 | 1.5 | 10 | +13.08 | Not estimated |

SD: Standard Deviation. CI: Confidence Interval. GBS: Guillain-Barre Syndrome. ICU: Intensive Care Unit.

1 Total Number of Patients per group

^ For meta-analysis calculations, pooled SD of the remaining studies was employed replacing missing data.

**Table 3c.** Study Results reporting Hospital Stay Means in days with dispersion measures (17 articles).

| **Number** | **Study** | **Exposure** | **Outcomes**  **(Time in days)** | **Axonal Variants**  **(AMAN, AMSAN, SMF)** | | | **Demyelination Variants (AIDP, non-specified)** | | | **Mean Difference** | **95% CI of Mean Difference** |
| --- | --- | --- | --- | --- | --- | --- | --- | --- | --- | --- | --- |
|  |  |  |  | **Mean** | **SD** | **Total^1^** | **Mean** | **SD** | **Total^1^** |  |  |
| 1 | Tian 2019 | GBS Type | Time of Hospital Stay | 14 | 8 | 22 | 11 | 4 | 58 | +3 | Not estimated |
| 2 | Estrade 2019 | GBS Type | Time of Hospital Stay | 11.4 | ^ | 20 | 12.6 | ^ | 72 | -1.2 | Not estimated |
| 3 | Gupta 2019 | GBS Type | Time of Hospital Stay | 16 | 22 | 20 | 11 | 12 | 20 | +5 | Not estimated |
| 4 | Konuşkan 2018 | GBS Type | Time of Hospital Stay | 17 | 23 | 82 | 13.4 | 8.4 | 84 | +3.6 | Not estimated |
| 5 | Kalita 2018 | GBS Type | Time of Hospital Stay | 22 | 26.8 | 33 | 13.2 | 10.5 | 95 | +8.8 | Not estimated |
| 6 | Liu 2018 | GBS Type | Time of Hospital Stay | 19.2 | 9.7 | 124 | 17.7 | 10.9 | 324 | +1.46 | Not estimated |
| 7 | Cea 2015 | GBS Type | Time of Hospital Stay | 26.4* | 28.9** | 14 | 18.7* | 27.4** | 27 | +7.7 | Not estimated |
| 8 | de la O-Peña 2015 | GBS Type | Time of Hospital Stay | 9.85* | 24.6** | 41 | 14* | 13.5** | 4 | -4.15 | Not estimated |
| 9 | Varkal 2015 | GBS Type | Time of Hospital Stay | 22.1 | 18.3 | 19 | 11.4 | 5.7 | 21 | +10.7 | Not estimated |
| 10 | Yadegari 2014 | GBS Type | Time of Hospital Stay | 16.3*** | ^ | 47 | 12.8 | 5.5 | 67 | +3.5 | Not estimated |
| 11 | Carrillo-Pérez 2012 | GBS Type | Time of Hospital Stay | 21.3 | ^ | 17 | 10.7 | ^ | 6 | +10.6 | Not estimated |
| 12 | Dourado 2012 | GBS Type | Time of Hospital Stay | 31 | 23.9 | 27 | 22.5 | 13.26 | 122 | +8.5 | Not estimated |
| 13 | Akbayram 2011 | GBS Type | Time of Hospital Stay | 3*** | ^ | 11 | 10*** | ^ | 25 | -7 | Not estimated |
| 14 | Shafqat 2006 | GBS Type | Time of Hospital Stay | 14 | 17 | 55 | 15 | 24 | 80 | -1 | Not estimated |
| 15 | Hung 2004 | GBS Type | Time of Hospital Stay | 21*** | ^ | 3 | 12.6 | ^ | 18 | +8.4 | Not estimated |
| 16 | Cheng 2003 | GBS Type | Time of Hospital Stay | 15.95*** | ^ | 13 | 25.4 | 18.72 | 77 | -9.45 | Not estimated |
| 17 | Tekgul 2003 | GBS Type | Time of Hospital Stay | 27.2 | 30.47 | 13 | 13.1 | 3.3 | 10 | +14.1 | Not estimated |

SD: Standard Deviation. CI: Confidence Interval. GBS: Guillain-Barre Syndrome.

1 Total Number of Patients per group

* Median

** Interquartilic interval divided by 1.35.

*** Calculated based on tables numbers.

^ For meta-analysis calculations, pooled SD of the remaining studies was employed replacing missing data.

**Table 3d.** Study results reporting disability after the event as a Hughes score with dispersion event measures (13 articles).

| **Number** | **Study** | **Exposure** | **Outcomes**  **(Time in days)** | **Axonal Variants**  **(AMAN, AMSAN, SMF)** | | | **Demyelination Variants (AIDP, non-specified)** | | | **Mean Difference** | **95% CI of Mean Difference** |
| --- | --- | --- | --- | --- | --- | --- | --- | --- | --- | --- | --- |
|  |  |  |  | **Mean** | **SD** | **Total^1^** | **Mean** | **SD** | **Total^1^** |  |  |
| 1 | Liu 2018 | GBS Type | Hughes score at nadir | 3.59 | 0.75 | 124 | 3.19 | 1.16 | 324 | +0.4 | Not estimated |
| 2 | Zhang 2015 | GBS Type | Hughes score at nadir | 3.70*** | ^ | 49 | 3.71 | 0.88 | 97 | -0.01 | Not estimated |
| 3 | Tang 2011 | GBS Type | Hughes score at nadir | 3.49*** | ^ | 146 | 3.25 | 1.04 | 102 | +0.24 | Not estimated |
| 4 | Nagasawa 2006 | GBS Type | Hughes score at nadir | 4* | 2.22** | 15 | 4* | 1.48** | 11 | 0 | Not estimated |
| 5 | Hung 2004 | GBS Type | Hughes score at nadir | 4*** | ^ | 3 | 3.2 | ^ | 18 | +0.8 | Not estimated |
| 6 | Cheng 2003 | GBS Type | Hughes score at nadir | 3*** | ^ | 13 | 3.55 | 1.16 | 77 | -0.55 | Not estimated |
| 7 | Liu 2018 | GBS Type | Hughes score  at discharge | 2.66 | 1.13 | 124 | 1.75 | 1.26 | 324 | +0.91 | Not estimated |
| 8 | Zhang 2015 | GBS Type | Hughes score  at 3 months | 1.57*** | ^ | 49 | 1.20 | 1.18 | 97 | +0.37 | Not estimated |
| 9 | Zhang 2015 | GBS Type | Hughes score  at 6 months | 1.08*** | ^ | 49 | 0.62 | 0.88 | 97 | +0.46 | Not estimated |
| 10 | Nagasawa 2006 | GBS Type | Hughes score  at 6 months | 1.1* | 2.96** | 15 | 0.7* | 1.48** | 11 | +0.4 | Not estimated |
| 11 | Kannan 2011 | GBS Type | Hughes score mean | 3.6 | 0.7 | 19 | 3.7 | 0.8 | 22 | -0.1 | Not estimated |
| 12 | Hung 2004 | GBS Type | Hughes score  at admission | 3*** | ^ | 3 | 2.9 | ^ | 18 | +0.1 | Not estimated |
| 13 | Cheng 2003 | GBS Type | Hughes score  at admission | 2.77*** | ^ | 13 | 2.96 | 0.9 | 77 | -0.19 | Not estimated |

SD: Standard Deviation. CI: Confidence Interval. GBS: Guillain-Barre Syndrome.

1 Total Number of Patients per group

* Median

** Interquartilic interval divided by 1.35.

*** Calculated based on tables numbers.

^ For meta-analysis calculations, pooled SD of the remaining studies was employed replacing missing data.

**Table 3e.** Study results reporting disability after the event as a dichotomous variable based on the Hughes score (13 articles).

| **Number** | **Study** | **Intervention** | **Outcomes** | **Axonal Variants**  **(AMAN, AMSAN, SMF)** | | **Demyelination Variants (AIDP, non-specified)** | | **RR** | **95% CI** |
| --- | --- | --- | --- | --- | --- | --- | --- | --- | --- |
|  |  |  |  | **Events** | **Total^1^** | **Events** | **Total^1^** |  |  |
| 1 | Ito 2007 | GBS Type | Disability: Hughes score at enrollment ≥3 | 4 | 6 | 5 | 5 | 0.67 | Not estimated |
| 2 | Hosokawa 2014 | GBS Type | Disability: Hughes score at first EMG ≥3 | 3 | 6 | 18 | 28 | 0.78 | Not estimated |
| 3 | Tian 2019 | GBS Type | Disability: Hughes score at discharge ≥3 | 28 | 58 | 11 | 22 | 0.97 | Not estimated |
| 4 | Peric 2017 | GBS Type | Disability: Hughes score at discharge >3 | 17 | 46 | 36 | 152 | 1.56 | Not estimated |
| 5 | Nagappa 2016 | GBS Type | Disability: Hughes score at discharge >3 | 1 | 1 | 37 | 62 | 0.00 | Not estimated |
| 6 | Cea 2015 | GBS Type | Disability: Hughes score at discharge ≥3 | 8 | 14 | 9 | 27 | 1.71 | Not estimated |
| 7 | Netto 2011 | GBS Type | Disability: Hughes score at discharge >3 | 0 | 1 | 49 | 86 | 0.00 | Not estimated |
| 8 | Peric 2017 | GBS Type | Disability: Hughes score at nadir >3 | 30 | 46 | 88 | 152 | 1.13 | Not estimated |
| 9 | Verma 2013 | GBS Type | Disability: Hughes score at nadir ≥3 | 25 | 60 | 0 | 30 | 0.00 | Not estimated |
| 10 | Dourado 2012 | GBS Type | Disability: Hughes score at nadir >3 | 19 | 27 | 108 | 122 | 0.79 | Not estimated |
| 11 | Tang 2011 | GBS Type | Disability: Hughes score at nadir ≥3 | 114 | 146 | 67 | 102 | 1.19 | Not estimated |
| 12 | Toopchizadeh 2008 | GBS Type | Disability: Hughes score at nadir >3 | 32 | 38 | 22 | 34 | 1.30 | Not estimated |
| 13 | Durand 2006 | GBS Type | Disability: Hughes score >1 at 6 months | 3 | 10 | 31 | 90 | 0.87 | Not estimated |

RR: Relative Risk. CI: Confidence Interval. GBS: Guillain-Barre Syndrome.

1 Total Number of Patients per group

**Table 3f.** Study Results reporting proportion of people admitted to ICU (1 article).

| **Number** | **Study** | **Intervention** | **Outcomes** | **Axonal Variants**  **(AMAN, AMSAN, SMF)** | | **Demyelination Variants (AIDP, non-specified)** | | **RR** | **95% CI** |
| --- | --- | --- | --- | --- | --- | --- | --- | --- | --- |
|  |  |  |  | **Events** | **Total^1^** | **Events** | **Total^1^** |  |  |
| 1 | Yadegari 2014 | GBS Type | ICU admission | 8 | 47 | 10 | 67 | 1.14 | Not estimated |

RR: Relative Risk. CI: Confidence Interval. GBS: Guillain-Barre Syndrome. ICU: Intensive Care Unit.

1 Total Number of Patients per group

**Table 3g.** Study Results reporting proportion of people requiring MV (34 articles).

| **Number** | **Study** | **Intervention** | **Outcomes** | **Axonal Variants**  **(AMAN, AMSAN, SMF)** | | **Demyelination Variants (AIDP, non-specified)** | | **RR** | **95% CI** |
| --- | --- | --- | --- | --- | --- | --- | --- | --- | --- |
|  |  |  |  | **Events** | **Total^1^** | **Events** | **Total^1^** |  |  |
| 1 | Ashrafi 2020 | GBS Type | MV requirement | 0 | 18 | 1 | 12 | 0.00 | Not estimated |
| 2 | Barzegar 2020 | GBS Type | MV requirement | 20 | 124 | 11 | 148 | 2.15** | 1.01 - 4.69 |
| 3 | Estrade 2019 | GBS Type | MV requirement (ICU) | 3 | 20 | 18 | 72 | 0.60 | Not estimated |
| 4 | Gupta 2019 | GBS Type | MV requirement | 6 | 20 | 2 | 20 | 3.00 | Not estimated |
| 5 | Islam 2019 | GBS Type | MV requirement | 55 | 295 | 17 | 134 | 1.50 | Not estimated |
| 6 | Tian 2019 | GBS Type | MV requirement at admission | 3 | 58 | 3 | 22 | 0.38 | Not estimated |
| 7 | Kalita 2018 | GBS Type | MV requirement | 8 | 33 | 13 | 95 | 1.78 | Not estimated |
| 8 | Karalok 2018 | GBS Type | MV requirement | 0 | 22 | 0 | 27 | 0.00 | Not estimated |
| 9 | Konuşkan 2018 | GBS Type | MV requirement | 10 | 82 | 8 | 84 | 1.28 | Not estimated |
| 10 | Liu 2018 | GBS Type | MV requirement | 7 | 124 | 39 | 324 | 0.46 | Not estimated |
| 11 | Van den Berg 2018 | GBS Type | MV requirement ≤61 days | 3 | 94 | 51 | 94 | 0.06 | Not estimated |
| 12 | Eshrif 2017 | GBS Type | MV requirement | 2 | 19 | 0 | 4 | 0.00 | Not estimated |
| 13 | Walgaard 2017 | GBS Type | MV requirement >14 days | 4 | 4 | 48 | 64 | 1.33 | Not estimated |
| 14 | Kalita 2016 | GBS Type | MV requirement | 13 | 23 | 22 | 65 | 1.67 | Not estimated |
| 15 | Cea 2015 | GBS Type | MV requirement | 2 | 14 | 1 | 27 | 3.86 | Not estimated |
| 16 | Zhang 2015 | GBS Type | MV requirement | 14 | 49 | 11 | 97 | 2.53 | Not estimated |
| 17 | Kalita 2014 | GBS Type | MV requirement | 7 | 44 | 25 | 242 | 1.54 | 1.70 - 7.54 |
| 18 | Sankhyan 2014 | GBS Type | MV requirement | 1 | 30 | 2 | 15 | 0.23 | Not estimated |
| 19 | El-Beleidy 2013 | GBS Type | MV requirement | 17 | 23 | 8 | 20 | 1.85 | Not estimated |
| 20 | Verma 2013 | GBS Type | MV requirement | 11 | 60 | 4 | 30 | 1.38 | Not estimated |
| 21 | Hu 2012 | GBS Type | MV requirement | 2 | 11 | 2 | 29 | 2.64 | Not estimated |
| 22 | Akbayram 2011 | GBS Type | MV requirement | 2 | 11 | 1 | 25 | 4.55 | Not estimated |
| 23 | Fourrier 2011 | GBS Type | MV requirement | 9 | 15 | 31 | 33 | 0.64 | Not estimated |
| 24 | Kannan 2011 | GBS Type | MV requirement | 2 | 19 | 2 | 22 | 1.17 | Not estimated |
| 25 | Lee 2008 | GBS Type | MV requirement | 3 | 22 | 5 | 34 | 0.93 | Not estimated |
| 26 | Toopchizadeh 2008 | GBS Type | MV requirement | 9 | 38 | 3 | 34 | 2.69 | Not estimated |
| 27 | Ito 2007 | GBS Type | MV requirement | 1 | 6 | 0 | 5 | 0.00 | Not estimated |
| 28 | Durand 2006 | GBS Type | MV requirement | 0 | 10 | 29 | 90 | 0.00 | Not estimated |
| 29 | Nagasawa 2006 | GBS Type | MV requirement | 4 | 15 | 0 | 11 | 0.00 | Not estimated |
| 30 | Shafqat 2006 | GBS Type | MV requirement | 16 | 55 | 13 | 80 | 1.78 | Not estimated |
| 31 | Hung 2004 | GBS Type | MV requirement | 1 | 3 | 0 | 18 | 0.00 | Not estimated |
| 32 | Kaida 2004 | GBS Type | MV requirement | 2 | 5 | 16 | 38 | 0.95 | Not estimated |
| 33 | Cheng 2003 | GBS Type | MV requirement | 3 | 13 | 23 | 77 | 0.77 | Not estimated |
| 34 | Durand 2003 | GBS Type | MV requirement | 0 | 5 | 17 | 37 | 0.00 | Not estimated |

RR: Relative Risk. CI: Confidence Interval. GBS: Guillain-Barre Syndrome. MV: Mechanical Ventilation.

1 Total Number of Patients per group

** Odds ratio

**Table 3h.** Study Results reporting proportion of people who died (10 articles).

| **Number** | **Study** | **Intervention** | **Outcomes** | **Axonal Variants**  **(AMAN, AMSAN, SMF)** | | **Demyelination Variants (AIDP, non-specified)** | | **RR** | **95% CI** |
| --- | --- | --- | --- | --- | --- | --- | --- | --- | --- |
|  |  |  |  | **Events** | **Total^1^** | **Events** | **Total^1^** |  |  |
| 1 | Liu 2018 | GBS Type | Death during hospitalization | 2 | 124 | 1 | 324 | 5.33 | Not estimated |
| 2 | Nagappa 2016 | GBS Type | Death during hospitalization | 0 | 1 | 1 | 62 | 0.00 | Not estimated |
| 3 | Yadegari 2014 | GBS Type | Death during hospitalization | 0 | 47 | 4 | 67 | 0.00 | Not estimated |
| 4 | Dourado 2012 | GBS Type | Death during hospitalization | 2 | 27 | 6 | 122 | 1.51 | Not estimated |
| 5 | Akbayram 2011 | GBS Type | Death during hospitalization | 1 | 11 | 2 | 25 | 1.14 | Not estimated |
| 6 | Netto 2011 | GBS Type | Death during hospitalization | 0 | 1 | 1 | 86 | 0.00 | Not estimated |
| 7 | Toopchizadeh 2008 | GBS Type | Death during hospitalization | 2 | 38 | 0 | 34 | 0.00 | Not estimated |
| 8 | Islam 2019 | GBS Type | Death at 6 months | 10 | 295 | 3 | 134 | 1.51 | Not estimated |
| 9 | Kalita 2014 | GBS Type | Death at 3 months | 1 | 44 | 6 | 242 | 0.92 | Not estimated |
| 10 | de la O-Peña 2015 | GBS Type | Death during follow up | 4 | 41 | 1 | 4 | 0.39 | Not estimated |

RR: Relative Risk. CI: Confidence Interval. GBS: Guillain-Barre Syndrome.

1 Total Number of Patients per group

**APPENDIX 5: FIGURES**

**Figure 1a:** Death Funnel Plot according to subgroups.


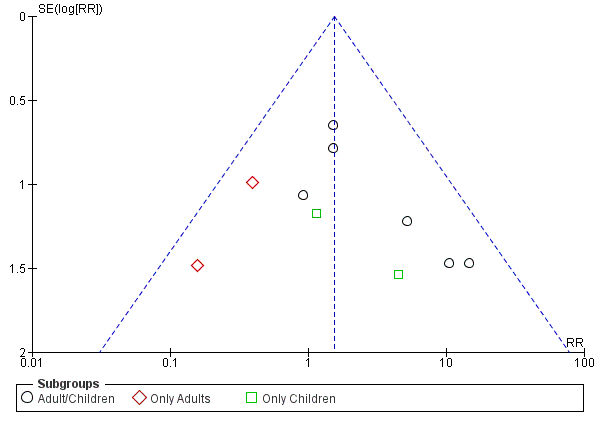


**Figure 1b:** Disability (as dichotomous variable) Funnel Plot according to subgroups.


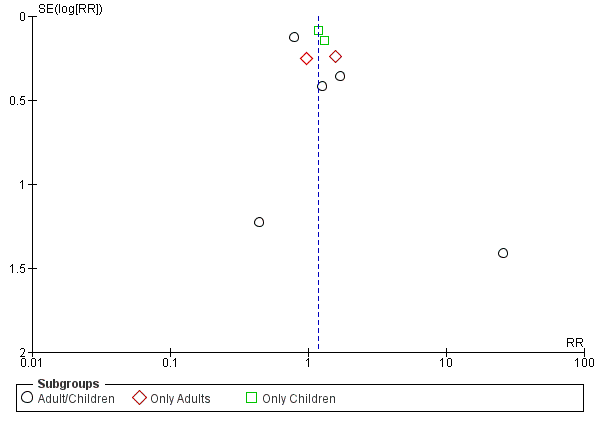


**Figure 1c:** Time of hospital stay Funnel Plot according to subgroups.


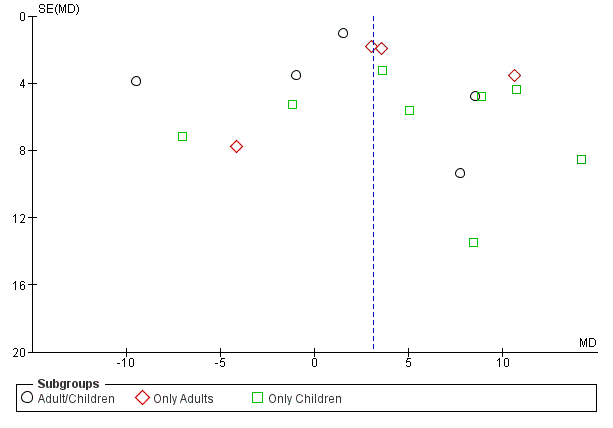


**Figure 1d:** MV requirement Funnel Plot according to subgroups.


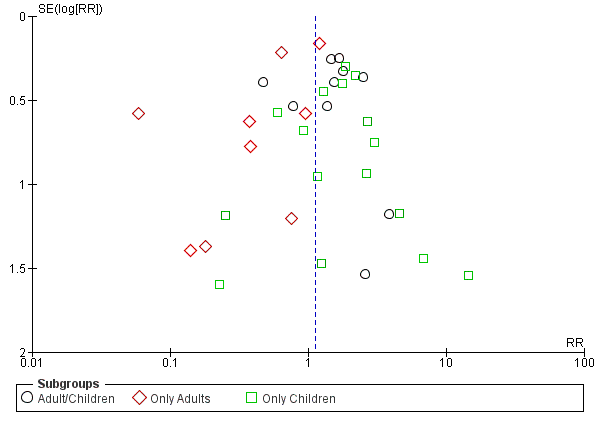


**Figure 2a:** Time of MV Forest Plot according to subgroups.


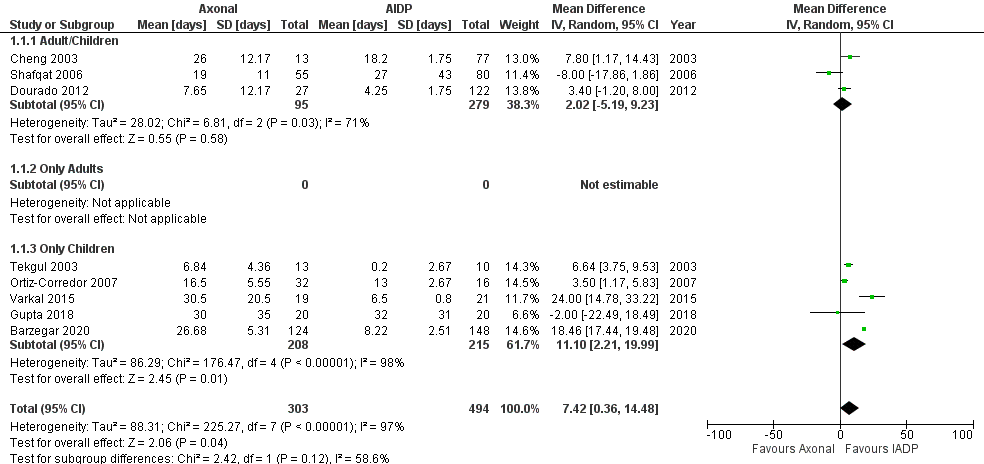


CI: Confidence Interval.

**Figure 2b:** Time of ICU stay Forest Plot according to subgroups.


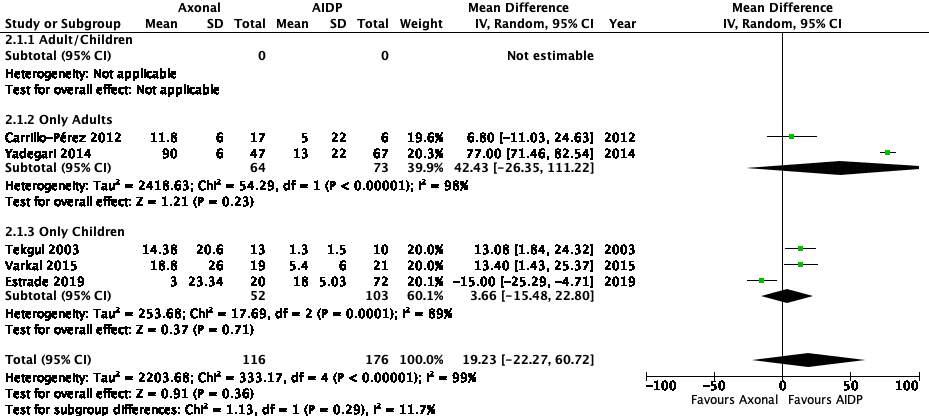


CI: Confidence Interval.

**Figure 2c:** Time of hospital stay Forest Plot according to subgroups.


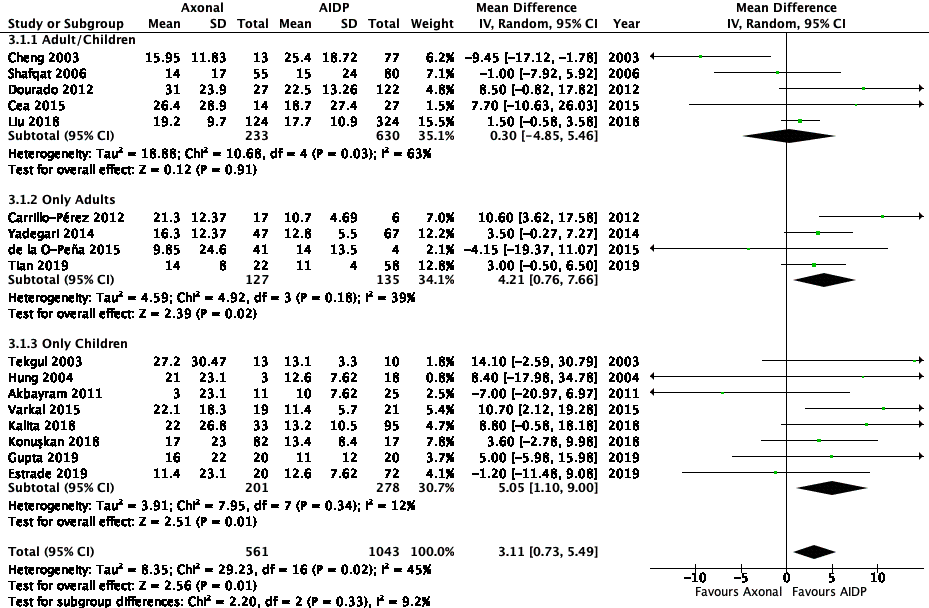


CI: Confidence Interval.**Figure 2d:** Disability (Hughes score) with dispersion measures Forest Plot according to subgroups.


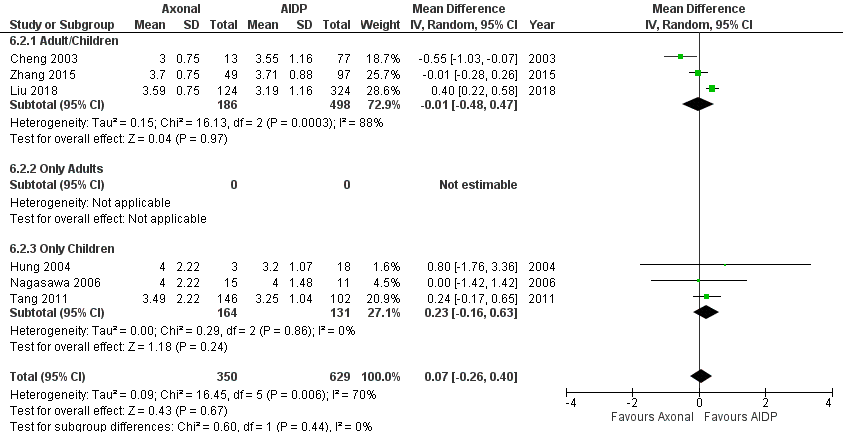


CI: Confidence Interval.

**Figure 2e:** Disability (Hughes score) Forest Plot according to subgroups.


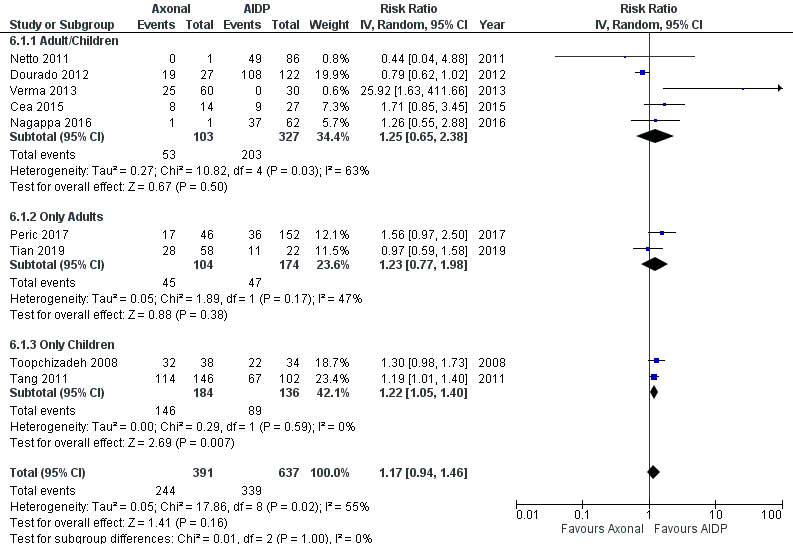


CI: Confidence Interval.**Figure 2f:** MV requirement Forest Plot according to subgroups.


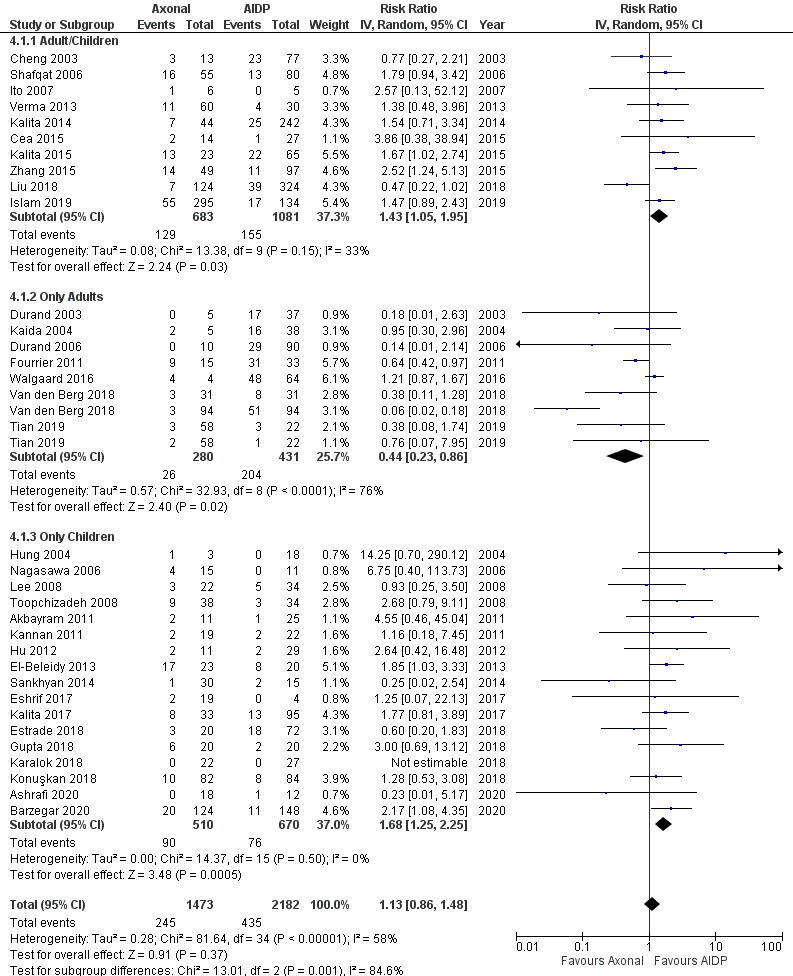


CI: Confidence Interval.

**Figure 2g:** Death Forest Plot according to subgroups.


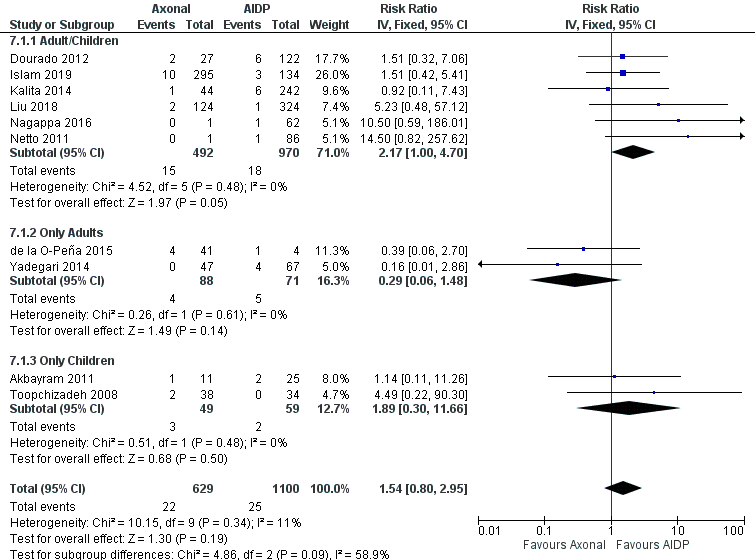


CI: Confidence Interval.

**APPENDIX 6: SENSITIVITY ANALYSIS**

**Figure 3a:** Sensitivity analysis for Time of Mechanical Ventilation according to subgroups**
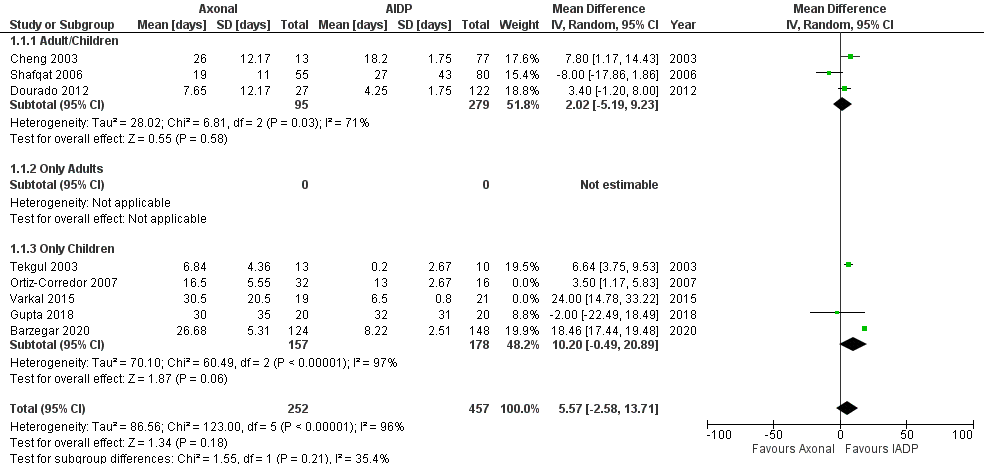
**

*A weight of 0% was given to articles with NOS <7.

**Figure 3b:** Sensitivity analysis for Time of Hospitalization according to subgroups

**
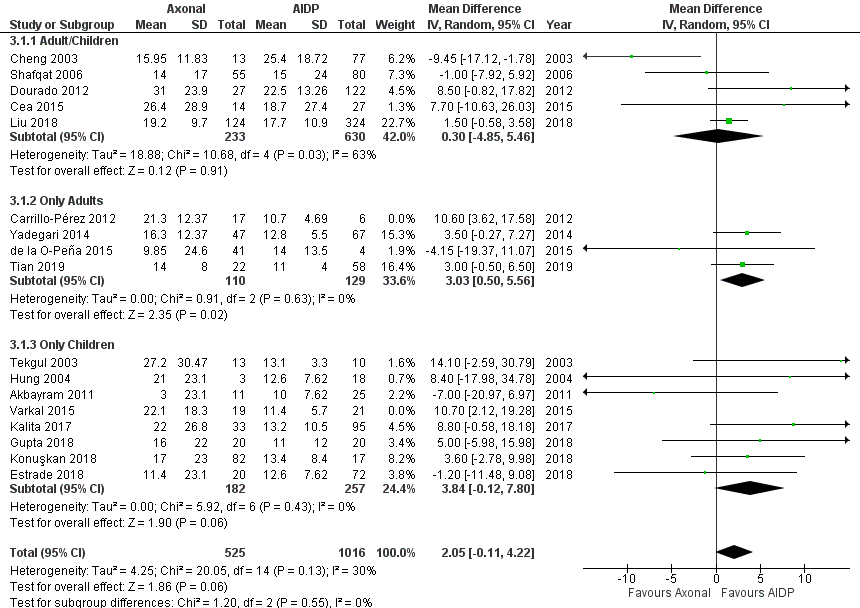
**

*A weight of 0% was given to articles with NOS <7.

**Figure 3c:** Sensitivity analysis for Disability according to subgroups

**
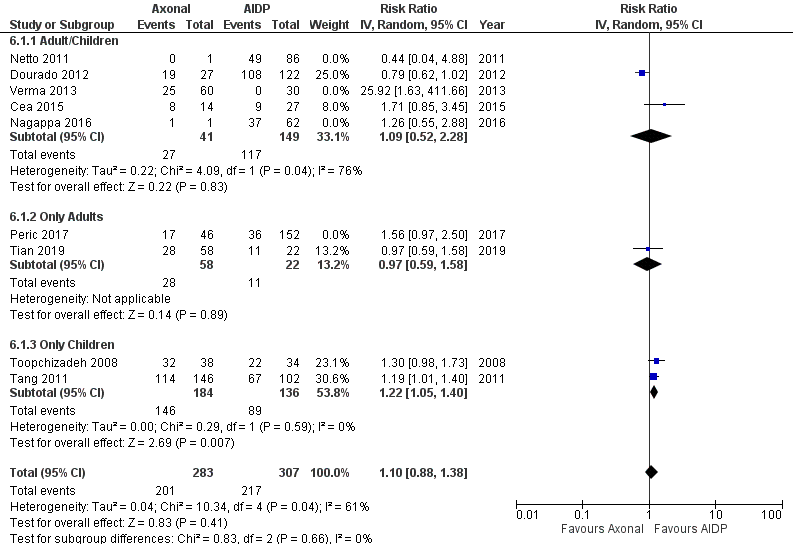
**

*A weight of 0% was given to articles with NOS <7.

**Figure 3d:** Sensitivity analysis for Mechanical Ventilation requirement according to subgroups

**
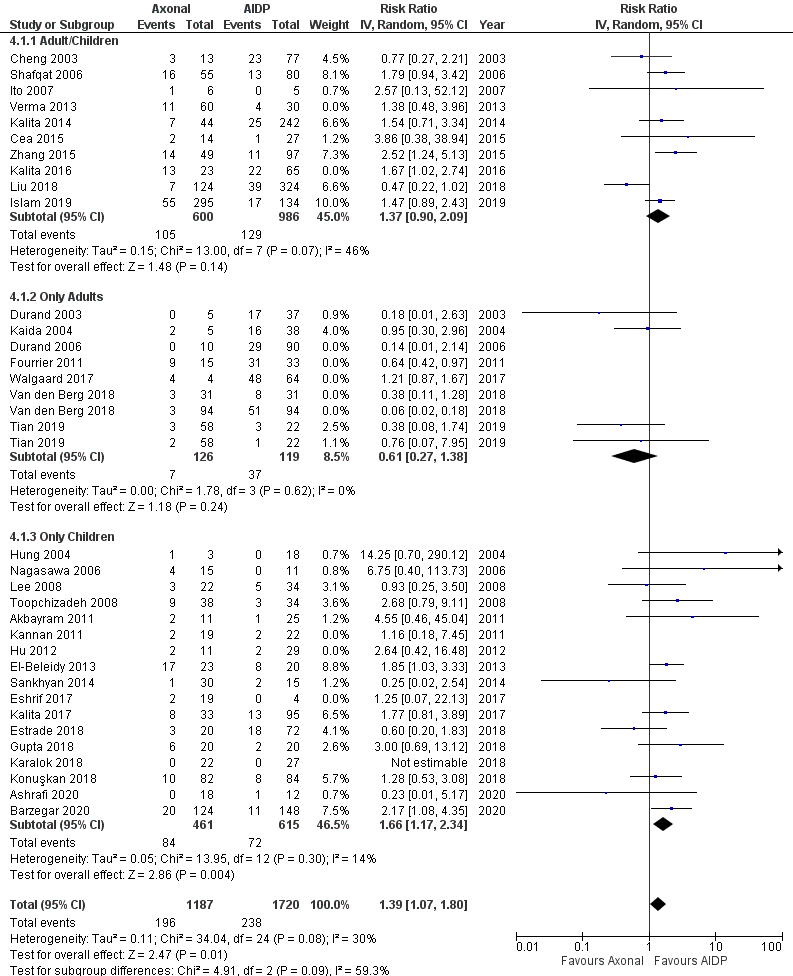
**

*A weight of 0% was given to articles with NOS <7.

**Figure 3e:** Sensitivity analysis for Mortality according to subgroups

**
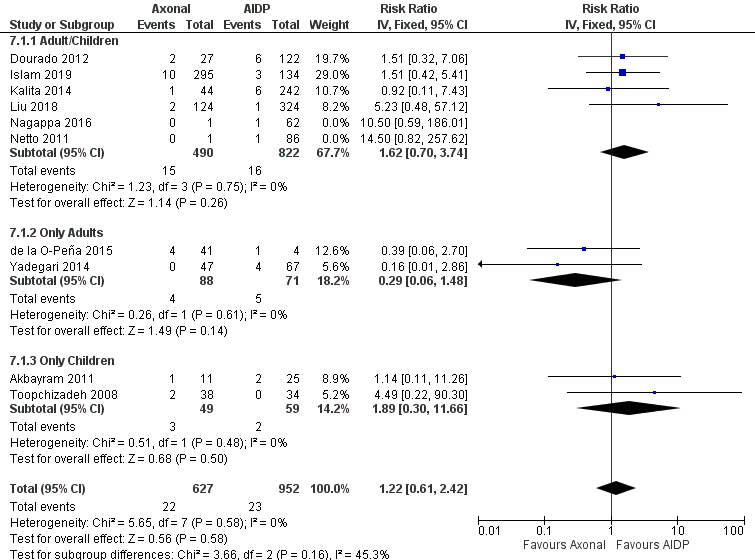
**

*A weight of 0% was given to articles with NOS <7.
