# Supplementary material for: Mechanical ventilation, hospitalization time, deaths and disability according to the variants of Guillain-Barré syndrome: systematic review and meta-analysis
Source: Rev Peru Med Exp Salud Publica. 2024 Aug 28;41(3):247–58. doi: 10.17843/rpmesp.2024.413.13509 (PMC11495932; doi:10.17843/rpmesp.2024.413.13509)
Supplement: Translation

# Translation: Ventilación mecánica, tiempo de hospitalización, muertes y discapacidad según las variantes del síndrome de Guillain-Barré: revisión sistemática y metaanálisis

**Authors:** Silvana Ximena Castro Diaz, Luiza Pereira-Salto, Roger Vladimir Araujo Castillo

**Affiliations:** 1 Universidad Peruana de Ciencias Aplicadas, Lima, Perú.

**Keywords:** Síndrome de Guillain-Barré, Neuropatía Autoinmune Aguda, Polineuropatía Desmielinizante Inflamatoria Aguda, Ventilación mecánica, Hospitalización, Unidad de Cuidados intensivos, Mortalidad, Evaluación de la Discapacidad

## Abstract

**Objetivos.:**

Determinar el requerimiento y tiempo para ventilación mecánica y Unidad de Cuidados Intensivos (UCI), hospitalización y tiempo de hospitalización, muerte y discapacidad de las variantes axonales del Síndrome de Guillain-Barré (SGB) en comparación con la variante aguda desmielinizante en pacientes de todas las edades.

**Materiales y métodos.:**

Revisión sistemática que incluyó pacientes con SGB; la exposición fueron las variantes axonales y el comparador la polineuropatía desmielinizante inflamatoria aguda (AIDP) los desenlaces fueron el requerimiento y tiempo en ventilación mecánica (VM), requerimiento y tiempo en la UCI, tiempo de hospitalización, discapacidad y muerte. Se utilizó la escala NewCasttle-Ottawa (NOS) para evaluar el riesgo de sesgo. Se realizó un metaanálisis para calcular las diferencias de medias y los riesgos relativos (RR) con sus intervalos de confianza (IC) del 95% utilizando varianzas inversas y modelos de efectos aleatorios.

**Resultados.:**

De los 3116 artículos encontrados, 46 cumplieron los criterios de selección. El tiempo en VM fue 7,42 días (IC95%: 0,36 a 1,48) y el tiempo de hospitalización fue 3,11 (IC95%: 0,73 a 5,49) días en las variantes axonales. Las variantes axonales tuvieron un RR de 0,47 (IC95%: 0,24 a 0,92) para el requerimiento de VM en adultos, pero en niños fue de 1,68 (IC95%: 1,25 a 2,25). Hubo una alta heterogeneidad estadística.

**Conclusiones.:**

Las variantes axonales tienen en promedio mayor tiempo de VM y de hospitalización, en total y por subgrupos. Se observó un mayor requerimiento de VM para las variantes axonales en niños; mientras que en los adultos fue menor.

## INTRODUCCIÓN

El síndrome de Guillain-Barré (SGB) es una enfermedad autoinmune caracterizada por una debilidad motora progresiva y una disminución o ausencia de reflejos. Los músculos respiratorios pueden verse comprometidos, lo que resulta en la necesidad de ventilación mecánica (VM), que ocurre en el 20-30% de los pacientes ^1^^,^^2^.

El SGB incluye la variante desmielinizante, llamada polirradiculoneuropatía desmielinizante inflamatoria aguda (AIDP), que causa síntomas sensoriales y motores, disfunción autonómica, dolor y déficit de nervios craneales; y por otro lado la variante axonal, que incluye la neuropatía axonal motora aguda (AMAN), que provoca únicamente síntomas motores; neuropatía axonal sensitivo-motora aguda (AMSAN), que se presenta con síntomas tanto sensitivos como motores; neuropatía sensorial aguda (ASAN), que causa desmielinización de los nervios periféricos; y el síndrome de Miller-Fisher (SMF), que se manifiesta con oftalmoplejía, ataxia y arreflexia ^3^^,^^4^.

La incidencia a nivel mundial es de 0,89-1,89 casos por cada 100.000 habitantes al año, siendo los niños los de mejor pronóstico ^5^^-^^7^. En Europa y América del Norte, la AIDP representa el 90% de los casos. Sin embargo, en Asia, América Central y del Sur, las variantes axonales son las más comunes, con un 30-40% de los casos ^8^.

La necesidad de VM implica el ingreso a la Unidad de Cuidados Intensivos (UCI) ^9^. Los pacientes con un tiempo de intubación mayor a 2 meses mostraron un período más largo (hasta 6 meses) para recuperar la capacidad de caminar ^10^. La mortalidad en personas que requieren VM varía entre 8,3-20% ^11^^,^^12^.

La AIDP tiene mejor pronóstico en comparación con la variante axonal, siendo el requerimiento de VM del 10% y 38%, respectivamente. AMAN y AIDP tienen una recuperación más rápida en comparación con AMSAN ^13^. Este último se manifiesta con síntomas severos, uso prolongado de VM ^14^ y mayor tiempo de hospitalización ^15^. Sin embargo, los hallazgos entre las publicaciones son inconsistentes, lo que destaca la necesidad de analizar sistemáticamente los datos, ya que sólo hay revisiones narrativas.

Aunque hay un porcentaje importante de pacientes que requieren VM, desarrollan secuelas permanentes o mueren, no está claro qué variante se asocia más con resultados adversos. Por lo tanto, es valioso recopilar información sobre los desenlaces adversos asociados con cada variante para cuantificar los recursos adicionales necesarios para estos pacientes y desarrollar planes de contingencia, especialmente en situaciones de brote. Por lo anteriormente mencionado, el objetivo del estudio fue determinar la frecuencia y duración de los desenlaces adversos (requerimiento y tiempo en VM, ingreso y tiempo en UCI, hospitalización y tiempo de hospitalización, muerte, discapacidad) de las variantes axonales del SGB en comparación con la variante aguda desmielinizante en pacientes de todas las edades.

**MENSAJES CLAVE**

Motivación para el estudio. No está claro qué variante del síndrome de Guillain-Barré está más asociada con desenlaces adversos.

Principales hallazgos. Las variantes tuvieron un mayor tiempo en VM (7,42 días más), y de hospitalización (3,11 días más). Por subgrupos, se observó que la variante axonal en niños presentaba un mayor uso de VM; mientras que, en adultos, la variante desmielinizante un mayor número de pacientes en VM.

Implicancias. Los resultados podrán ser utilizados para la implementación de nuevas políticas de salud pública, permitiendo al personal de salud tener un mayor conocimiento sobre los pronósticos de cada variante y los recursos necesarios para enfrentar futuros brotes.

## MATERIALES Y MÉTODOS

### Diseño del estudio

Se realizó una revisión sistemática donde se evaluó la frecuencia de los desenlaces adversos de las variantes axonales del SGB (AMAN, AMSAN, ASAN, SMF) en comparación con la variante desmielinizante aguda (AIDP), estratificada para pacientes menores y mayores de 18 años. Para la revisión sistemática de estudios observacionales se utilizó la metodología Cochrane, y se aplicó la lista de verificación PRISMA (Preferred Reporting Items for Systematic review and Meta-Analysis) ^16^^,^^17^. La búsqueda y selección de artículos se realizó entre julio y septiembre de 2020, y una actualización en marzo de 2021.

### Criterios de elegibilidad

La población fueron estudios observacionales que incluyen pacientes con el diagnóstico de SGB, según el diagnóstico clínico que aparece en las historias clínicas, o según criterios diagnósticos como Brighton, Asbury y Cornblath, National Institute of Neurologic Disorders and Stroke (NINCDS), Hadden, Ho, ICD-10 y electrofisiológico. Las variantes axonales se consideraron como exposición, en comparación con las desmielinizantes. Los desenlaces considerados fueron requerimiento y tiempo de VM, ingreso y tiempo de UCI, hospitalización y tiempo de hospitalización, muerte y discapacidad. Se incluyeron estudios observacionales, y se excluyeron ensayos clínicos, revisiones sistemáticas, revisiones narrativas y guías de práctica clínica. Ningún estudio fue excluido por filtros. Cuando no se pudo acceder a los artículos o encontrarlos en un idioma diferente al inglés, español o portugués, se contactó al investigador, si no se obtenían fueron excluidos.

### Estrategia de búsqueda y selección de estudios

Se realizó una búsqueda bibliográfica en MEDLINE/Pubmed, LILACS/SciELO, Cochrane, Scopus, Web of Science y Google Scholar (Material Suplementario). Los artículos seleccionados se registraron en el gestor de referencias de Zotero, donde se eliminaron los artículos duplicados.

### Extracción de datos

De cada estudio seleccionado se extrajeron por duplicado: número de participantes, número de expuestos a cada variante de SGB, medidas de desenlace para cada exposición y medidas de asociación con sus intervalos de confianza (IC) del 95%. Los desenlaces fueron: numéricos (tiempo de VM, tiempo de UCI, tiempo de hospitalización, puntuación de discapacidad) y dicotómicos (ingreso a UCI, requerimiento de VM, muerte, discapacidad). Para los desenlaces numéricos, se colocaron medias con desviación estándar (DE). Si se reportaban medianas con rangos intercuartílicos (RIQ), se consideró la mediana como media y el RIQ se dividió por 1,35 para estimar la DE siguiendo el Manual Cochrane ^17^. La medida de asociación fue la diferencia de medias (DM) con su IC del 95%; en caso de no informarse, se calculó la DM, sin colocar el IC del 95%. Para los desenlaces categóricos, se utilizaron las frecuencias absolutas de cada desenlace según la exposición, y como medidas de asociación se utilizaron los riesgos relativos (RR) con su IC del 95%; en caso de no reportarse, se calculó el RR, sin colocar el IC del 95%.

### Evaluación del riesgo de sesgo

Cada artículo contó con una evaluación por duplicado de la calidad metodológica mediante la Escala Newcastle-Ottawa (NOS) para cohortes y para estudios de casos y controles. La versión de cohorte se utilizó para estudios transversales o series de casos. La NOS tiene tres dominios: selección, comparabilidad y exposición ^18^, con puntuaciones de 0 a 9. Los estudios con puntuaciones inferiores a 7 puntos se consideraron con alto riesgo de sesgo. Para los resultados con al menos 9 artículos, se realizó un gráfico en embudo de Egger para evaluar los sesgos de publicación. La probabilidad de sesgo de publicación fue alta cuando el valor p fue ≤0,05.

### Síntesis de datos

Para los resultados que tenían 3 o más estudios incluidos, se realizó un metaanálisis utilizando el programa Review Manager 5.3 (RevMan 5.3, Copenhague, The Cochrane Collaboration). Se utilizaron modelos de efectos aleatorios con varianzas inversas como forma de ponderación. Se obtuvieron combinando todos los estudios para ese desenlace, y en diferentes subgrupos: estudios que combinan niños y adultos, sólo niños y sólo adultos.

Se utilizó la prueba I^2^ y la prueba chi^2^ para estimar la heterogeneidad estadística de los estudios. Valores de I^2^ iguales o superiores al 75%, indicaron heterogeneidad alta, y baja cuando fueron inferiores al 25%.

### Consideraciones éticas

Las revisiones sistemáticas utilizan información procedente de bases de datos de libre acceso y textos ya publicados que no contienen identificadores individuales de los participantes. No se incluyeron estudios con violaciones éticas o retractaciones. No se excluyeron estudios por motivos de sexo, raza, religión, origen o idioma. El protocolo de esta revisión sistemática fue aprobado por el Comité de Ética de la Facultad de Ciencias de la Salud de la Universidad Peruana de Ciencias Aplicadas (UPC) con el código FCS-CEI/216-07-20 y FCS-CEI/345-05-21. Además, fue registrado en la base de datos internacional PROSPERO con el código CRD42020198653.

## RESULTADOS

Luego de aplicar los términos de búsqueda se obtuvieron de la base de datos 10960 artículos. Después de eliminar los duplicados, quedaron 3116. Se excluyeron 2996 artículos por título y resumen, y 120 se dejaron para revisión del texto completo. Se eliminaron 15 por no presentar la exposición, cuatro por no presentar los desenlaces, 33 por no analizar los resultados por exposición, 18 porque no se pudo acceder al texto completo y cuatro estaban en un idioma distinto al español, inglés o portugués.

Se incluyeron 46 artículos, publicados entre 2003 y 2020. Veintisiete fueron desarrollados en Asia, siete en Europa, cinco en Turquía, cinco en América Latina y dos en África. Trece fueron cohortes prospectivas, 26 cohortes retrospectivas, cinco series de casos, un estudio analítico transversal y un caso-control.

Hubo 4585 pacientes de ambos sexos: 1780 de las variantes axonales y 2805 de la desmielinizante. Diecisiete artículos consideraron la variante axonal en su conjunto, 14 reportaron AMAN por separado, 14 reportaron AMSAN por separado, uno reportó ASAN y nueve reportaron SMF. En total, 12 artículos eran sólo para adultos, 21 sólo para niños y 13 incluían a ambos grupos.

Respecto a los desenlaces, 34 mencionaron el requerimiento de VM como variable discreta, y 8 midieron el tiempo de VM como variable continua en días. Sólo 1 reportó el ingreso a UCI como variable discreta, cinco reportaron el tiempo en UCI como variable continua en días y 17 reportaron el tiempo de hospitalización como variable continua en días. En cuanto a la discapacidad posterior al evento, 13 reportaron la escala de Hughes como variable continua, y 13 dicotomizaron la escala utilizando diferentes puntos de corte. Diez estudios reportaron la proporción de muertes como una variable discreta; 7 informaron muertes durante la hospitalización, un estudio a los tres meses, otro a los seis meses y uno durante un período de seguimiento fluctuante.

Tras la aplicación de la NOS, se encontraron 31 artículos con riesgo bajo, siendo un estudio de casos y controles. Se realizaron gráficos en embudo para el tiempo de hospitalización, discapacidad como variable dicotómica, necesidad de VM y muerte. Estos no demostraron posibles sesgos de publicación para discapacidad o muerte. Para el tiempo de hospitalización, los estudios no se mantuvieron dentro de los límites del embudo, sino que se distribuyeron simétricamente en ambos lados. Para la VM, parece haber un sesgo hacia publicaciones con RR<1,0, que favorece las variantes axonales, en estudios realizados en adultos.

Veinticuatro estudios tuvieron desenlaces numéricos, 11 reportaron medias con DE, 10 reportaron medias sin medidas de dispersión, y tres reportaron medianas con RIQ, que se transformaron en DE. Artículos sin medidas de dispersión, la DE combinada del resto de estudios de su grupo correspondiente, fueron usados en el metaanálisis, siguiendo lo descrito por Ma *et al*^19^. Sólo uno reportó la DM con su IC del 95%, el resto fueron calculados mediante el programa Review Manager 5.4 y aparecen en los “Forest Plots” correspondientes. 42 de 46 estudios reportaron un desenlace dicotómico con frecuencias absolutas. Sólo dos estudios reportaron medidas de asociación con su IC del 95%, un artículo reportó odds ratio (OR) y otro RR. Los metaanálisis se realizaron para cada uno de los desenlaces, excepto el ingreso a UCI por tener un solo estudio.

Para el tiempo en VM (Figura 1), la DM fue 2,02 (IC 95%: -5,19 a 9,23) días más para las variantes axonales en estudios que combinaron niños y adultos; mientras que en los estudios con sólo niños fue 11,10 días (IC 95%: 2,21 a 19,99). Esto no fue significativo para ningún subgrupo. La combinación de los 8 estudios mostró un tiempo de 7,42 días (IC 95%: 0,36 a 14,48) en las variantes axonales, siendo significativo, pero con alta heterogeneidad estadística (I^2^ = 97%).

**Figura 1:** *Forest Plot* para el tiempo de ventilación mecánica de las variantes axonales en comparación con la variante desmielinizante en pacientes con Síndrome de Guillain-Barré menores.

IC: intervalo de confianza; AIDP: polirradiculoneuropatía desmielinizante inflamatoria aguda; UCI: Unidad de Cuidados Intensivos; DE: desviación estándar.

En cuanto a la duración de la estancia en UCI (Figura 2), fue 42,4 días (IC 95%: -26,35 a 111,22) más para las variantes axonales solo en adultos; mientras que en sólo niños la DM fue 3,66 días (IC 95%: -15,48 a 22,80); sin ser significativo. La DM de 5 estudios mostró un tiempo de 19,23 días (IC95%: -22,27 a 60,72) en las variantes axonales, sin ser significativo, y una alta heterogeneidad estadística (I^2^ = 99%).

**Figura 2:** *Forest Plot* para el tiempo en Unidad de Cuidados Intensivos de las variantes axonales en comparación con la variante desmielinizante en pacientes con Síndrome de Guillain-Barré.

IC: intervalo de confianza; AIDP: polirradiculoneuropatía desmielinizante inflamatoria aguda; DE: desviación estándar.

Para el tiempo de hospitalización (Figura 3), la DM fue 0,30 días (IC 95%: -4,85 a 5,46) para las variantes axonales en los estudios que combinaron niños y adultos, sin ser significativo; mientras que en estudios de sólo adultos fue 4,21 días más (IC 95%: 0,76 a 7,66), y en sólo niños fue 5,05 días (IC 95%: 1,10 a 9,00); siendo este último significativo. La combinación de 17 estudios mostró una DM de 3,11 días (IC 95%: 0,73 a 5,49) en las variantes axonales, sin ser significativo, y con una heterogeneidad estadística media (I^2^=45%).

**Figura 3:** *Forest Plot* para el tiempo de hospitalización de las variantes axonales en comparación con la variante desmielinizante en pacientes con Síndrome de Guillain-Barré.

IC: intervalo de confianza; AIDP: polirradiculoneuropatía desmielinizante inflamatoria aguda; DE: desviación estándar.

La puntuación de discapacidad de Hughes (Figura 4), mostró una DM de -0,01 puntos (IC 95%: -0,48 a 0,47) para las variantes axonales en estudios que combinaron niños y adultos; mientras que en sólo niños fue 0,23 puntos (IC 95%: -0,16 a 0,63); sin ser ambos significativos. La combinación de seis estudios mostró 0,07 (IC 95%: -0,26 a 0,40) puntos más en variantes axonales, lo cual no fue significativo, con una heterogeneidad estadística media (I^2^=70%).

**Figura 4:** *Forest Plot* para medidas de dispersión de discapacidad (escala de Hughes) de las variantes axonales en comparación con la variante desmie-linizante en pacientes con Síndrome de Guillain-Barré

IC: intervalo de confianza; AIDP: polirradiculoneuropatía desmielinizante inflamatoria aguda; DE: desviación estándar.

Al analizar la discapacidad como variable dicotómica (Figura 5), los estudios que combinaron niños y adultos, los subtipos axonales tuvieron una probabilidad de 1,25 (IC 95% 0,65 a 2,38) veces de desarrollar discapacidad; mientras que en adultos hubo un RR de 1,23 (IC 95%: 0,77 a 1,98), no siendo significativo. En estudios en niños, el RR fue significativo con 1,22 (IC 95%: 1,05 a 1,40). En total, nueve estudios mostraron que las variantes axonales tuvieron un RR de 1,17 (IC 95%: 0,94 a 1,46), con heterogeneidad estadística media (I^2^=55%).

**Figura 5:** *Forest Plot* para discapacidad (variable dicotómica) de las variantes axonales en comparación con la variante desmielinizante en pacientes con Síndrome de Guillain-Barré.

IC: intervalo de confianza; AIDP: polirradiculoneuropatía desmielinizante inflamatoria aguda; DE: desviación estándar.

Para el requerimiento de VM (Figura 6), los estudios que combinaron niños y adultos, las formas axonales tuvieron una probabilidad de 1,43 (IC 95%: 1,05 a 1,95) veces de requerir VM; y en sólo niños fue de 1,68 veces (IC 95%: 1,25 a 2,25), siendo ambos significativos. La asociación fue inversa en estudios de sólo adultos, con un RR de 0,44 (IC 95%: 0,23 a 0,86), también significativo.

**Figura 6:** *Forest Plot* para requerimiento de ventilación mecánica de las variantes axonales en comparación con la variante desmielinizante en pacientes con Síndrome de Guillain-Barré.Favours axonalFavours AIDP0,010,1101001.

Los 36 estudios combinados mostraron que las variantes axonales tuvieron un RR de 1,13 (IC 95%: 0,86 a 1,48) con una heterogeneidad estadística media (I2 = 58%), sin ser significativo.

Cuando se analizó la muerte (Figura 7), los estudios que combinaron niños y adultos, las variantes axonales tuvieron una probabilidad de morir de 2,17 (IC 95%: 1,00 a 4,70), lo cual fue marginalmente significativo. En solo adultos, hubo un RR de 0,29 (IC 95%: 0,06 a 1,48), y en sólo niños fue de 1,89 (IC 95%: 0,30 a 11,66), ambos no significativos. Los 10 estudios combinados mostraron que los subtipos axonales tuvieron un RR de muerte de 1,54 (IC 95%: 0,80 a 2,95), sin ser significativo, con una heterogeneidad estadística media (I^2^=58,9%).

**Figura 7:** Forest Plot para muertes por las variantes axonales en comparación con la variante desmielinizante en pacientes con Síndrome de Guillain-Barré.

Los metaanálisis y los Forest Plot se repitieron para los desenlaces donde se encontraron diferencias significativas en total o en algún subgrupo, pero dando un peso del 0% a los estudios que obtuvieron una puntuación <7 en NOS.

Para tiempo en VM, la DM para niños (10,20; IC 95%: -0,49 a 20,89) y en total (5,57; IC95%: -2,58 a 13,71) perdió significancia estadística al aumentar el IC; sin embargo, la direccionalidad hacia un mayor número de días se mantuvo con las variantes axonales. Para el tiempo de hospitalización, la DM siguió siendo significativa para los adultos (3,03; IC 95%: 0,50 a 5,56), pero se redujo para los niños (3,84; IC 95%: -0,12 a 7,80) como total (2,05; IC 95%: -0,11 a 4,22); sin embargo, la direccionalidad hacia una estancia hospitalaria más prolongada se mantuvo con las variantes axonales.

Para el resultado dicotómico de discapacidad, el RR se mantuvo sin cambios y fue significativo para los niños (1,22; IC 95%: 1,05 a 1,40), y no significativo en total (1,17; IC95%: 0,94 a 1,46). Para el requerimiento de VM, la asociación en niños siguió siendo significativa (0,61; IC 95% 1,17 a 2,34), y el análisis con todos los artículos se volvió significativo (1,39; IC 95% 1,07 a 1,80). Para muerte, no hubo cambios en la significancia o direccionalidad de las asociaciones (1,22; IC 95%: 0,61 a 2,42).

## DISCUSIÓN

Hasta donde sabemos, esta es la primera revisión sistemática que evalúa las diferencias en los desenlaces clínicos según el tipo de SGB. Las variantes axonales tuvieron 7,42 días más en VM (IC 95%: 0,36 a 1,48), y mayor tiempo de hospitalización con 3,11 días (IC 95%: 0,73 a 5,49). Se observó un 17% más de aparición de discapacidad (RR 1,17; IC 95%: 0,94 a 1,46), aunque no significativo. No hubo diferencias estadísticamente significativas para los otros desenlaces. En algunos casos, se observaron diferencias en la direccionalidad entre los estudios que incluyeron sólo niños, sólo adultos, y ambos grupos.

En cuanto al tiempo de VM, las variantes axonales requieren un uso más prolongado en comparación con las desmielinizantes. Sin embargo, dos artículos reportan que el subtipo desmielinizante permanece en VM por más días, en comparación con la axonal. El primer estudio, Gupta 2019 ^20^, incluyó únicamente a niños donde el tiempo de VM en el subtipo axonal fue de 30 días, mientras que en el desmielinizante fue de 32 días. El segundo, Shafqat 2006 ^21^, incluyó niños y adultos; reportando que la variante axonal tiene 19 días en VM y 27 días para la desmielinizante. El mayor tiempo de VM en el subtipo axonal puede deberse a que presenta más discapacidades, aumentando la posibilidad de compromiso de los músculos respiratorios. Cuando se analiza por subgrupos, los niños tienen un mayor requerimiento de VM en comparación con los adultos. La variante axonal en niños tiene una presentación grave y por tanto requiere un mayor uso de VM.

Las variantes axonales requieren una estancia más prolongada en la UCI en comparación con las desmielinizantes, aunque estas diferencias no fueron significativas. Varios estudios reportan resultados similares, pero algunos muestran el efecto contrario, como Estrade 2019 ^22^. Este estudio realizado en niños informa una estancia promedio de 3 días en la UCI para el subtipo axonal y de 18 días para el desmielinizante. Según los resultados del presente estudio, en la variante axonal, el tiempo en la UCI es mayor en comparación con el tiempo de VM. Esta discrepancia puede deberse a que los pacientes pueden ser ingresados en la UCI por razones distintas a la VM ^23^, pero estos no especifican la razón del ingreso a la UCI.

El tiempo de hospitalización aumentó estadísticamente en la variante axonal. Sin embargo, algunos artículos reportan discrepancias. Cheng 2003 ^24^, que evalúa pacientes de todas las edades, indica un promedio de 15,9 días de hospitalización para el tipo axonal y 25,4 días para el desmielinizante. Shafqat 2006 ^21^ reportó 14 días de hospitalización para el axonal en comparación con 15 días para el desmielinizante. Peña 2015 ^25^ fue el único que difirió de los estudios en adultos, donde el subtipo axonal estuvo hospitalizado por 9,8 días y el desmielinizante por 14 días. Sólo 2 artículos realizados únicamente en niños reportan diferencias. Primero es Akbayram 2011 ^26^ donde la variante axonal tuvo un tiempo medio de hospitalización de tres días y el desmielinizante fue de diez días, y Estrade 2019 ^22^ que reportó 11,4 días de hospitalización para el axonal en comparación con 12,6 días para el desmielinizante. Esta diferencia puede deberse al retraso en el diagnóstico del SGB, lo que lleva a una progresión de la enfermedad y la gravedad de cada variante, siendo la axonal la que requiere el mayor tiempo de hospitalización debido a más complicaciones.

En la variante axonal, el tiempo de hospitalización es menor comparado con la estancia en la UCI y la VM. Esto podría deberse al hecho de que no todos los pacientes son ingresados en la UCI. Por lo tanto, si el tiempo de hospitalización en pacientes que no son ingresados en la UCI es más corto en las variantes axonales, el efecto combinado en la prolongación sería menor. Desafortunadamente, los estudios no reportan tiempos de hospitalización estratificados según la admisión a la UCI o no. Otra posibilidad es que después de salir de la UCI, los pacientes sean transferidos directamente a institutos especializados, reduciendo el tiempo de hospitalización.

En cuanto al requerimiento de VM, se observó un efecto diferenciado entre niños y adultos. Los estudios que incluyeron niños y adultos encontraron un 43% más de uso de VM; mientras que en los niños solo se observó un 68% más de uso, ambos estadísticamente significativos. En contraste, la investigación realizada solo en adultos mostró un 53% menos de uso de VM, también significativo. Al combinar estos valores, el resultado no muestra efecto. Podemos concluir que los resultados en cuanto al requerimiento de VM son diferentes según la edad. Aunque en niños, las variantes axonales aumentan la disfunción ventilatoria, en adultos, las desmielinizantes tienen mayor riesgo de VM. Esto podría deberse a que los niños a menudo presentan la variante axonal, que progresa rápidamente, en comparación con los adultos, por lo tanto, desarrollan disfunción respiratoria y la necesidad de VM. Sin embargo, no se encontraron estudios que comparen el requerimiento de VM entre niños y adultos.

La discapacidad fue evaluada según la escala de Hughes, como una variable continua y dicotómica, aunque con diferentes puntos de corte. Cuando se evaluó como una variable continua, no se observaron diferencias. Cuando se evaluó de forma dicotomizada, hubo una tendencia hacia una mayor ocurrencia de discapacidad en las formas axonales. Todos los estudios coinciden en esto, excepto Netto 2011 ^(^^27^ y Dourado 2012 ^28^, que reportaron más discapacidad en AIDP. Aunque el valor ponderado mostró más discapacidad en las formas axonales, esto no fue significativo. La discrepancia entre ambas formas de medición puede deberse a que, aunque los puntajes son similares en promedio; en los casos axonales, más pacientes tuvieron valores que pasaron el punto de corte. Esto podría interpretarse que, aunque las formas axonales causan más discapacidad; cuando estas ocurren en AIDP, tienden a dar puntajes más altos, es decir, son más graves. Un estudio que compare los puntajes de pacientes ya clasificados como discapacitados podría mostrarnos esto más claramente.

La letalidad fue mayor en las variantes axonales en estudios realizados solo en niños, o combinados niños y adultos; mientras que hubo más muertes en AIDP cuando los estudios se realizaron solo en adultos. Al combinar los diferentes subgrupos, los efectos se cancelan entre sí, resultando en una asociación no significativa. Nos encontramos ante un caso de interacción, en el cual la edad cambia la direccionalidad de la asociación. Debido al pequeño número de estudios por subgrupo y al amplio IC, no podemos llegar a una conclusión clara, pero parece que las variantes axonales aumentan las muertes en niños, mientras que AIDP tiene una mayor letalidad en adultos. Los niños con variantes axonales tienen una enfermedad más grave y tienen mayor riesgo de afectación respiratoria y alteración hemodinámica, por lo cual se recomienda la hospitalización o estancia en la UCI ^29^^,^^30^. Es evidente la contradicción, ya que la literatura describe que los niños tienen menos secuelas, mejor pronóstico y menor mortalidad que los adultos ^31^. Se cree que las diferencias entre las variantes de SGB en niños y adultos se deben a sus antecedentes y factores de riesgo, que afectan la gravedad y su pronóstico ^29^.

Los sesgos de selección pueden ocurrir si los estudios con ciertos resultados han sido sistemáticamente excluidos, pero los estudios excluidos no estaban disponibles o no contenían la información requerida, y por lo tanto no pudieron haber sido excluidos basándose en los resultados que informan. Otro sesgo de selección podría ocurrir si se favorece una definición de SGB sobre otra. Para evitar esto, se han utilizado casi todos los criterios diagnósticos disponibles. Aunque esto genera mayor heterogeneidad metodológica entre los estudios, el beneficio supera el riesgo. Para definir el subtipo de SGB, no todos los estudios incluyen estudios electromiográficos, en cuyo caso la exposición y el comparador no podrían clasificarse correctamente. Se incluyeron todos los estudios incluso si no mencionaban si realizaron electromiografía. Al no excluir estos, se asegura que los estudios realizados en sistemas de salud deficientes entren en la revisión.

Excluimos ensayos clínicos y, revisiones sistemáticas, revisiones narrativas y guías de práctica clínica porque los resultados podrían ser modificados debido a la intervención. Nuestra búsqueda bibliográfica no utilizó la base de datos EMBASE, debido a no tener acceso gratuito a ella. No anticipamos que esto genere un gran sesgo de selección, ya que se utilizaron otras seis bases de datos y un gran número de publicaciones.

La evaluación de las publicaciones y la extracción de datos no fueron realizadas por expertos en SGB ni por expertos metodológicos, lo que podría generar limitaciones. Para minimizar esto, se consultaron definiciones y clasificaciones de SGB con un con un experto metodológico. Un tercio de los estudios tenía problemas potenciales de calidad, pero se realizó un análisis de sensibilidad incluyendo solo estudios con alta calidad metodológica. Se encontró alta heterogeneidad estadística y/o metodológica para algunos de los resultados, lo cual siempre se ha reportado.

Haber estratificado los estudios por edad, nos permitió evaluar el resultado de cada variante por subgrupos y encontrar interacciones importantes según la edad, en dos de los resultados evaluados.

Los resultados encontrados pueden ser utilizados para la implementación de nuevas políticas de salud pública, permitiendo que el personal de salud tenga un mayor conocimiento sobre los pronósticos de cada variante y los recursos necesarios para enfrentar futuros brotes. Se puede llevar a cabo un diagnóstico temprano y un tratamiento adecuado para reducir el tiempo de hospitalización, el requerimiento de VM, la discapacidad y la muerte. Finalmente, se sugiere estudiar estos resultados categorizados por grupos de edad, ya que nuestra revisión detectó una interacción importante de la edad en el efecto que las variantes tienen sobre los resultados clínicos.

En conclusión, las variantes axonales tuvieron un tiempo más prolongado en VM con una diferencia significativa de 7,4 días más y un tiempo de hospitalización más largo con 3,1 días más. El tiempo en UCI fue más largo, pero sin diferencia significativa. La discapacidad fue un 17% mayor en las variantes axonales, sin llegar a ser significativa. No hubo diferencias estadísticamente significativas cuando se evaluó como una variable numérica.

El requerimiento de VM entre las variantes axonales no fue significativo cuando se analizó en conjunto. Sin embargo, por subgrupos, se observó más VM para las variantes axonales en niños; mientras que, en adultos, hubo más VM con la variante desmielinizante. Se observó una tendencia similar para la muerte sin llegar a ser significativa.
